# Preparation of imidazo[1,2-*a*]-*N*-heterocyclic derivatives with *gem*-difluorinated side chains

**DOI:** 10.3762/bjoc.13.208

**Published:** 2017-10-10

**Authors:** Layal Hariss, Kamal Bou Hadir, Mirvat El-Masri, Thierry Roisnel, René Grée, Ali Hachem

**Affiliations:** 1 Laboratory for Medicinal Chemistry and Natural Products, Lebanese University, Faculty of Sciences (1) and PRASE-EDST, Hadath, Beirut, Lebanon; 2 American University of Beirut, Department of Chemistry, Beirut 11-0236, Lebanon; 3 Université de Rennes 1, Institut des Sciences Chimiques de Rennes, CNRS UMR 6226, Avenue du Général Leclerc, 35042 Rennes Cedex, France

**Keywords:** aerobic oxidative coupling, imidazo[1,2-*a*]-*N*-heterocycles, *gem*-difluoroalkyl derivatives, propargylic fluorides

## Abstract

Using an aerobic oxidative coupling, different new imidazo[1,2-*a*]-*N*-heterocycles with *gem*-difluroroalkyl side chains have been prepared in fair yields by the reaction of *gem*-difluoroenones with aminopyridines, -pyrimidines and -pyridazines. Condensed heterocycles of this type play an important role as key core structures of various bioactive compounds. Further, starting with a chloroimidazopyridazine derivative, Pd-catalyzed coupling reactions as well as nucleophilic substitutions have been performed successfully in order to increase the molecular diversity.

## Introduction

Nitrogen-containing heterocyclic compounds are frequently found in bioactive naturally occurring compounds, as well as in the synthetic pharmacopeia. Imidazo[1,2-*a*]pyridine is an important heterocyclic system present in many molecules featuring diverse biological activities, such as antiviral, antimicrobial, antitumor, anti-inflammatory, antiparasitic, hypnotic, etc. [1–5]. It is recognized as a key scaffold due to its broad occurrence in a number of drug candidates and drugs, such as zolpidem [6] (**1a**, used in the treatment of insomnia), and alpidem [6] (**1b**, an anxiolytic agent). Some imidazopyridine derivatives also act as β-amyloid formation inhibitors, GABA and benzodiazepine receptor agonists, and cardiotonic agents [7–10]. Further, the biological activities of imidazo[1,2-*a*]pyridines proved to be strongly depending upon the nature of substituents at C2 and C3 positions. For instance, the 3-aroylimidazo[1,2-*a*]pyridines **1c** demonstrated also good anticancer properties [11–12], while imidazo[1,2-*a*]pyrimidines **2** are also known for their antituberculosis activity [13], and imidazopyridazine **3** acts as a sirtuin modulator [14].

On the other hand, the incorporation of fluorine or fluorinated groups into organic molecules has been widely recognized as a general strategy toward drug development in pharmaceutical research. This is connected to fluorine's electronegativity, size, and lipophilicity [15–16], which can strongly improve the biological properties of molecules through, for instance, increase of metabolic stability and bioavailability for many drugs and pharmacological tools. So, the preparation of fluorinated molecules is a very attractive research area for organic and medicinal chemists [17–20].

**Figure 1:**
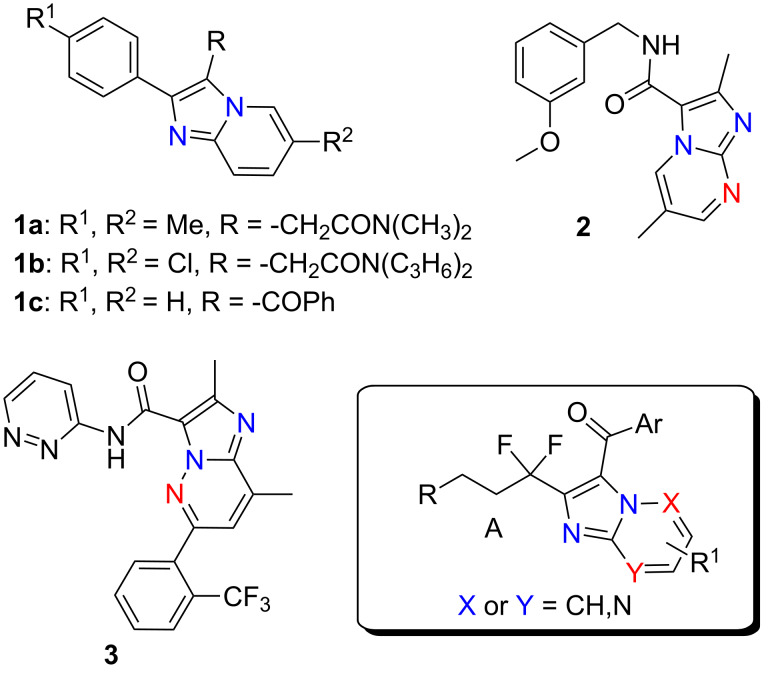
Representative examples of bioactive imidazo[1,2-*a*]pyridines, imidazo[1,2-*a*]pyrimidines, imidazopyridazines and our target molecules.

Our research program aims to synthesize new fluorinated molecules based on the easy access and the versatility of fluorinated propargylic derivatives [21]. Thus, taking into account the known biological properties of the imidazo-fused *N*-heterocycles, we became interested in the preparation of new derivatives of this type possessing *gem*-difluorinated side chains as indicated in Figure 1. Such new fluorinated heterocycles could be of interest for bioorganic and medicinal chemistry studies.

**Scheme 1:**
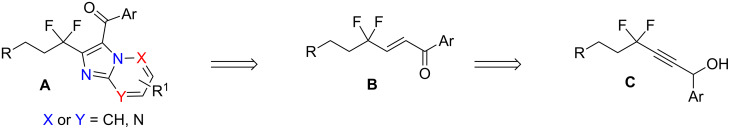
Retrosynthetic scheme for the preparation of our target molecules **A**.

Several synthetic approaches for imidazopyridines are available, but only a few examples have been reported to date for the construction of this scaffold with introduction of fluorine [22], trifluoromethyl [23] or trifluoroethyl groups [24]. Herein, we report the synthesis of imidazo[1,2-*a*]pyridines, imidazo[1,2-*a*]pyrimidines, and imidazopyridazines with fluorinated side chains following an efficient strategy developed by Hajra et al. [25]. This methodology, developed for the synthesis of 3-aroylimidazopyridines, involves a copper(II) acetate-catalyzed aerobic oxidative amination and it proceeds through a tandem Michael addition followed by an intramolecular oxidative amination. Therefore, our target molecules **A** could be synthesized by the oxidative coupling of 2-aminopyridines with α,β-unsaturated ketones **B**, themselves easily accessible from *gem*-difluoropropargylic alcohols **C** through a base-mediated isomerization process (Scheme 1) [26–27].

## Results and Discussion

**Scheme 2:**
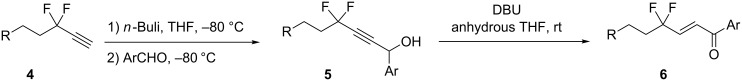
Synthesis of enones **6** with a *gem*-difluoroalkyl side chain.

The required propargylic alcohols **5a**–**e** (type **C**, Scheme 1) were obtained in 27–73% yields by reaction of the lithium salt of the easily accessible *gem*-difluoro propargylic derivatives **4** [28] with aromatic aldehydes. Then, the DBU-mediated isomerization afforded the desired enones **6a**–**e** in 21–66% yields (Scheme 2 and Table 1).

**Table 1:** Synthesis of enones **6a**–**e**.

Entry	R	Ar	**5**: yield (%)	**6**: yield (%)

1	Ph	Ph	**5a** (73%)	**6a** (62%)
2	Ph	*o*-PhBr	**5b** (70%)	**6b** (60%)
3	Ph	2-naphthaldehyde	**5c** (71%)	**6c** (50%)
4	Ph	*p*-anisaldehyde	**5d** (27%)	**6d** (21%)
5	CH_2_OBn	Ph	**5e** (65%)	**6e** (66%)

**Scheme 3:**
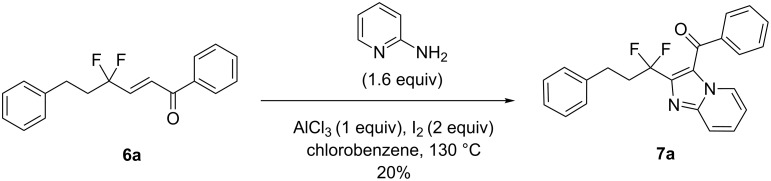
Synthesis of **7a**.

For the synthesis of the desired nitrogen heterocycles, we started our study by reacting **6a** with 2-aminopyridine in the presence of AlCl_3_ and I_2_ under an O_2_ atmosphere [29]. However, only a poor yield was obtained (20%, Scheme 3).

Then, we found that Cu(OAc)_2_·H_2_O (10 mol %) and 1,10-phenanthroline (10 mol %) in chlorobenzene at 160 °C under an O_2_ atmosphere, following the conditions recently reported by Hajra et al. [25], gave **7a** in 62% yield (Table 2, entry 1). Having these optimized conditions in hand, and to explore the substrate scope, different substituted 2-aminopyridines were successfully employed to afford the tandem oxidative cyclization products **7** in 32–65% yields. On the other hand, enones **6** with two different R groups (Table 2, entries 1, 2, 3, 6, 8, and 9) were well tolerated under the optimized conditions affording the tandem products **7** in fair to moderate yields, although lower yields were obtained in the cases of **7j** and **7g** (32% and 36%, respectively). Moreover, two other important heterocyclic frameworks, imidazo[1,2-*a*]pyrimidines **7d** and imidazo[1,2-*b*]pyridazines **7e**, have been synthesized by the same method albeit in slightly decreased yields (Table 2, entries 4 and 5).

**Table 2:** Preparation of different imidazo[1,2-*a*]-*N*-heterocyclic derivatives.

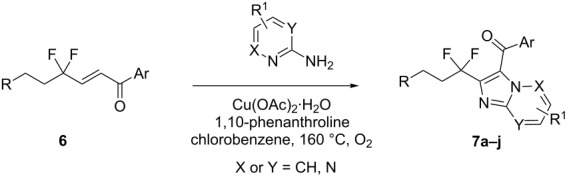								

Entry	Product	Ar	R	R^1^	X	Y	Time	Yield (%)

1	**7a**	Ph	Ph	H	CH	CH	25 h	62
2	**7b**	Ph	Ph	7-Me	CH	CH	20 h	60
3	**7c**	Ph	Ph	6-Br	CH	CH	46 h	60
4	**7d**	Ph	Ph	H	CH	N	30 h	57
5	**7e**	Ph	Ph	6-Cl	N	CH	24 h	53
6	**7f**	Ph	-CH_2_OBn	H	CH	CH	33 h	55
7	**7g**	Ph	-CH_2_OBn	H	CH	N	29 h	36
8	**7h**	*o*-BrPh	Ph	H	CH	CH	4 h	65
9	**7i**	2-naphthaldehyde	Ph	H	CH	CH	3.5 h	59
10	**7j**	*p*-MeOPh	Ph	H	CH	CH	6 h	32

**Figure 2:**
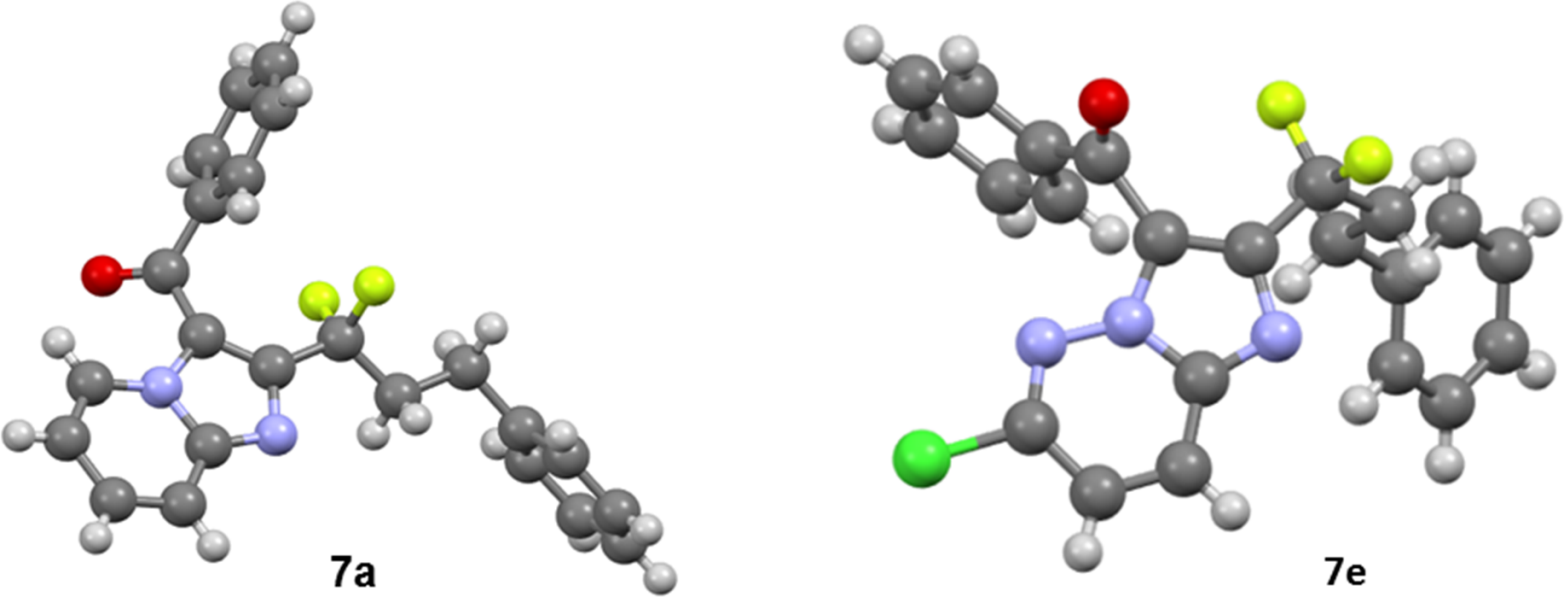
Structures of **7a** and **7e** by X-ray crystallography analysis.

The structures of molecules **7** are in full agreement with their spectroscopical (NMR) and analytical data (HRMS). For the imidazopyridines, the structure of **7a** was confirmed by X-ray analysis (Figure 2) [30] and the other derivatives were proposed by analogy. In the same way as for the imidazopyridazines, the structure of **7e** was established by X-ray analysis [30] and this was extended to the other derivatives. These results unambiguously demonstrate the regiochemistry of the reaction. These cascade reactions proceed first through a Michael addition of the primary amine on the enone, followed by an intramolecular cyclization by the pyridine/pyrimidine nucleus. Unfortunately, no crystal structure could be obtained for the imidazopyrimidines and therefore the corresponding structures **7d** and **7g** were proposed by analogy.

**Scheme 4:**
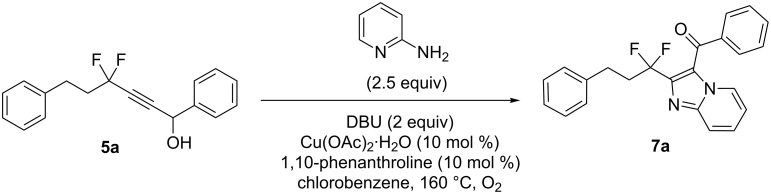
One-pot synthesis of **7a**.

These oxidative coupling conditions appeared compatible with the first isomerization step, therefore, the possibility of a "one-pot" reaction was considered. Indeed, by heating alcohol **5a** (Table 1, entry 1) with 2-aminopyridine and DBU (1,8-diazabicycloundec-7-ene) under the same conditions as mentioned above, the desired imidazopyridine derivative **7a** was isolated in 33% yield (Scheme 4). This one-pot process gives an overall yield very close to the two-step reaction (38%).

Further, the halogen-substituted substrate **7e** appeared as an attractive precursor to increase the molecular diversity around this scaffold. In order to explore this possibility, we performed two Suzuki–Miyaura reactions, as representative examples of Pd-catalyzed coupling processes (Table 3). They gave the target molecules **8** and **9** in 46% and 53% yields, respectively. On the other hand, two nucleophilic substitution reactions using phenol and morpholine gave the expected heterocycles **10** and **11** in 73% and 23% yields, respectively.

**Table 3:** Coupling and substitution reactions.

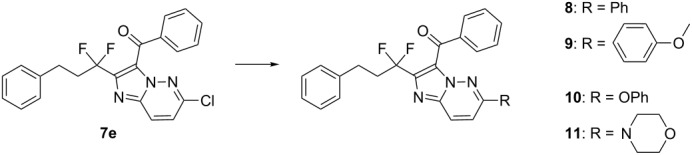				

Entry	Product	R	Conditions	Yield (%)

1	**8**	Ph	PhB(OH)_2_ Na_2_CO_3_, PdCl_2_(dppf)_2_ EtOH/H_2_O, 16 h	46
2	**9**	*p-*MeOPh	methoxyphenylboronic acid, Na_2_CO_3_, PdCl_2_(dppf)_2_ EtOH/H_2_O, 16 h	53
3	**10**	PhO	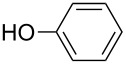 K_2_CO_3_, DMF, 120 °C, 5 h	73
4	**11**	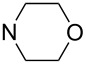	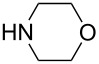 NEt_3_, EtOH, reflux, 2 days	23

## Conclusion

In summary, we developed a short and completely regioselective method for the synthesis of imidazo[1,2-*a*]-*N*-heterocycles with *gem*-fluorinated side chains starting from easily accessible propargylic fluorides. Although the yields are only moderate to fair, this short (1–2 steps) method offers significant flexibility to prepare focused libraries of molecules with this core structure. Such new fluorine-containing heteroaromatic frameworks would be of much interest for biological studies in different areas of life sciences.

## Experimental

### Representative procedure for the synthesis of imidazopyridine **7a**

The syntheses of propargylic fluorides **5** and enones **6** were performed in a similar way as described before [26].

### Synthesis of 4,4-difluoro-1,6-diphenylhex-2-yn-1-ol (**5a**)

To a solution of *gem*-difluoro intermediate **4** [28] (500 mg, 2.77 mmol, 1 equiv) in anhydrous THF (6 mL) cooled at −80 °C was added dropwise under nitrogen a 2.5 M solution of *n*-BuLi in hexanes (1.3 mL, 3.30 mmol, 1.2 equiv). The mixture was stirred for 1 h at a temperature below −80 °C before dropwise addition of the aldehyde (0.35 mL, 3.33 mmol, 1.2 equiv) in anhydrous THF (4 mL). The reaction mixture was stirred for additional 45 min at *t* < −80 °C and then allowed to warm to rt for 2 h. The mixture was then treated with a saturated ammonium chloride solution and extracted with ethyl acetate. The combined organic phases were washed with water, dried over Na_2_SO_4_ and concentrated in vacuo. The crude product was purified by chromatography on silica gel, using a mixture of petroleum ether/ethyl acetate as eluent. After purification by chromatography on silica gel, propargylic alcohol **5a** was obtained as a colorless oil (580 g, 73% yield); R*_f_* 0.46 (petroleum ether/AcOEt 8:2); ^1^H NMR (CDCl_3_, 300 MHz) δ 7.59–7.25 (m, 10H), 5.54 (t, *J_HF_* = 3.9 Hz, 1H), 2.99–2.93 (m, 2H), 2.54–2.38 (m, 2H), 2.83 (br s, 1H); ^13^C NMR (CDCl_3_, 75 MHz) δ 139.7, 138.9 (t, *^4^J* = 1.1 Hz), 128.8, 128.7 (2C), 128.5 (2C), 128.3 (2C), 126.5, 126.3, 125.9, 114.1 (t, *^1^J* = 233.4 Hz), 87.0 (t, *^3^J* = 6.8 Hz), 79.1 (t, *^2^J* = 40.9 Hz), 64.0 (t, *^4^J* = 1.8 Hz), 40.8 (t, *^2^J* = 26.1 Hz), 28.9 (t, *^3^J* = 4.0 Hz); ^19^F NMR (CDCl_3_, 282 MHz) δ −83.45 (td, *J_FH_* = 14.6, 3.9 Hz); HRMS (ESI) *m*/*z* [M + Na]^+^: calcd. for C_18_H_16_OF_2_Na, 309.10614; found, 309.1060 (0 ppm); *m*/*z* [M – HF + Na]^+^: calcd. for C_18_H_15_OFNa, 289.09991; found, 289.0992 (2 ppm).

### Synthesis of (*E*)-4,4-difluoro-1,6-diphenylhex-2-en-1-one (**6a**)

The previous difluoropropargylic alcohol **5a** (540 mg, 1.88 mmol, 1 equiv) was dissolved in THF (4 mL), then DBU (0.42 mL, 2.82 mmol, 1.5 equiv) was added and the reaction mixture was stirred at room temperature. After 2 h, ^19^F NMR showed 100% conversion and the reaction mixture was neutralized with a saturated solution of NH_4_Cl. After extraction with ethyl acetate, the organic phases were washed with water, dried (Na_2_SO_4_) and concentrated in vacuo. The crude product was purified by chromatography on silica gel, using a mixture of petroleum ether/ethyl acetate as eluent. Enone **6a** was isolated as a colorless oil (335 mg, 62% yield); R*_f_* 0.43 (petroleum ether/AcOEt 9:1); ^1^H NMR (CDCl_3_, 500 MHz) δ 8.04 (m, 2H), 7.66–7.64 (m, 1H), 7.57–7.52 (m, 2H), 7.35–7.28 (m, 6H), 6.97 (m, 1H), 2.93–2.92 (m, 2H), 2.41–2.35 (m, 2H); ^13^C NMR (CDCl_3_, 125 MHz) δ 188.93, 139.8, 138.3 (t, *^2^J* = 27.1 Hz), 136.7, 133.5, 128.7 (2C), 128.6 (2C), 128.5 (2C), 128.1 (2C), 127.6 (t, *^3^J* = 7.5 Hz), 126.3, 120.6 (t, *^1^J* = 240.4 Hz), 38.9 (t, *^2^J* = 25.9 Hz), 28.2 (t, *^3^J* = 4.3 Hz); ^19^F NMR (CDCl_3_, 282 MHz) δ −98.84 (m); HRMS (ESI) *m*/*z* [M + Na]^+^: calcd. for C_18_H_16_OF_2_Na, 309.10614; found, 309.1059 (1 ppm).

### Synthesis of (2-(1,1-difluoro-3-phenylpropyl)imidazo[1,2-*a*]pyridin-3-yl)(phenyl)methanone (**7a**)

A mixture of 2-aminopyridine (20 mg, 0.21 mmol, 1.2 equiv), enone **6a** (51 mg, 0.17 mmol, 1 equiv), Cu(OAc)_2_·H_2_O (3.6 mg, 0.02 mmol, 10 mol %), and 1,10-phenanthroline (2.5 μL, 0.02 mmol, 10 mol %) in chlorobenzene (1 mL) was stirred in a reaction tube at 160 °C under an O_2_ atmosphere. After 25 h, ^19^F NMR monitoring indicated complete consumption of the starting material. The reaction mixture was cooled to room temperature, filtered and extracted with dichloromethane. The filtrate was concentrated and the crude product was purified by column chromatography on silica gel, using petroleum ether/ethyl acetate as eluent. **7a** was isolated as white crystals (32 mg, 62% yield); R*_f_* 0.46 (petroleum ether/EtOAc 7:3); Mp: 117 °C; ^1^H NMR (CDCl_3_, 300 MHz) δ 8.76 (d, *J* = 6.9 Hz, 1H), 7.89 (s, 1H), 7.86 (s, 1H), 7.75 (d, *J* = 9.0 Hz, 1H), 7.64 (t, *J* = 7.3 Hz, 1H), 7.53–7.43 (m, 3H), 7.26–7.13 (m, 5H), 7.02 (t, *J* = 6.7 Hz, 1H), 2.72–2.65 (m, 4H); ^13^C NMR (CDCl_3_, 75 MHz) δ 187.9, 146.1, 145.1, 140.4, 139.5 (t, ^3^*J* = 2.1 Hz), 133.3 (2C), 129.5, 128.4 (2C), 128.3 (3C), 128.2 (2C), 127.3, 126.0, 120.6, 120.3 (t, ^1^*J* = 239.8 Hz), 118.2, 114.7, 39.3 (t, ^2^*J* = 25.1 Hz), 28.3 (t, ^3^*J* = 4.4 Hz); ^19^F NMR (CDCl_3_, 282 MHz) δ −90.93 (t, *J* = 15.7 Hz); HRMS (ESI) *m*/*z* [M + Na]^+^: calcd. for C_23_H_18_N_2_OF_2_Na, 399.12794; found, 399.1279 (0 ppm); *m*/*z* [M + H]^+^: calcd. for C_23_H_19_N_2_OF_2_, 377.14599; found, 377.1454 (2 ppm); *m*/*z* [M – HF + Na]^+^: calcd. for C_23_H_17_N_2_OFNa, 379.12171; found, 379.1216 (0 ppm).

### One pot synthesis of (2-(1,1-difluoro-3-phenylpropyl)imidazo[1,2-*a*]pyridin-3-yl)(phenyl)methanone (**7a**)

A mixture of 2-aminopyridine (25 mg, 0.26 mmol, 2.5 equiv), alcohol **5a** (30 mg, 0.10 mmol, 1 equiv), DBU (0.03 mL, 0.20 mmol, 2 equiv), Cu(OAc)_2_·H_2_O (2.1 mg, 0.01 mmol, 10 mol %), and 1,10-phenanthroline (1.4 μL, 0.01 mmol, 10 mol %) in chlorobenzene (1 mL) was stirred in a reaction tube at 160 °C under an O_2_ atmosphere. After 4 h monitoring by ^19^F NMR indicated the disappearance of the starting material. Thus the mixture was cooled to room temperature, filtered, washed and extracted with dichloromethane. The organic phase was concentrated and the crude product was purified by column chromatography on silica gel, using petroleum ether/ethyl acetate as eluent. Imidazopyridine **7a** was isolated in 33% yield.

## Supporting Information

File 1 Experimental details and characterization data of new compounds with copies of ^1^H, ^13^C and ^19^F NMR spectra.
