# Supplementary material for: Preparation of imidazo[1,2-a]-N-heterocyclic derivatives with gem-difluorinated side chains
Source: Beilstein J Org Chem. 2017 Oct 10;13:2115–21. doi: 10.3762/bjoc.13.208 (PMC5647726; doi:10.3762/bjoc.13.208)
Supplement: File 1 — Experimental details and characterization data of new compounds with copies of 1H, 13C and 19F NMR spectra. [file Beilstein_J_Org_Chem-13-2115-s001.pdf]

Supporting Information

for

Preparation of imidazo[1,2-*a*]-*N*-heterocyclic derivatives with *gem*-difluorinated side chains

Layal Hariss[1], Kamal Bou Hadir[2], Mirvat El-Masri[1], Thierry Roisnel[3], René Grée[*3] and Ali Hachem[*1]

Address: [1]Laboratory for Medicinal Chemistry and Natural Products, Lebanese University, Faculty of Sciences (1) and PRASE-EDST, Hadath, Beirut, Lebanon, [2]American University of Beirut, Department of Chemistry, Beirut 11-0236, Lebanon and [3]Université de Rennes 1, Institut des Sciences Chimiques de Rennes, CNRS UMR 6226, Avenue du Général Leclerc, 35042 Rennes Cedex, France

Email: René Gree - rene.gree@univ-rennes1.fr; Ali Hachem - ahachem@ul.edu.lb

*Corresponding author

Experimental details and characterization data of new compounds with copies of ^{1}H, ^{13}C and ^{19}F NMR spectra

Contents

1. General methods……… S4

2. General procedure for the synthesis of difluoropropargylic alcohols (**5**) S5

2.1. Synthesis of 1-(2-bromophenyl)-4,4-difluoro-6-phenylhex-2-yn-1-ol (**5b**)………… S5

2.2. Synthesis of 4,4-difluoro-1-(naphthalen-2-yl)-6-phenylhex-2-yn-1-ol (**5c**)………… S6

2.3. Synthesis of 4,4-difluoro-1-(4-methoxyphenyl)-6-phenylhex-2-yn-1-ol (**5d**)……… S6

2.4. Synthesis of 7-(benzyloxy)-4,4-difluoro-1-phenylhept-2-yn-1-ol (**5e**)………………. S7

3. General procedure for the synthesis of *gem*-difluoroenones (**6**) S8

3.1. Synthesis of (E)-1-(2-bromophenyl)-4,4-difluoro-6-phenylhex-2-en-1-one (**6b**)… S8

3.2. Synthesis of (E)-4,4-difluoro-1-(naphthalen-2-yl)-6-phenylhex-2-en-1-one(**6c**)… S9

3.3. Synthesis of (E)-4,4-difluoro-1-(4-methoxyphenyl)-6-phenylhex-2-en-1-one (**6d**) S9

3.4. Synthesis of (E)-7-(benzyloxy)-4,4-difluoro-1-phenylhept-2-en-1-one (**6e**)……… S10

4. General procedure for the oxidative coupling S10

4.1. Synthesis of (2-(1,1-difluoro-3-phenylpropyl)-7-methyl*H*-imidazo[1,2-*a*]pyridin-3-yl)(phenyl)methanone (**7b**)…………. S11

4.2. Synthesis of (6-bromo-2-(1,1-difluoro-3-phenylpropyl)H-imidazo[1,2-a]pyridin-3-yl)(phenyl)methanone (**7c**)…………. S11

4.3. Synthesis of (2-(1,1-difluoro-3-phenylpropyl)imidazo[1,2-a]pyrimidin-3-yl)(phenyl)methanone (**7d**)…………. S12

4.4. Synthesis of (6-chloro-2-(1,1-difluoro-3-phenylpropyl)*H*-imidazo[1,2-*a*]pyridin-3-yl)(phenyl)methanone(**7e**)…………. S13

4.5. Synthesis of (2-(4-(benzyloxy)-1,1-difluorobutyl)H-imidazo[1,2-a]pyridin-3-yl)(phenyl)methanone (**7f**)…………. S13

4.6. Synthesis of (2-(4-(benzyloxy)-1,1-difluorobutyl)imidazo[1,2-a]pyrimidin-3-yl)(phenyl)methanone (**7g**)…………. S14

4.7. Synthesis of (2-bromophenyl)(2-(1,1-difluoro-3-phenylpropyl)*H*-imidazo[1,2-*a*]pyridin-3-yl)methanone (**7h**)…………. S14

4.8. Synthesis of (2-(1,1-difluoro-3-phenylpropyl)H-imidazo[1,2-a]pyridin-3-yl)(naphthalen-2-yl)methanone (**7i**)…………. S15

4.9. Synthesis of (2-(1,1-difluoro-3-phenylpropyl)H-imidazo[1,2-a]pyridin-3-yl)(4-methoxyphenyl)methanone (**7j**)…………. S16

5. General procedure of Suzuki-Miyaura coupling S16

5.1. Synthesis of (2-(1,1-difluoro-3-phenylpropyl)-6-phenylimidazo[1,2-b]pyridazin-3-yl)(phenyl)methanone (**8**)…………. S17

5.2. Synthesis of [2-(1,1-Difluoro-3-phenyl-propyl)-6-(4-methoxy-phenyl)-imidazo[1,2-b]pyridazin-3-yl]-phenyl-methanone (**9**)…………. S17

6. General procedure for Nucleophilic substitution of phenol S18

6.1. Synthesis of (2-(1,1-difluoro-3-phenylpropyl)-6-phenoxyimidazo[1,2-b]pyridazin-3-yl)(phenyl)methanone (**10**)……………………………………………………………………………….…… S18

7. General procedure for Nucleophilic substitution of morpholine S19

7.1. Synthesis of (2-(1,1-difluoro-3-phenylpropyl)-6-morpholinoimidazo[1,2-b]pyridazin-3-yl)(phenyl)methanone (**11**)……………………………………………………………………………. S19

^{1}H, ^{13}C and ^{19}F NMR spectra for all new compounds… …………S20–S67

1. General methods

The solvents used were freshly distilled under anhydrous conditions, unless otherwise specified. Some reactions were carried out under a nitrogen atmosphere and dry conditions. The reaction mixtures were magnetically stirred with Teflon stirring bars, and the temperatures were measured externally. Reactions that required anhydrous conditions were carried out by using oven dried (120 °C, 24 h) glassware. Yields refer to chromatographically and spectroscopically (^{1}H, ^{13}C, and ^{19}F NMR) homogeneous materials. Reactions were monitored by ^{19}F NMR and by thin-layer chromatography (TLC), carried out on 0.25 mm Merck silica gel plates (60 F254) with detection by UV light or staining with *p*-anisaldehyde. The eluents used were mixtures of petroleum ether and ethyl acetate (EtOAc). Sigma Aldrich silica gel (60 Å, particle size 0.040–0.063 mm) was used for column chromatography. Nuclear magnetic resonance (NMR) spectra have been recorded with Bruker Avance 500, and 300 spectrometers. ^{1}H NMR spectra: δ (H) are given in ppm relative to tetramethylsilane (TMS), using [δ ($CHCl_3$) = 7.26 ppm] as internal reference. ^{13}C NMR spectra: δ (C) are given in ppm relative to TMS, using [*δ* ($CDCl_3$) = 77.0 ppm] as internal reference. ^{19}F NMR spectra: δ (F) are given in ppm relative to $CFCl_3$ = 0.0 ppm as external reference. Multiplicities were designated as singlet (s), doublet (d), triplet (t), multiplet (m) or br (broad). Mass spectral analyses have been performed at the Centre Régional de Mesures Physiques de l'Ouest (CRMPO) in Rennes (France).

2. General procedure for the synthesis of difluoropropargylic alcohols (5)

The preparation of propargylic fluorides **5** and enones **6** was performed in a similar way as described before [26].

To a solution of *gem*-difluoro intermediate **4** (1 equiv) in anhydrous THF (2 mL per mmol) cooled at −80°C was added dropwise under nitrogen a 2.5 M solution of *n*-BuLi in hexanes (1.2 equiv). The mixture was stirred for 1 h at −80 °C before dropwise addition of the aldehyde (1.2 equiv) in anhydrous THF (1 mL per mmol). The reaction mixture was stirred for additional 45 min at −80 °C and then allowed to warm to rt for 2 h. The mixture was then treated with a saturated ammonium chloride solution and extracted with ethyl acetate. The combined organic phases were washed with water, dried over Na_2SO_4 and concentrated in vacuo. The crude is purified by chromatography on silica gel, using a mixture of petroleum ether/ethyl acetate as eluent.

2.1. Synthesis of 1-(2-bromophenyl)-4,4-difluoro-6-phenylhex-2-yn-1-ol (5b)

The reaction was performed with **4** (305 mg, 1.69 mmol) and 2-bromobenzaldehyde (0.24 mL, 2.03 mmol), according to the general procedure. After purification by chromatography on silica gel, the propargylic alcohol was obtained as a white solid (430 mg, 70% yield); $\mathbf{R_f}$ 0.54 (petroleum ether / AcOEt: 8/2); Mp: 84°C. **^{1}H NMR ($CDCl_3$, 300 MHz):** δ, ppm: 7.62 (dd, 1H, J = 7.7, 1.6 Hz), 7.51 (dd, 1H, J = 7.9, 1.1 Hz), 7.31 (dt, 1H, J = 7.5, 1.1 Hz), 7.17-7.26 (m, 2H), 7.16 (m, 1H), 7.08-7.15 (m, 3H), 5.77 (t, 1H, J_{HF} = 4.0 Hz), 2.75-2.83 (m, 2H), 2.20-2.40 (m, 2H), 2.14 (br s, 1H). **^{13}C NMR ($CDCl_3$, 75 MHz):** δ, ppm: 139.7, 137.9, 133.2, 130.4, 128.6 (2C), 128.4 (2C), 128.0 (2C), 126.4, 122.5, 114.1 (t, ^1J = 233.6 Hz), 85.9 (t, ^3J = 6.8 Hz), 79.2 (t, ^2J =

41.0 Hz), 63.7 (t, ^{4}J = 1.8 Hz), 40.9 (t, ^{2}J = 26.1 Hz), 29.0 (t, ^{3}J = 4.1 Hz). **^{19}F NMR ($CDCl_3$, 282 MHz):** δ, ppm: -83.89 (td, J_{FH} = 14.5, 4.0 Hz). HRMS (ESI): calcd. for $C_{18}H_{15}OF_2{}^{79}BrNa$: m/z $[M+Na]^+$ 387.01665; found: 387.0166 (0 ppm).

2.2. Synthesis of 4,4-difluoro-1-(naphthalen-2-yl)-6-phenylhex-2-yn-1-ol (5c)

The reaction was performed with **4** (400 mg, 2.22 mmol) and 2-naphtaldehyde (0.36 mL, 2.66 mmol), according to the general procedure. After purification by chromatography on silica gel, propargylic alcohol was obtained as a yellow oil (530 mg, 71% yield); **R_f** 0.54 (petroleum ether / AcOEt: 8/2). **^{1}H NMR ($CDCl_3$, 300 MHz):** δ, ppm: 7.86 (s, 1H), 7.71-7.82 (m, 3H), 7.50-7.53 (dd, 1H, J = 8.5, 1.8 Hz), 7.37-7.46 (m, 2H), 7.06-7.24 (m, 5H), 5.59 (t, 1H, J_{HF} = 4.0 Hz), 2.76-2.86 (m, 2H), 2.23-2.42 (m, 2H). **^{13}C NMR ($CDCl_3$, 75 MHz):** δ, ppm: 139.8, 136.4, 133.2, 128.9, 128.6 (2C), 128.3 (2C), 128.2 (2C), 127.7, 126.6, 126.5, 126.4, 125.6, 124.1, 114.2 (t, ^{1}J = 233.7 Hz), 87.1 (t, ^{3}J = 6.6 Hz), 79.7 (t, ^{2}J = 41.1 Hz), 64.4, 40.9 (t, ^{2}J = 26.3 Hz), 29.0 (t, ^{3}J = 4.0 Hz). **^{19}F NMR ($CDCl_3$, 282 MHz):** δ, ppm: -83.63 (td, J_{FH} = 14.5, 4.0 Hz). HRMS (ESI): calcd. for $C_{22}H_{18}OF_2Na$: m/z $[M+Na]^+$ 359.12179; found: 359.1218 (0 ppm); calcd. for $C_{22}H_{17}OFNa$: m/z $[M-HF+Na]^+$ 339.11556; found: 339.1153 (1 ppm).

2.3. Synthesis of 4,4-difluoro-1-(4-methoxyphenyl)-6-phenylhex-2-yn-1-ol (5d)

The reaction was performed with **4** (295 mg, 1.64 mmol) and *p*-anisaldehyde (0.06 mL, 1.96 mmol), according to the general procedure. After purification by chromatography on silica gel, propargylic alcohol was obtained as a yellow oil (140 mg, 27% yield); **R_f** 0.62 (petroleum ether / AcOEt: 8/2). **^{1}H NMR**

(CDCl$_3$, 300 MHz): δ, ppm: 7.47 (m, 1H), 7.45 (m, 1H), 7.24-7.37 (m, 3H), 7.23 (m, 1H), 7.21 (m, 1H), 6.96 (m, 1H), 6.93 (m, 1H), 5.49 (m, 1H), 3.85 (s, 3H), 2.86-2.96 (m, 2H), 2.32-2.47 (m, 2H), 2.21 (d, 1H, *J* = 6.3 Hz). **^{13}C NMR (CDCl$_3$, 75 MHz):** δ, ppm: 160.1, 139.8, 131.3, 128.6 (2C), 128.4 (2C), 128.1 (2C), 126.4, 117.3, 114.2 (t, ^1J = 233.4 Hz), 114.1 (2C), 87.2 (t, ^3J = 6.8 Hz), 79.2 (t, ^2J = 40.9 Hz), 63.9 (t, ^4J = 1.9 Hz), 40.9 (t, ^2J = 26.3 Hz), 29.0 (t, ^3J = 4.0 Hz). **^{19}F NMR (CDCl$_3$, 282 MHz):** δ, ppm: -83.64 (td, *J* = 14.5, 4.0 Hz). HRMS (ESI): calcd. for $C_{19}H_{18}O_2F_2Na$: m/z $[M+Na]^+$ 339.11671; found: 339.1165 (1 ppm); calcd. for $C_{19}H_{17}O_2FNa$: m/z $[M-HF+Na]^+$ 319.11048; found: 319.1106 (0 ppm).

2.4. Synthesis of 7-(benzyloxy)-4,4-difluoro-1-phenylhept-2-yn-1-ol (5e)

The reaction was performed with **4** (250 mg, 1.12 mmol) and benzaldehyde (0.14 mL, 1.34 mmol), according to the general procedure. After purification by chromatography on silica gel, propargylic alcohol was obtained as a yellow oil (240 mg, 65% yield); **R_f** 0.20 (petroleum ether / AcOEt: 8/2). **^{1}H NMR (CDCl$_3$, 300 MHz):** δ, ppm: 7.37-7.40 (m, 2H), 7.14-7.35 (m, 8H), 5.38 (d, 1H, J_{HF} = 3.9 Hz), 4.40 (s, 2H), 3.43 (t, 2H, *J* = 6.2 Hz), 2.71(d, 1H, *J* = 6.2 Hz), 2.10 (m, 2H), 1.79 (m, 2H). **^{13}C NMR (CDCl$_3$, 75 MHz):** δ, ppm: 138.9, 138.1, 128.8 (2C), 128.4 (2C), 127.6 (2C), 127.6 (2C), 126.6 (2C), 114.7 (t, ^1J = 232.8 Hz), 86.7 (t, ^3J = 6.7 Hz), 79.2 (t, ^2J = 41.1 Hz), 72.8, 68.8, 64.0, 36.1 (t, ^2J = 26.4 Hz), 23.2 (t, ^3J = 3.5 Hz). **^{19}F NMR (CDCl$_3$, 282 MHz):** δ, ppm: -83.22 (td, *J* = 15.0, 3.9 Hz). HRMS (ESI): calcd. for $C_{20}H_{20}O_2F_2Na$: m/z $[M+Na]^+$ 353.13236; found: 353.1325 (0 ppm); calcd. for $C_{20}H_{19}O_2FNa$: m/z $[M-HF+Na]^+$ 333.12613; found: 333.1262 (0 ppm).

3. General procedure for the synthesis of *gem*-difluoroenones (6)

The difluoropropargylic alcohol **5** (1 equiv) was dissolved in THF (2 mL per mmol), then DBU (1.5 equiv) was added, and stirred at room temperature for the appropriate time (monitored by ^{19}F NMR). After completion of the reaction, the mixture was neutralized with saturated solution of NH_4Cl. After extraction with ethyl acetate, the organic phases were washed with water, dried (Na_2SO_4) and concentrated in vacuo. The crude product was purified by chromatography on silica gel, using a mixture of petroleum ether/ethyl acetate as eluent.

3.1. Synthesis of (*E*)-1-(2-bromophenyl)-4,4-difluoro-6-phenylhex-2-en-1-one (6b)

The reaction was performed with **5b** (400 mg, 1.16 mmol) according to the general procedure. After 4 h, ^{19}F NMR showed 100% conversion. After purification by flash chromatography on silica gel, the enone **6b** was isolated as a yellow solid (240 mg, 60% yield); **R***f* 0.48 (petroleum ether / AcOEt: 8/2); Mp: 87°C. **^{1}H NMR ($CDCl_3$, 300 MHz):** δ, ppm: 7.56 (dt, 1H, *J* = 7.6, 0.8 Hz), 7.07-7.36 (m, 8H), 6.87 (dt, 1H, *J* = 15.9, 2.1 Hz), 6.52 (dt, 1H, *J* = 27.08, 11.2 Hz), 2.75 (m, 2H), 2.22 (m, 2H). **^{13}C NMR ($CDCl_3$, 75 MHz):** δ, ppm: 193.0, 139.8, 139.5 (t, ^2J = 27.7 Hz), 133.6, 132.2, 131.3 (t, ^3J = 7.8 Hz), 129.5, 128.6 (3C), 128.2 (2C), 127.5, 126.4, 120.4 (t, ^1J = 240.7 Hz), 119.5, 38.9 (t, ^2J = 25.9 Hz), 28.3 (t, ^3J = 4.6 Hz). **^{19}F NMR ($CDCl_3$, 282 MHz):** δ, ppm: -99.07 (tdd, *J* = 15.9, 11.2, 2.1 Hz). HRMS (ESI): calcd. for $C_{18}H_{15}OF_2{}^{79}BrNa$: m/z $[M+Na]^+$ 387.01665; found: 387.0163 (1 ppm).

3.2. Synthesis of (*E*)-4,4-difluoro-1-(naphthalen-2-yl)-6-phenylhex-2-en-1-one (6c)

The reaction was performed with **5c** (550 mg, 1.60 mmol) according to the general procedure. After 2 h, ^{19}F NMR showed 100% conversion. After purification by flash chromatography on silica gel, the enone 6c was isolated as a white solid (275 mg, 50% yield); **R_f** 0.69 (petroleum ether / AcOEt: 9/1); Mp: 66°C. **^{1}H NMR ($CDCl_3$, 300 MHz):** δ, ppm: 8.35 (s,1H), 7.93 (dd, 1H, *J* = 8.6, 1.7 Hz), 7.85 (m, 1H), 7.73-7.82 (m, 2H), 7.36-7.52 (m, 3H), 7.15-7.24 (m, 2H), 7.06-7.14 (m, 3H), 6.90 (dt, 1H, *J* = 15.5, 11.3 Hz), 2.73-2.80 (m, 2H), 2.14-2.33 (m, 2H). **^{13}C NMR ($CDCl_3$, 75 MHz):** δ, ppm: 188.7, 139.9, 138.3 (t, ^2J = 27.0 Hz), 135.7, 134.1, 132.4, 130.7, 129.6, 128.9, 128.8, 128.6 (2C), 128.2 (2C), 127.8, 127.6 (t, ^3J = 7.6 Hz), 127.0, 126.3, 124.0, 120.7 (t, ^1J = 240.4 Hz), 39.0 (t, ^2J = 26.0 Hz), 28.3 (t, ^3J = 4.5 Hz). **^{19}F NMR ($CDCl_3$, 282 MHz):** δ, ppm: -98.70 (tdd, *J* = 15.9, 11.7, 2.1 Hz). HRMS (ESI): calcd. for $C_{22}H_{18}OF_2Na$: m/z $[M+Na]^+$ 359.12179; found: 359.1214 (1 ppm).

3.3. Synthesis of (*E*)-4,4-difluoro-1-(4-methoxyphenyl)-6-phenylhex-2-en-1-one (6d)

The reaction was performed with **5d** (120 mg, 0.43 mmol) according to the general procedure. After 8 h, ^{19}F NMR showed 100% conversion. After purification by flash chromatography on silica gel, the enone **6d** was isolated as yellow oil (25 mg, 21% yield); **R_f** 0.63 (petroleum ether / AcOEt: 8/2). **^{1}H NMR ($CDCl_3$, 300 MHz):** δ, ppm: 7.94-7.99 (m, 2H), 7.30-7.38 (m, 1H), 7.25-7.30 (m, 2H), 7.16-7.22 (m, 3H), 6.93-6.99 (m, 2H), 6.85 (dt, 1H, *J* = 15.5, 11.7 Hz), 3.87 (s, 3H), 2.79-2.86 (m, 2H), 2.20-2.40 (m, 2H). **^{13}C NMR ($CDCl_3$, 75 MHz):** δ, ppm: 187.3, 164.0, 140.0, 137.6 (t, ^2J = 27.0 Hz), 131.1 (2C),

129.8, 128.6 (2C), 128.2 (2C), 127.6 (t, ^3J = 7.5 Hz), 126.3, 120.7 (t, ^1J = 240.2 Hz), 114.0 (2C), 55.5, 39.1 (t, ^2J = 26.0 Hz), 28.3 (t, ^3J = 4.5 Hz). **^{19}F NMR ($CDCl_3$, 282 MHz):** δ, ppm: -98.74 (m). HRMS (ESI): calcd. for $C_{19}H_{18}O_2F_2Na$: m/z $[M+Na]^+$ 339.11671; found: 339.1166 (0 ppm); calcd. for $C_{19}H_{17}O_2FNa$: m/z $[M-HF+Na]^+$ 319.11048; found: 319.1104 (0 ppm).

3.4. Synthesis of (*E*)-7-(benzyloxy)-4,4-difluoro-1-phenylhept-2-en-1-one (6e)

The reaction was performed with **5e** (150 mg, 0.90 mmol) according to the general procedure. After 2.5 h, ^{19}F NMR showed 100% conversion. After purification by flash chromatography on silica gel, the enone **6e** was isolated as yellow oil (100 mg, 66% yield); **R_f** 0.77 (petroleum ether / AcOEt: 8/2). **^{1}H NMR ($CDCl_3$, 300 MHz):** δ, ppm: 7.91 (m, 1H), 7.88 (m, 1H), 7.53 (m, 1H), 7.43 (m, 2H), 7.18-7.31 (m, 6H), 6.79 (m, 1H), 4.44 (s, 2H), 3.46 (t, 2H, *J* = 6.1 Hz), 2.07 (m, 2H), 1.76 (m, 2H). **^{13}C NMR ($CDCl_3$, 75 MHz):** δ, ppm: 189.2, 138.6 (t, ^2J = 27.3 Hz), 138.3, 136.9, 133.6, 128.8 (2C), 128.7 (2C), 128.4 (2C), 127.6 (t, ^3J = 6.2 Hz), 127.6 (4C), 121.1 (t, ^1J = 239.7 Hz), 69.1, 34.2 (t, ^2J = 26.3 Hz), 22.7 (t, ^3J = 4.1 Hz). **^{19}F NMR ($CDCl_3$, 282 MHz):** δ, ppm: -98.58 (m). HRMS (ESI): calcd. for HRMS (ESI): calcd. for $C_{20}H_{20}O_2F_2Na$: m/z $[M+Na]^+$ 353.13236; found: 353.1320 (1 ppm); calcd. for $C_{20}H_{19}O_2FNa$: m/z $[M-HF+Na]^+$ 333.12613; found: 333.1257 (1 ppm)

4. General procedure for the oxidative coupling

A mixture of 2-aminopyridine (1.2 equiv), enone **6** (1 equiv), $Cu(OAc)_2·H_2O$ (10 mol %), and 1,10-phenantroline (10 mol %) in chlorobenzene (1 mL per 0.17 mmol) was stirred in a reaction tube at 160 °C under O_2 atmosphere. The reaction was monitored by ^{19}F NMR. After completion of the reaction, it was

cooled to room temperature, then it was filtered and washed with dichloromethane. The filtrate was concentrated and the crude product was purified by column chromatography on silica gel, using petroleum/ethyl acetate as eluent.

4.1. Synthesis of (2-(1,1-difluoro-3-phenylpropyl)-7-methylimidazo[1,2-*a*]pyridin-3-yl)(phenyl)methanone (7b)

The reaction was performed with **6a** (50 mg, 0.17 mmol) according to the general procedure. After 20 h, ^{19}F NMR monitoring showed the absence of starting material. After purification by flash chromatography on silica gel, **7b** was isolated as white crystals (30 mg, 60% yield); **R_f** 0.22 (petroleum ether-EtOAc; 8.5:1.5); Mp: 105°C. **^1H NMR ($CDCl_3$, 300 MHz):** δ, ppm: 8.68 (d, 1H, J = 7.1 Hz), 7.86 (s, 1H), 7.84 (s, 1H), 7.58-7.65 (m, 1H), 7.45-7.52 (m, 3H), 7.20-7.25 (m, 2H), 7.11-7.18 (m, 3H), 6.85 (dd, 1H, J = 7.15, 1.40 Hz), 2.58-2.70 (m, 4H), 2.47 (s, 3H). **^{13}C NMR ($CDCl_3$, 75 MHz):** δ, ppm: 187.8, 146.6, 145.3 (t, ^2J = 32.2 Hz), 140.5, 139.9, 139.8 (t, ^3J = 2.3 Hz), 133.1, 129.4 (t, ^5J = 1.4 Hz), 128.4 (3C), 128.3 (2C), 128.2 (2C), 126.5, 126.0, 120.3, 120.3 (t, ^1J = 239.6 Hz), 117.4, 116.6, 39.3 (t, ^2J = 25.2 Hz), 28.3 (t, ^3J = 4.5 Hz), 21.4. **^{19}F NMR ($CDCl_3$, 282 MHz):** δ, ppm: -90.84 (t, J = 16.0 Hz). HRMS (ESI): calcd. for $C_{24}H_{20}N_2OF_2Na$: m/z $[M+Na]^+$ 413.14359, found: 413.1432 (1 ppm), calcd. for $C_{24}H_{19}N_2OFNa$: m/z $[M-HF+Na]^+$ 393.13736, found: 393.1369 (1 ppm),

4.2. Synthesis of (6-bromo-2-(1,1-difluoro-3-phenylpropyl)imidazo[1,2-a]pyridin-3-yl)(phenyl)methanone (7c)

The reaction was performed with **6a** (50 mg, 0.17 mmol) according to the general procedure. After 46 h, ^{19}F NMR monitoring showed the absence of starting material. After purification by flash chromatography on silica gel, **7c**

was isolated as a yellow oil (30 mg, 60% yield); **R_f** 0.57 (petroleum ether-EtOAc; 8:2). **^{1}H NMR ($CDCl_3$, 500 MHz):** δ, ppm: 8.85 (m, 1H), 7.76-7.81 (m, 2H), 7.53-7.59 (m, 2H), 7.40-7.45 (m, 3H), 7.13-7.16 (m, 2H), 7.03-7.09 (m, 3H), 2.51-2.64 (m, 4H). **^{13}C NMR ($CDCl_3$, 125 MHz):** δ, ppm: 187.7, 148.7, 145.1 (t, ^2J = 32.3 Hz), 140.2, 139.1, 133.6, 131.9, 129.5, 128.3 (4C), 128.2 (2C), 127.3, 126.0, 120.7, 120.0 (t, ^1J = 239.8 Hz), 118.6, 115.3, 109.7, 39.1 (t, ^2J = 25.1 Hz), 28.2 (t, ^3J = 4.4 Hz). **^{19}F NMR ($CDCl_3$, 470 MHz):** δ, ppm: -90.89 (t, J = 16.0 Hz). HRMS (ESI): calcd. for $C_{23}H_{17}N_2OF_2{}^{79}BrNa$: m/z $[M+Na]^+$ 477.03845; found: 477.0389 (1 ppm); calcd. for $C_{23}H_{16}N_2OF^{79}BrNa$: m/z $[M-HF+Na]^+$ 457.03222; found: 457.0341 (4 ppm).

4.3. Synthesis of (2-(1,1-difluoro-3-phenylpropyl)imidazo[1,2-*a*]pyrimidin-3-yl)(phenyl)methanone (7d)

The reaction was performed with **6a** (35 mg, 0.12 mmol) according to the general procedure. After 30 h, ^{19}F NMR monitoring showed the absence of starting material. After purification by flash chromatography on silica gel, **7d** was isolated as a colorless oil (20 mg, 57% yield); ***R_f*** 0.45 (petroleum ether-EtOAc; 7:3). **^{1}H NMR ($CDCl_3$, 500 MHz):** δ, ppm: 9.07 (dt, 1H, J = 6.9,1.6 Hz), 8.73 (s, 1H), 7.83 (m, 1H), 7.82 (m, 1H), 7.59 (m, 1H), 7.45 (m, 2H), 7.19 (m, 1H), 7.16 (m, 1H), 7.15 (m, 1H), 7.06-7.09 (m, 3H), 2.62-2.73 (m, 4H). **^{13}C NMR ($CDCl_3$, 75 MHz):** δ, ppm: 187.6, 153.6, 148.7, 146.1 (t, ^2J = 33.1 Hz), 140.3, 139.0 (t, ^3J = 2.3 Hz), 135.5, 133.7 (2C), 129.6 (t, 2C, ^4J = 1.4 Hz), 128.4 (4C), 126.1 (2C), 120.2 (t, ^1J = 239.9 Hz), 119.0, 111.1, 38.8 (t, ^2J = 24.5 Hz), 28.3 (t, ^3J = 4.5 Hz). **^{19}F NMR ($CDCl_3$, 470 MHz):** δ, ppm: -90.67 (t, J = 15.8 Hz). HRMS (ESI): calcd. for $C_{22}H_{17}N_3OF_2Na$: m/z $[M+Na]^+$ 400.12319; found: 400.1230 (0 ppm).

4.4. Synthesis of (6-chloro-2-(1,1-difluoro-3-phenylpropyl)imidazo[1,2-*a*]pyridin-3-yl)(phenyl)methanone (7e)

The reaction was performed with **6a** (260 mg, 0.90 mmol) according to the general procedure. After 24 h, ^{19}F NMR monitoring showed the absence of starting material. After purification by flash chromatography on silica gel, **7e** was isolated as yellow oil (138 mg, 53% yield); **R*f*** 0.20 (petroleum ether-EtOAc; 8:2).**^{1}H NMR (CDCl$_3$, 300 MHz):** δ, ppm: 7.90 (d, 1H, *J* = 9.5 Hz), 7.80 (m, 2H), 7.6 (tt, 1H, *J* = 6.8, 1.3 Hz), 7.41 (m, 2H), 7.07-7.21 (m, 6H), 2.57-2.78 (m, 4H). **^{13}C NMR (CDCl$_3$, 75 MHz):** δ, ppm: 185.9, 148.5, 142.0 (t, ^{2}J = 32.8 Hz), 140.2, 137.1, 136.9, 134.5, 129.9 (2C), 128.7 (2C), 128.5 (2C), 128.3 (2C), 127.6, 126.2, 124.7 (t, ^{4}J = 2.0 Hz), 121.7, 120.2 (t, ^{1}J = 240.5 Hz), 39.0 (t, ^{2}J = 25.2 Hz), 28.3 (t, ^{3}J = 4.4 Hz). **^{19}F NMR (CDCl$_3$, 282 MHz):** δ, ppm: -93.14 (t, *J* = 16.2 Hz). HRMS (ESI): calcd. for $C_{22}H_{16}N_3OF_2{}^{35}ClNa$: m/z [M+Na]$^+$ 434.08422, found: 434.0843 (0 ppm), calcd. for $C_{22}H_{15}N_3OF^{35}ClNa$ m/z [M-HF+Na]$^+$ 414.07909, found: 414.0784 (1 ppm).

4.5. Synthesis of (2-(4-(benzyloxy)-1,1-difluorobutyl)imidazo[1,2-*a*]pyridin-3-yl)(phenyl)methanone (7f)

The reaction was performed with **6e** (30 mg, 0.09 mmol) according to the general procedure. After 33 h, ^{19}F NMR monitoring showed the absence of starting material. After purification by flash chromatography on silica gel, **7f** was isolated as white crystals (17 mg, 55% yield); **R*f*** 0.11 (petroleum ether-EtOAc; 8:2); Mp: 61°C. **^{1}H NMR (CDCl$_3$, 300 MHz):** δ, ppm: 8.69 (dt, 1H, *J* = 10.0, 7.0 Hz), 7.81 (m, 1H), 7.79 (m, 1H), 7.68-7.71 (dt, 1H, *J* = 9.1, 1.0 Hz), 7.56 (m, 1H), 7.36-7.46 (m, 3H), 7.23-7.27 (m, 5H), 6.93-6.99 (dt, 1H, *J* = 7.0, 1.0 Hz), 4.41 (s, 2H), 3.42 (t, 2H, *J* = 6.4 Hz), 2.33-2.52 (m, 2H), 1.62-1.74 (m, 2H). **^{13}C NMR (CDCl$_3$, 75 MHz):** δ, ppm: 188.0, 146.1 (t, ^{2}J = 32.4 Hz), 145.2

(t, ^2J = 32.4 Hz), 139.5 (t, ^3J = 2.2 Hz), 138.4, 133.3 (2C), 129.5 (2C), 128.3 (4C), 127.6 (2C), 127.5, 127.3, 120.8 (t, ^1J = 239.5 Hz), 120.6, 118.2, 114.7, 72.7, 69.4, 34.4 (t, ^2J = 25.3 Hz), 22.5 (t, ^3J = 3.9 Hz). **^{19}F NMR ($CDCl_3$, 282 MHz):** δ, ppm: -90.39 (t, *J* = 17.3 Hz). HRMS (ESI): calcd. for $C_{25}H_{22}N_2O_2F_2Na$: m/z $[M+Na]^+$ 443.15415, found: 443.1545 (1 ppm).

4.6. Synthesis of (2-(4-(benzyloxy)-1,1-difluorobutyl)imidazo[1,2-*a*]pyrimidin-3-yl)(phenyl)methanone (7g)

The reaction was performed with **6e** (20 mg, 0.06 mmol) according to the general procedure. After 29 h, ^{19}F NMR monitoring showed the absence of starting material. After purification by flash chromatography on silica gel, **7g** was isolated as yellow oil (9 mg, 36% yield); **R_f** 0.15 (petroleum ether-EtOAc; 6:4). **^{1}H NMR ($CDCl_3$, 500 MHz):** δ, ppm: 9.11 (dd, 1H, *J* = 7.0, 2.0 Hz), 8.78 (dd, 1H, *J* = 4.0, 2.0 Hz), 7.86 (m, 2H), 7.63 (m, 1H), 7.49 (m, 2H), 7.27-7.33 (m, 5H), 7.12 (d, 1H, *J* = 7.0, 4.0 Hz), 4.45 (s, 2H), 3.47 (t, 2H, *J* = 6.4 Hz), 2.53 (m, 2H), 1.71 (m, 2H). **^{13}C NMR ($CDCl_3$, 125 MHz):** δ, ppm: 187.6, 153.5, 148.7, 146.2 (t, ^2J = 33.1 Hz), 138.9, 138.3, 135.4, 133.6, 129.5 (2C), 128.3 (4C), 127.5 (2C), 127.5, 120.6 (t, ^1J = 239.5 Hz), 118.9, 111.0, 72.7, 69.3, 33.9 (t, ^2J = 24.8 Hz), 22.4 (t, ^3J = 4.0 Hz). **^{19}F NMR ($CDCl_3$, 470 MHz):** δ, ppm: -90.33 (t, *J* = 17.3 Hz). HRMS (ESI): calcd. for $C_{24}H_{21}N_3O_2F_2Na$: m/z $[M+Na]^+$ 444.1494; found: 444.1493 (0 ppm).

4.7. Synthesis of (2-bromophenyl)(2-(1,1-difluoro-3-phenylpropyl)imidazo[1,2-*a*]pyridin-3-yl)methanone (7h)

The reaction was performed with **6b** (20 mg, 0.05 mmol) according to the general procedure. After 4 h, ^{19}F NMR monitoring showed the absence of starting material. After purification by flash chromatography on silica gel, **7h**

was isolated as a yellow oil (13 mg, 65% yield); **R_f** 0.30 (petroleum ether-EtOAc; 8:2). **^{1}H NMR ($CDCl_3$, 300 MHz):** δ, ppm: 7.73 (dt, 1H, *J* = 9.0, 1.0 Hz), 7.55 (m, 1H), 7.49 (m, 1H), 7.34-7.42 (m, 1H), 7.27-7.34 (m, 2H), 7.14-7.21 (m, 3H), 7.04-7.12 (m, 4H), 2.60-2.68 (m, 2H), 2.37-2.55 (m, 2H). **^{13}C NMR ($CDCl_3$, 75 MHz):** δ, ppm: 186.6, 147.7 (t, ^2J = 32.4 Hz), 146.4, 141.5, 140.5, 132.9, 131.7, 130.0 (t, ^3J = 2.0 Hz), 129.7, 128.5, 128.4 (4C), 126.9, 126.1, 121.0, 120.4, 119.7 (t, ^1J = 240 Hz), 118.3, 116.0, 39.0 (t, ^2J = 24.9 Hz), 28.2 (t, ^3J = 4.5 Hz). **^{19}F NMR ($CDCl_3$, 282 MHz):** δ, ppm: -90.52 (t, *J* = 15.4 Hz). HRMS (ESI): calcd. for $C_{23}H_{17}N_2OF_2{}^{79}BrNa$: m/z $[M+Na]^+$ 477.03845; found: 477.0386 (0 ppm); calcd. for $C_{23}H_{16}N_2OF^{79}BrNa$: m/z $[M-HF+Na]^+$ 457.03222; found: 457.0342 (4 ppm).

4.8. Synthesis of (2-(1,1-difluoro-3-phenylpropyl)imidazo[1,2-a]pyridin-3-yl)(naphthalen-2-yl)methanone (7i)

The reaction was performed with **6c** (60 mg, 0.18 mmol) according to the general procedure. After 3h 30 min, ^{19}F NMR monitoring showed the absence of starting material. After purification by flash chromatography on silica gel, **7i** was isolated as white crystals (35 mg, 59% yield); **R_f** 0.23 (petroleum ether-EtOAc; 8:2); Mp: 132°C. **^{1}H NMR ($CDCl_3$, 300 MHz):** δ, ppm: 8.67 (d, 1H, *J* = 7.0 Hz), 8.26 (s, 1H), 7.86-7.94 (m, 2H), 7.77-7.84 (m, 2H), 7.68 (d, 1H, *J* = 9.1 Hz), 7.42-7.55 (m, 2H), 7.34 (m, 1H), 6.96-7.15 (m, 5H), 6.86-6.92 (t, 1H, *J* = 6.9 Hz), 2.58 (m, 4H). **^{13}C NMR ($CDCl_3$, 75 MHz):** δ, ppm: 187.7, 146.1, 144.9 (t, ^2J = 32.1 Hz), 140.3, 136.7 (t, ^3J = 2.2 Hz), 135.7, 132.1 (2C), 129.5, 128.6, 128.4, 128.3 (2C), 128.2 (2C), 127.8, 127.2, 126.9, 125.9, 124.5, 120.7, 120.3 (t, ^1J = 239.9 Hz), 118.1, 114.7 (2C), 39.2 (t, ^2J = 25.1 Hz), 28.2 (t, ^3J = 4.4 Hz). **^{19}F NMR ($CDCl_3$, 282 MHz):** δ, ppm: -90.71 (s). HRMS (ESI):

calcd. for $C_{27}H_{20}N_2OF_2Na$: m/z $[M+Na]^+$ 449.14359; found: 449.1430 (1 ppm); calcd. for $C_{27}H_{19}N_2OFNa$: m/z $[M-HF+Na]^+$ 429.13736; found: 429.1362 (3 ppm).

4.9. Synthesis of (2-(1,1-difluoro-3-phenylpropyl)imidazo[1,2-*a*]pyridin-3-yl)(4-methoxyphenyl)methanone (7j)

The reaction was performed with **6d** (15 mg, 0.05 mmol) according to the general procedure. After 6 h, ^{19}F NMR monitoring showed the absence of starting material. After purification by flash chromatography on silica gel, **7j** was isolated as white crystals (6 mg, 32% yield); **R*f*** 0.30 (petroleum ether-EtOAc; 8:2); Mp: 128°C. **^{1}H NMR ($CDCl_3$, 300 MHz):** δ, ppm: 8.58 (dt, 1H, J = 7.0, 1.1 Hz), 7.92 (m, 1H), 7.89 (m, 1H), 7.73 (dt, 1H, J = 9.1, 1.0 Hz), 7.37-7.45 (m, 1H), 7.21-7.26 (m, 2H), 7.13-7.18 (m, 3H), 6.95-6.99 (m, 3H), 3.90 (s, 3H), 2.60-2.77 (m, 4H). **^{13}C NMR ($CDCl_3$, 75 MHz):** δ, ppm: 186.4, 164.1, 145.8, 143.7 (t, ^3J = 32.3 Hz), 140.5, 132.2 (2C), 131.9, 128.3 (4C), 127.8, 127.0, 126.0, 120.7, 120.4 (t, ^1J = 238.9 Hz), 118.1, 114.4, 113.7 (2C), 55.5, 39.3 (t, ^2J = 25.1 Hz), 28.3 (t, ^3J = 4.4 Hz). **^{19}F NMR ($CDCl_3$, 282 MHz):** δ, ppm: -91.37 (t, t, J = 16.0 Hz). HRMS (ESI): calcd. for $C_{24}H_{20}N_2O_2F_2Na$: m/z $[M+Na]^+$ 429.1385; found: 429.1383 (0 ppm); calcd. for $C_{24}H_{19}N_2O_2FNa$: m/z $[M-HF+Na]^+$ 409.13228; found: 409.1337 (4 ppm).

5. General procedure of Suzuki–Miyaura coupling

A mixture of **7e** (1 equiv), phenylboronic acid (2 equiv), $PdCl_2(dppf)_2$ (0.09 equiv.), and Na_2CO_3 (1.3 equiv) in $EtOH/H_2O$ (1 mL/0.125 mL per 0.07 mmol) was heated under reflux. The reaction mixture was allowed to cool to room temperature and then diluted with EtOAc and water. The aqueous layer was

separated and extracted with EtOAc (3 times), the combined extracts were washed with water (3 times), dried over Na_2SO_4, filtered and concentrated. The residue was purified by chromatography on silica gel, using petroleum ether/ethyl acetate as eluent.

5.1. Synthesis of (2-(1,1-difluoro-3-phenylpropyl)-6-phenylimidazo[1,2-b]pyridazin-3-yl)(phenyl)methanone (8)

The reaction was performed with **7e** (30 mg, 0.07 mmol) according to the general procedure. After 16 h, ^{19}F NMR monitoring showed the absence of starting material. After purification by flash chromatography on silica gel, **8** was isolated as a yellow oil (14 mg, 46% yield); **R*f*** 0.14 (petroleum ether-EtOAc; 8:2).**^{1}H NMR ($CDCl_3$, 300 MHz):** δ, ppm: 8.26 (s, 1H), 8.23 (s, 1H), 8.09 (d, 1H, *J* = 9.6 Hz), 7.89 (s, 1H), 7.87 (s, 1H), 7.63-7.70 (m, 3H), 7.48-7.52 (m, 3H), 7.38-7.45 (m, 3H), 7.18-7.29 (m, 3H), 2.67-2.91 (m, 4H). **^{13}C NMR ($CDCl_3$, 75 MHz):** δ, ppm: 186.6, 152.8, 140.4, 137.8, 137.6, 135.6, 134.4, 133.8, 133.5, 132.7, 130.5, 129.7, 129.0, 128.6 (2C), 128.4 (4C), 128.0 (2C), 127.0, 126.3, 126.1, 120.3 (t, ^{1}J = 240.4 Hz), 118.5, 39.3 (t, ^{2}J = 25.4 Hz), 28.3 (t, ^{3}J = 4.3 Hz). **^{19}F NMR ($CDCl_3$, 282 MHz):** δ, ppm: -93.55 (t, *J* = 16.1 Hz). HRMS (ESI): calcd. for $C_{28}H_{21}N_3OF_2Na$: m/z $[M+Na]^+$ 476.15449; found: 476.1542 (1 ppm); calcd. for $C_{28}H_{20}N_3OFNa$: m/z $[M-HF+Na]^+$ 456.14826; found: 456.1478 (1 ppm).

5.2. Synthesis of [2-(1,1-Difluoro-3-phenylpropyl)-6-(4-methoxyphenyl)-imidazo[1,2-*b*]pyridazin-3-yl]phenylmethanone (9)

The reaction was performed with **7e** (8 mg, 0.02 mmol) according to the general procedure. After 16 h, TLC showed the absence of starting material. After purification by flash chromatography on silica gel, **9** was isolated as a yellow oil (5 mg, 53% yield); **R*f*** 0.22 (petroleum ether-EtOAc; 7:3).**^{1}H NMR ($CDCl_3$, 300 MHz):** δ, ppm: 7.96 (d, 1H, *J* = 9.6 Hz), 7.80 (m, 2H), 7.51-7.59 (m, 4H), 7.40-7.44 (m, 2H), 7.16-7.21 (m, 3H), 7.06-7.15 (m, 2H), 6.79-6.83 (m, 2H), 3.74 (s, 3H), 2.79-2.81 (m, 2H), 2.63-2.75 (m, 2H). **^{13}C NMR ($CDCl_3$, 125 MHz):** δ, ppm: 186.7, 161.6, 152.3, 142.4 (t, ^{2}J = 32.4

Hz), 140.4, 137.7, 137.6, 133.8, 129.7 (2C), 128.6 (2C), 128.4 (4C), 128.3 (2C), 126.7, 126.1, 126.0, 124.5, 120.3 (t, ^1J = 240.1 Hz), 118.2, 114.4 (2C), 55.4, 39.3 (t, ^2J = 25.4 Hz), 28.3 (t, ^3J = 4.1 Hz). **^{19}F NMR ($CDCl_3$, 470 MHz):** δ, ppm: -93.05 (t, J = 16.3 Hz). HRMS (ESI): calcd. for: m/z $C_{29}H_{23}N_3O_2F_2Na$: m/z $[M+Na]^+$ 506.16505; found: 506.1655 (1 ppm); calcd. for $C_{29}H_{22}N_3O_2FNa$: m/z $[M-HF+Na]^+$ 486.15883; found: 486.1588 (0 ppm).

6. General procedure for nucleophilic substitution of phenol

To a solution of pyridazin-2-one **7e** (1 equiv) in DMF (1 mL per 0.02 mmol) were added phenol (1.5 equiv) and K_2CO_3 (3 equiv). The mixture was stirred at 120°C. The reaction mixture was then extracted with EtOAc (3 times), the organic layers were separated, washed with H_2O (3 times), dried over Na_2SO_4, and concentrated under vacuum. After purification by chromatography on silica gel, using petroleum ether/ ethyl acetate as eluent.

6.1. Synthesis of (2-(1,1-difluoro-3-phenylpropyl)-6-phenoxyimidazo[1,2-*b*]pyridazin-3-yl)(phenyl)methanone (10)

The reaction was performed with **7e** (10 mg, 0.02 mmol) according to the general procedure. After 5 h, ^{19}F NMR monitoring showed the absence of starting material. After purification by flash chromatography on silica gel, **10** was isolated as a yellow oil (7 mg, 73% yield); **R_f** 0.50 (petroleum ether-EtOAc; 6:4).**^{1}H NMR ($CDCl_3$, 300 MHz):** δ, ppm: 7.80 (d, 1H, J = 9.7 Hz), 7.65 (s, 1H), 7.61-7.64 (m, 1H), 7.55-7.60 (m, 1H), 7.41 (m, 2H), 7.18-7.25 (m, 5H), 7.11-7.14 (m, 3H), 7.00-7.05 (d, 1H, J = 9.7 Hz), 6.84 (m, 1H), 6.87 (m, 1H), 2.64-2.91 (m, 4H). **^{13}C NMR ($CDCl_3$, 75 MHz):** δ, ppm: 186.1, 159.7, 152.1, 140.4, 137.1, 136.6, 133.7, 129.5 (2C), 129.4 (2C), 128.5 (4C), 128.4 (4C), 126.1, 125.4, 124.8, 120.5 (2C), 120.2 (t, ^1J = 240.5 Hz), 114.6, 39.5 (t, ^2J =

25.6 Hz), 28.4 (t, ^3J = 4.3 Hz). **^{19}F NMR ($CDCl_3$, 282 MHz):** δ, ppm: -93.78 (t, J = 16.1 Hz). HRMS (ESI): calcd. for $C_{28}H_{21}N_3O_2F_2Na$: m/z $[M+Na]^+$ 492.1494; found: 492.1491 (1 ppm); calcd. for $C_{28}H_{20}N_3O_2FNa$: m/z $[M-HF+Na]^+$ 472.14318; found: 472.1429 (1 ppm).

7. General procedure for nucleophilic substitution of morpholine

To a solution of pyridazin-2-one **7e** (1 equiv) in EtOH (1 mL per 0.10 mmol) were added morpholine (1.2 equiv) and Et_3N (5 equiv). The mixture was stirred at 80 °C. The solvent was then evaporated in vacuo and the residue was purified by flash column chromatography on silica gel.

7.1. Synthesis of (2-(1,1-difluoro-3-phenylpropyl)-6-morpholinoimidazo[1,2-*b*]pyridazin-3-yl)(phenyl)methanone (11)

The reaction was performed with **7e** (40 mg, 0.10 mmol) according to the general procedure. After 48 h, ^{19}F NMR monitoring showed the absence of starting material. After purification by flash chromatography on silica gel, **11** was isolated as a yellow oil (9 mg, 23% yield); **R*f*** 0.60 (petroleum ether-EtOAc; 7:3). **^{1}H NMR ($CDCl_3$, 300 MHz):** δ, ppm: 7.81-7.84 (m, 2H), 7.78-7.81 (m, 1H), 7.55-7.63 (m, 1H), 7.42-7.49 (m, 2H), 7.22-7.29 (m, 2H), 7.15-7.21 (m, 3H), 6.89-6.94 (d, 1H, J = 10.0 Hz), 3.67 (2t, 4H, J = 4.9 Hz), 3.22 (2t, 4H, J = 4.9 Hz), 2.65-2.89 (m, 4H). **^{13}C NMR ($CDCl_3$, 75 MHz):** δ, ppm: 186.9, 154.7, 140.5, 137.8, 135.8, 133.4, 129.5 (2C), 128.4 (4C), 128.3 (2C), 126.3, 126.0, 124.1, 120.3 (t, ^1J = 239.9 Hz), 112.0, 111.9, 66.1 (2C), 45.8 (2C), 39.4 (t, ^2J = 25.6 Hz), 28.4 (t, ^3J = 4.2 Hz). **^{19}F NMR ($CDCl_3$, 282 MHz):** δ, ppm: -93.24 (t, J = 15.9 Hz). HRMS (ESI): calcd. for $C_{26}H_{24}N_4O_2F_2Na$: m/z $[M+Na]^+$ 485.17595; found: 485.1759 (0 ppm); calcd. for $C_{26}H_{23}N_4O_2FNa$: m/z $[M-HF+Na]^+$ 465.16972; found: 465.1700 (1 ppm).

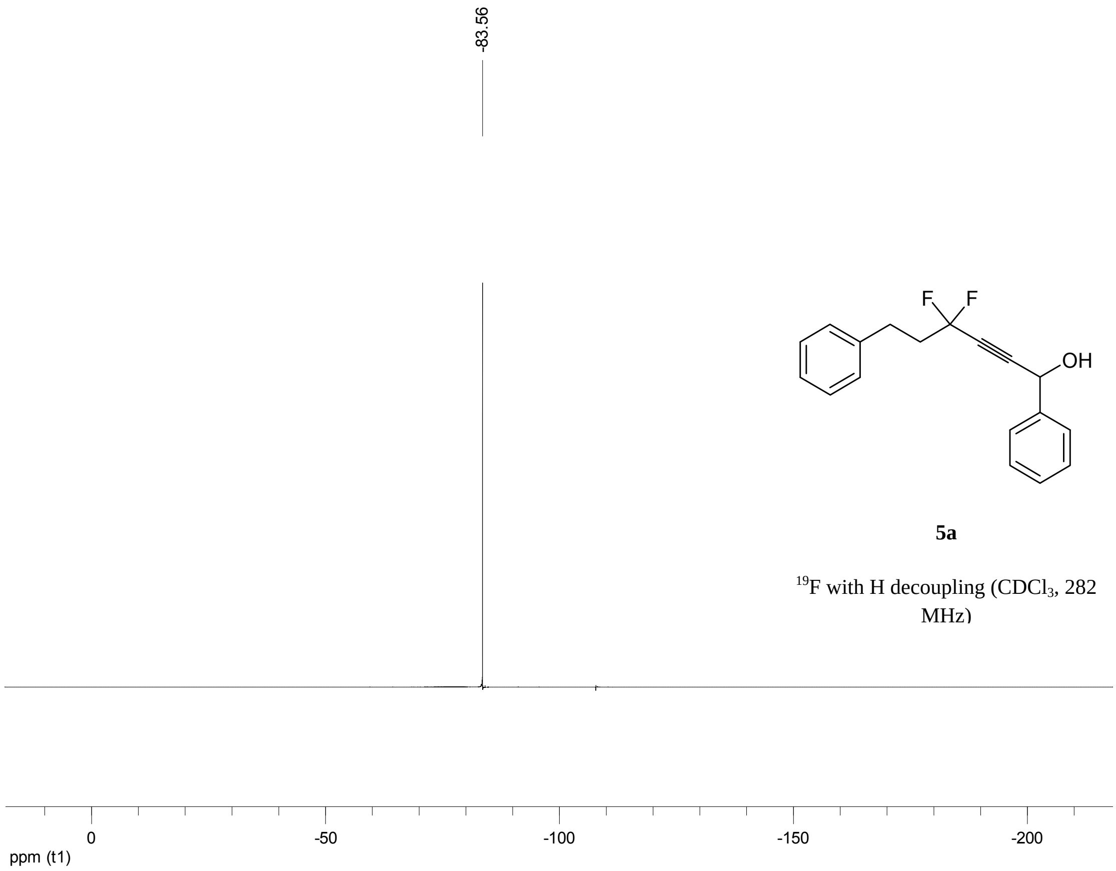

5a

^{19}F with H decoupling ($CDCl_3$, 282 MHz)

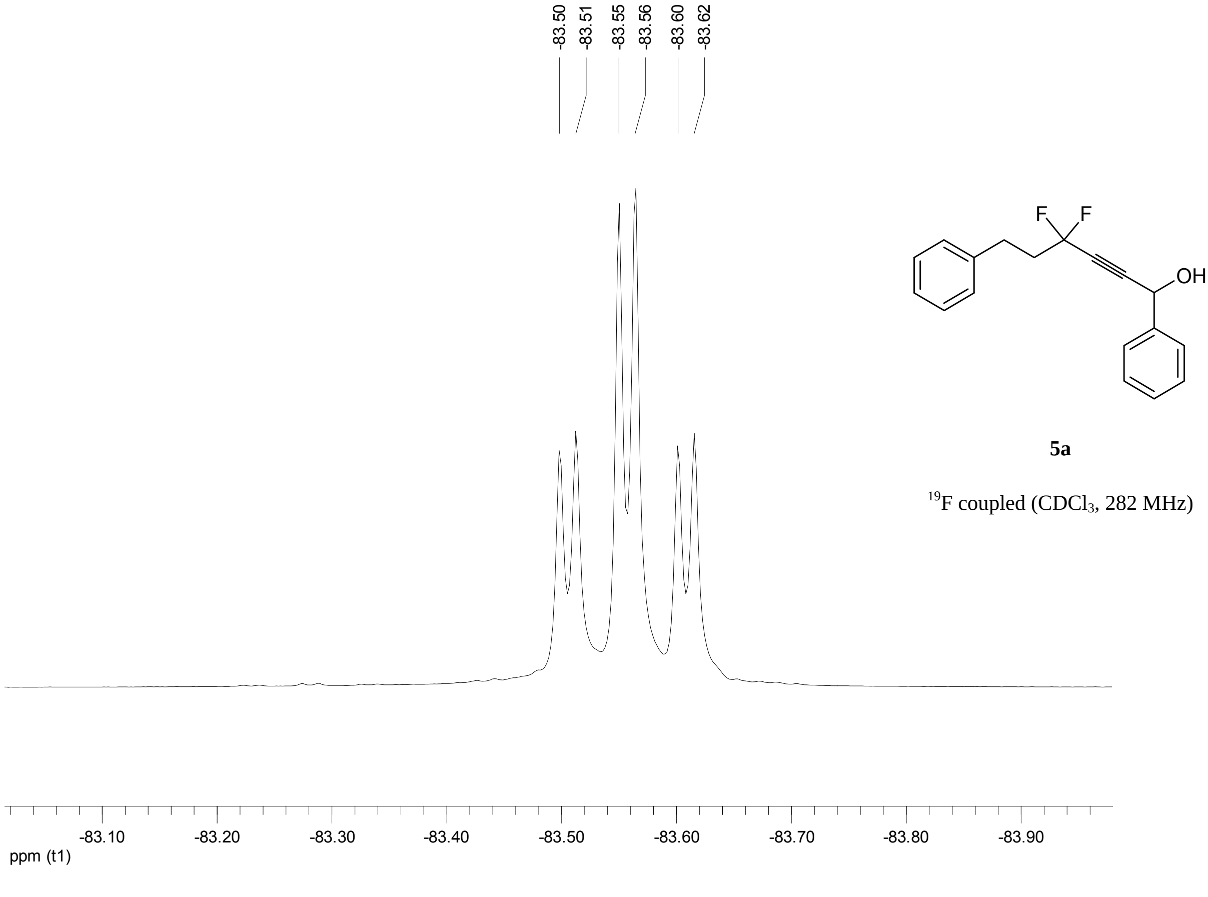

5a

^{19}F coupled ($CDCl_3$, 282 MHz)

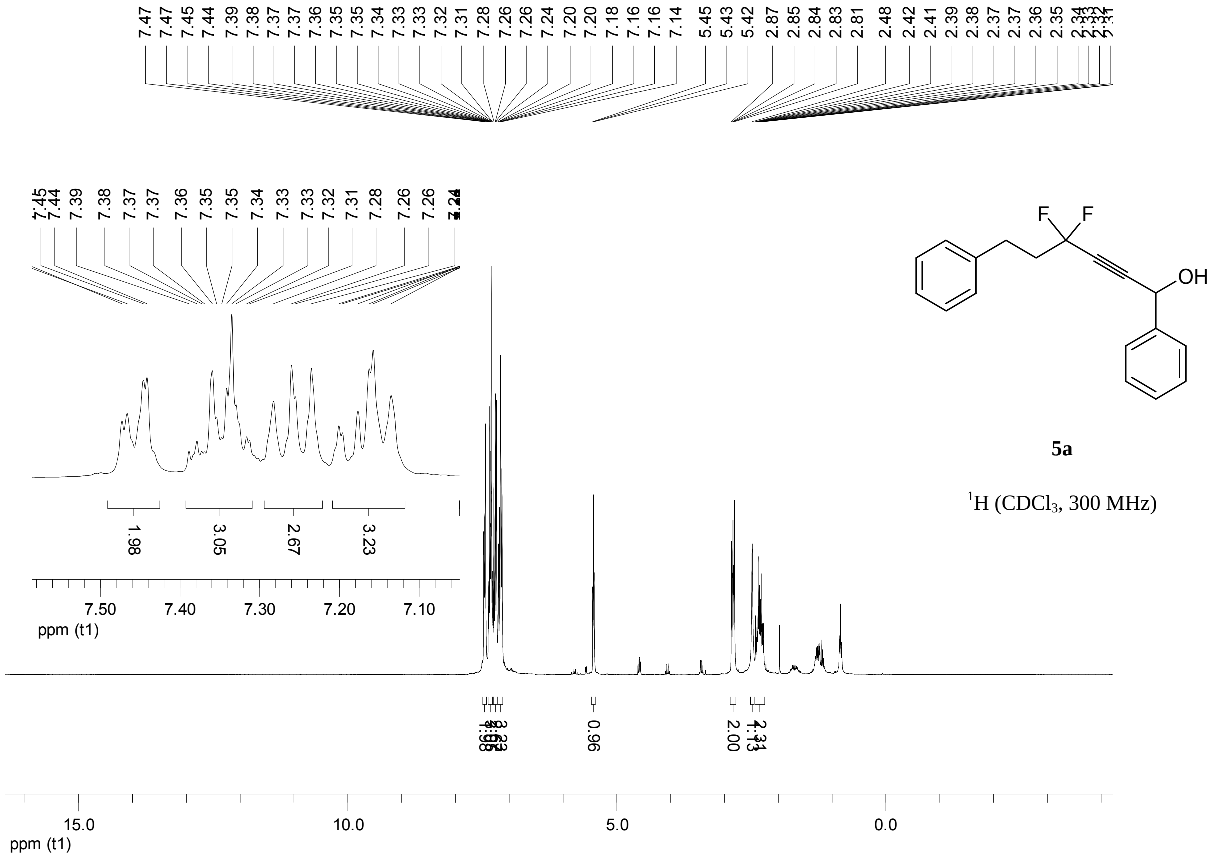

5a

^{1}H ($CDCl_3$, 300 MHz)

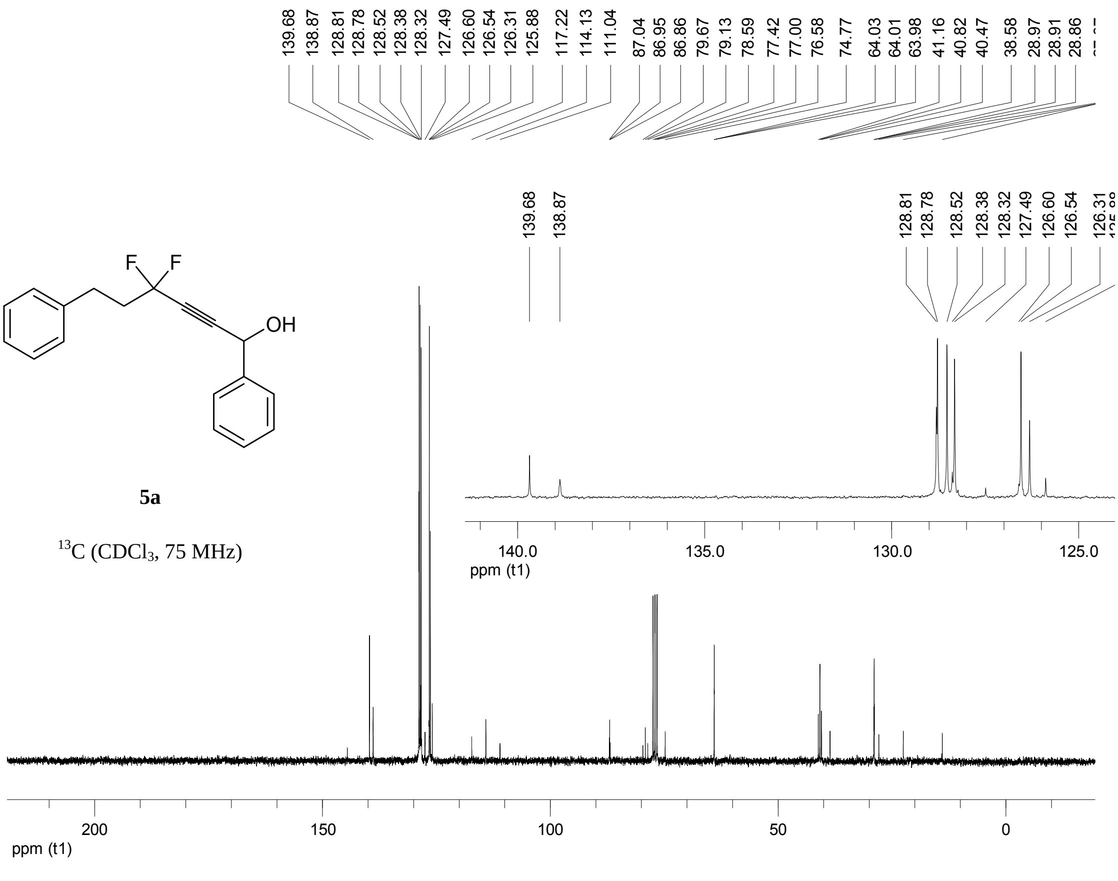

5a

^{13}C ($CDCl_3$, 75 MHz)

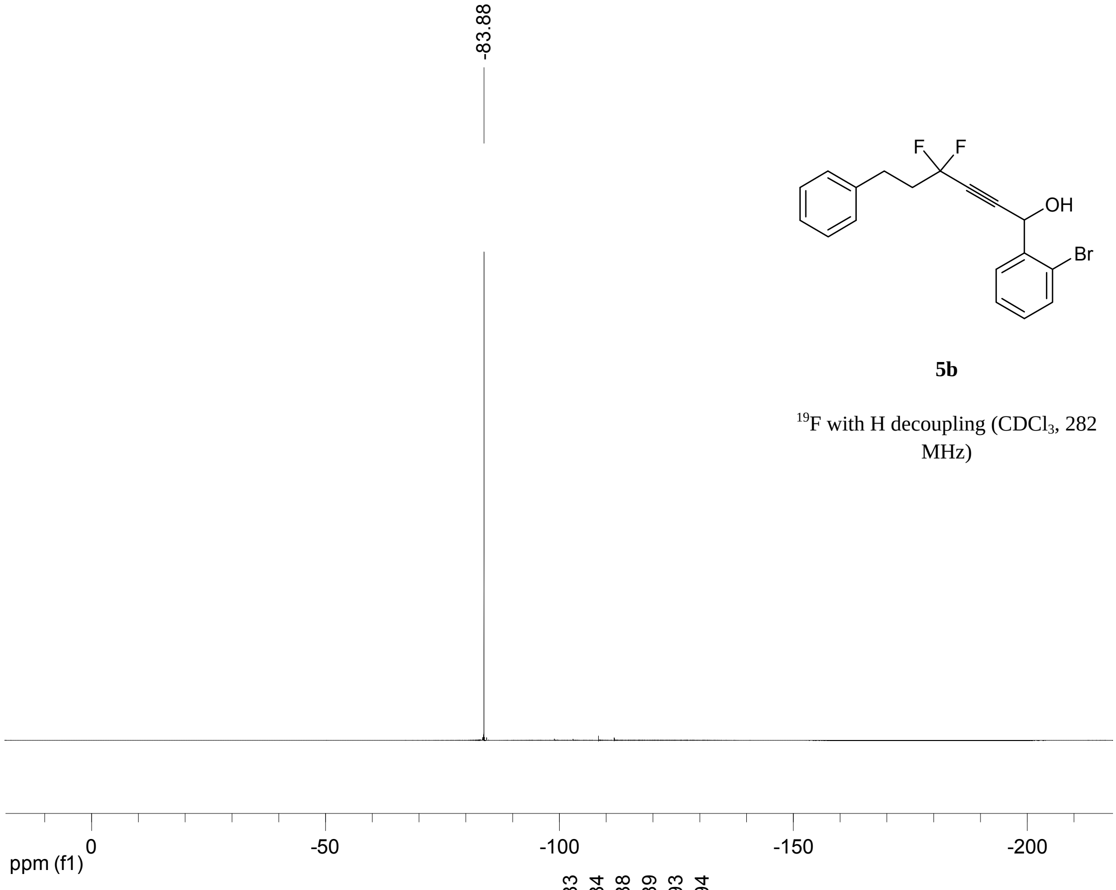

5b

^{19}F with H decoupling ($CDCl_3$, 282 MHz)

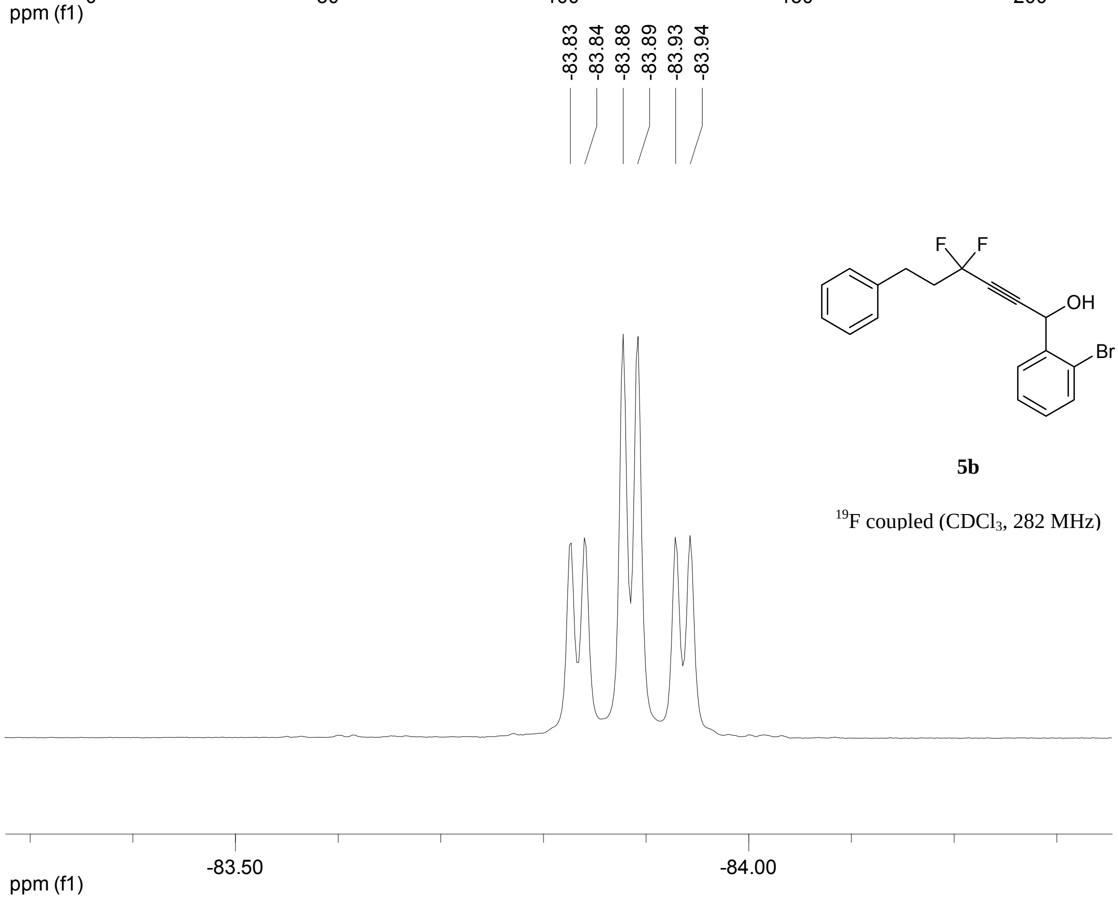

5b

^{19}F coupled ($CDCl_3$, 282 MHz)

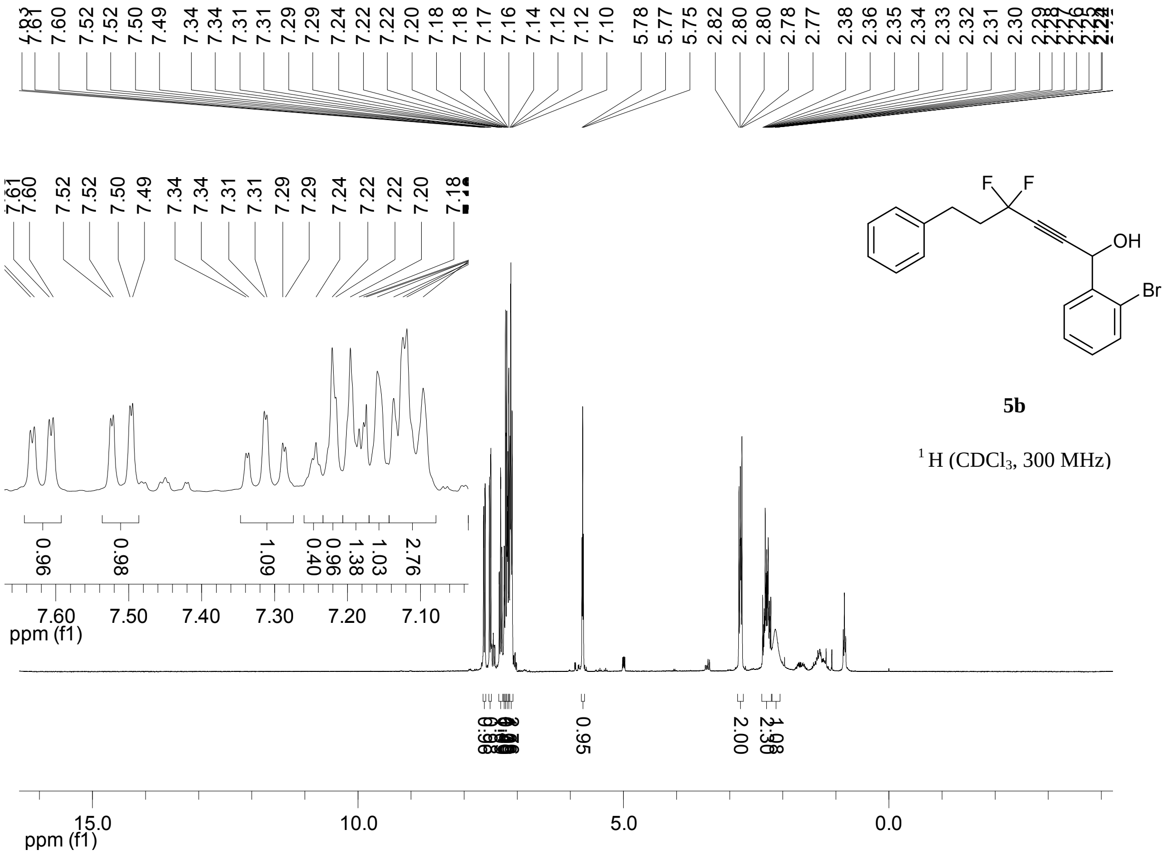

5b

^{1}H ($CDCl_3$, 300 MHz)

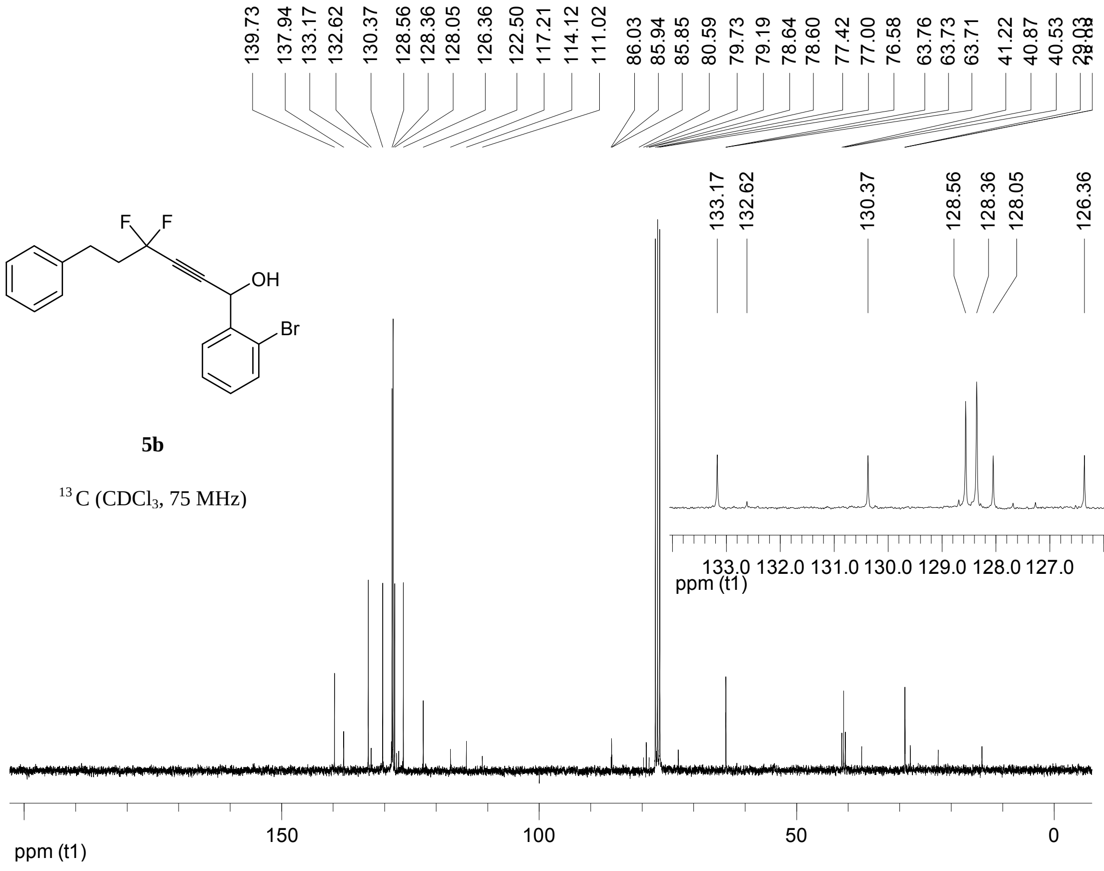

5b

^{13}C ($CDCl_3$, 75 MHz)

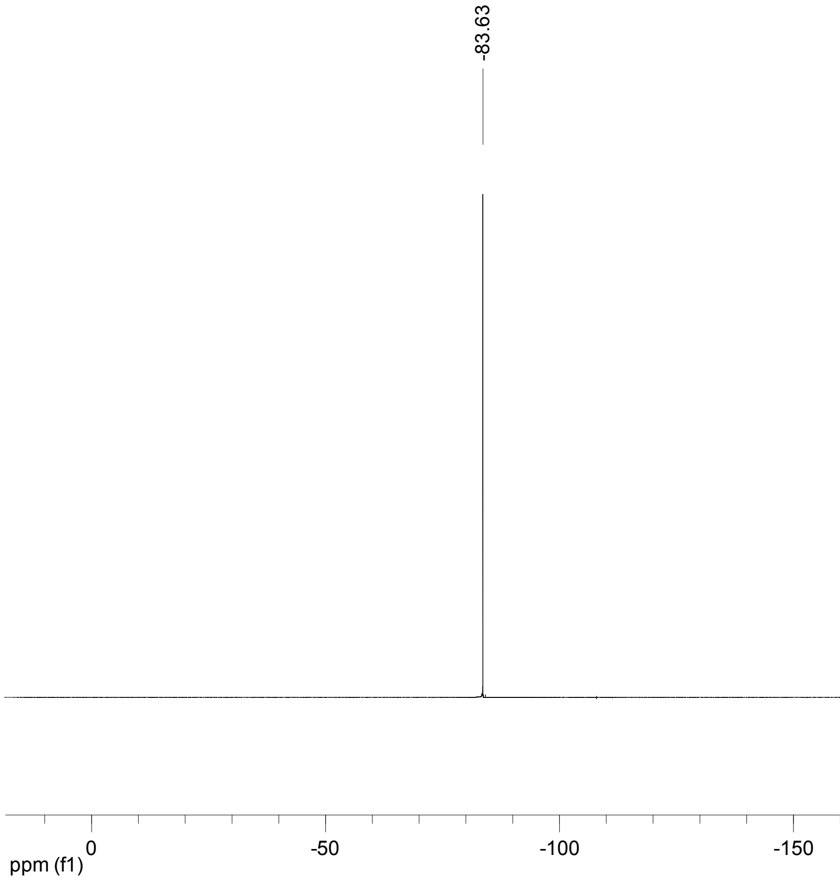

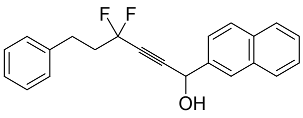

5c

^{19}F with H decoupling ($CDCl_3$, 282 MHz)

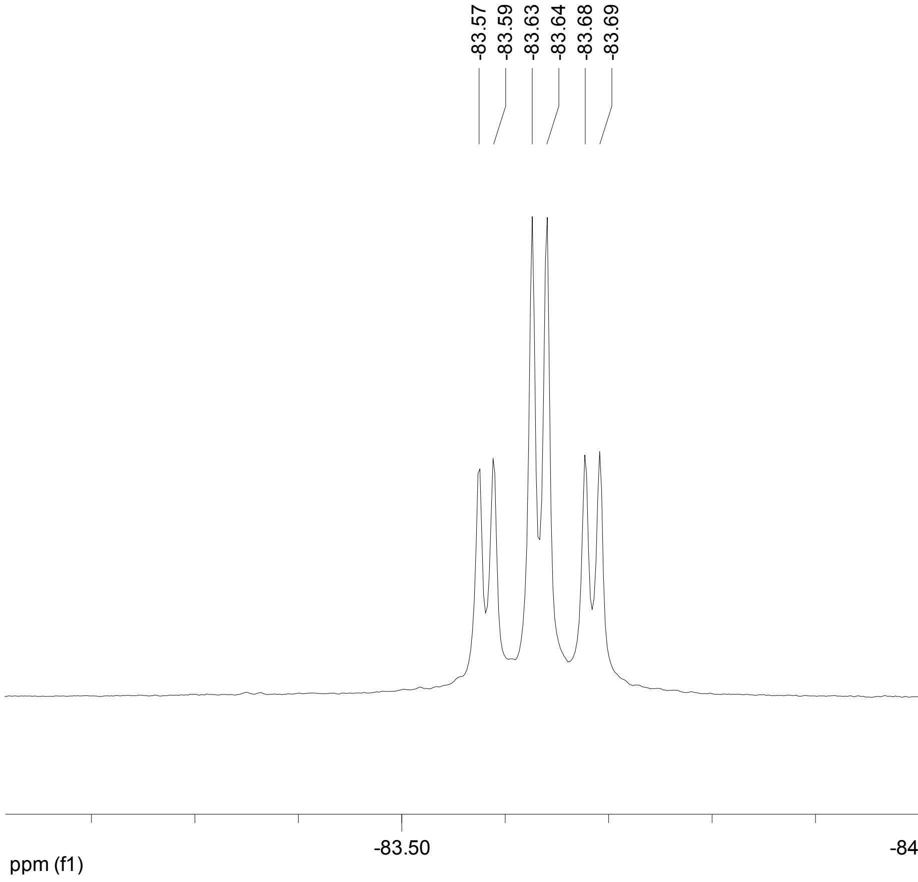

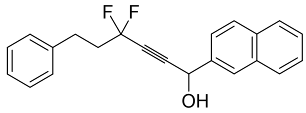

5c

^{19}F coupled ($CDCl_3$, 282 MHz)

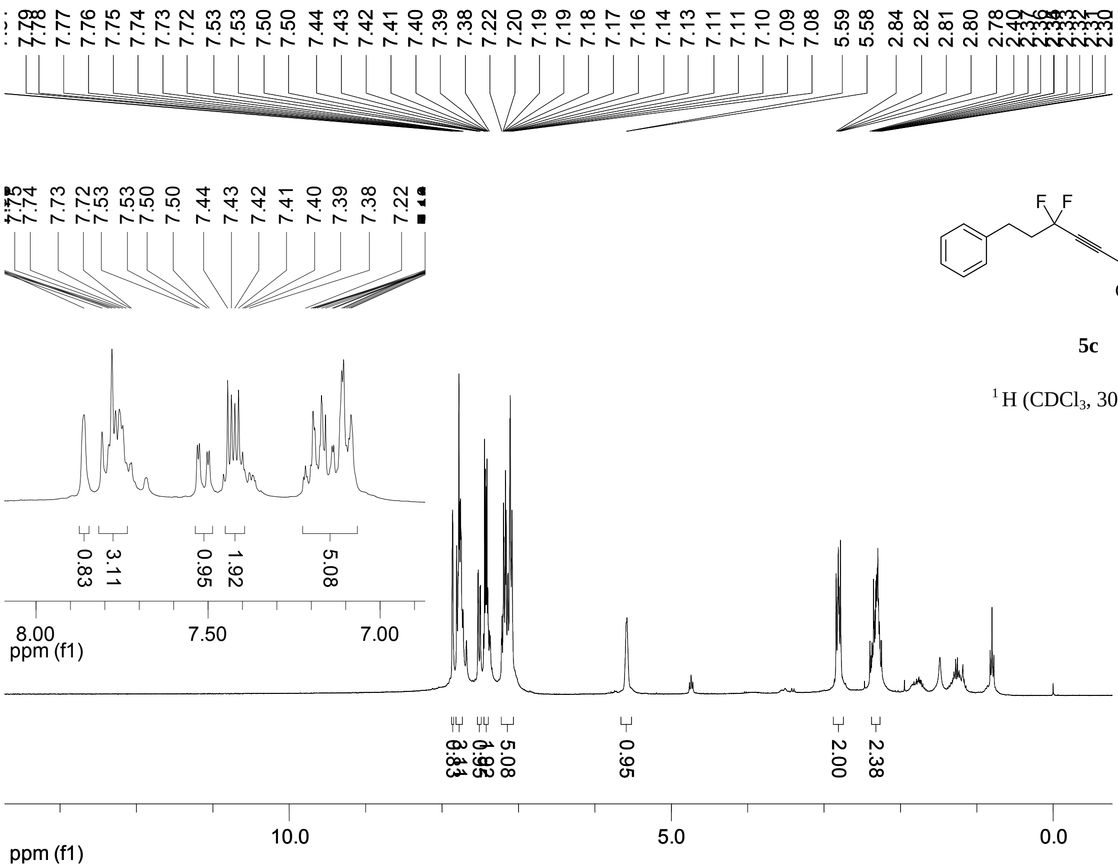

5c

^{1}H ($CDCl_3$, 300 MHz)

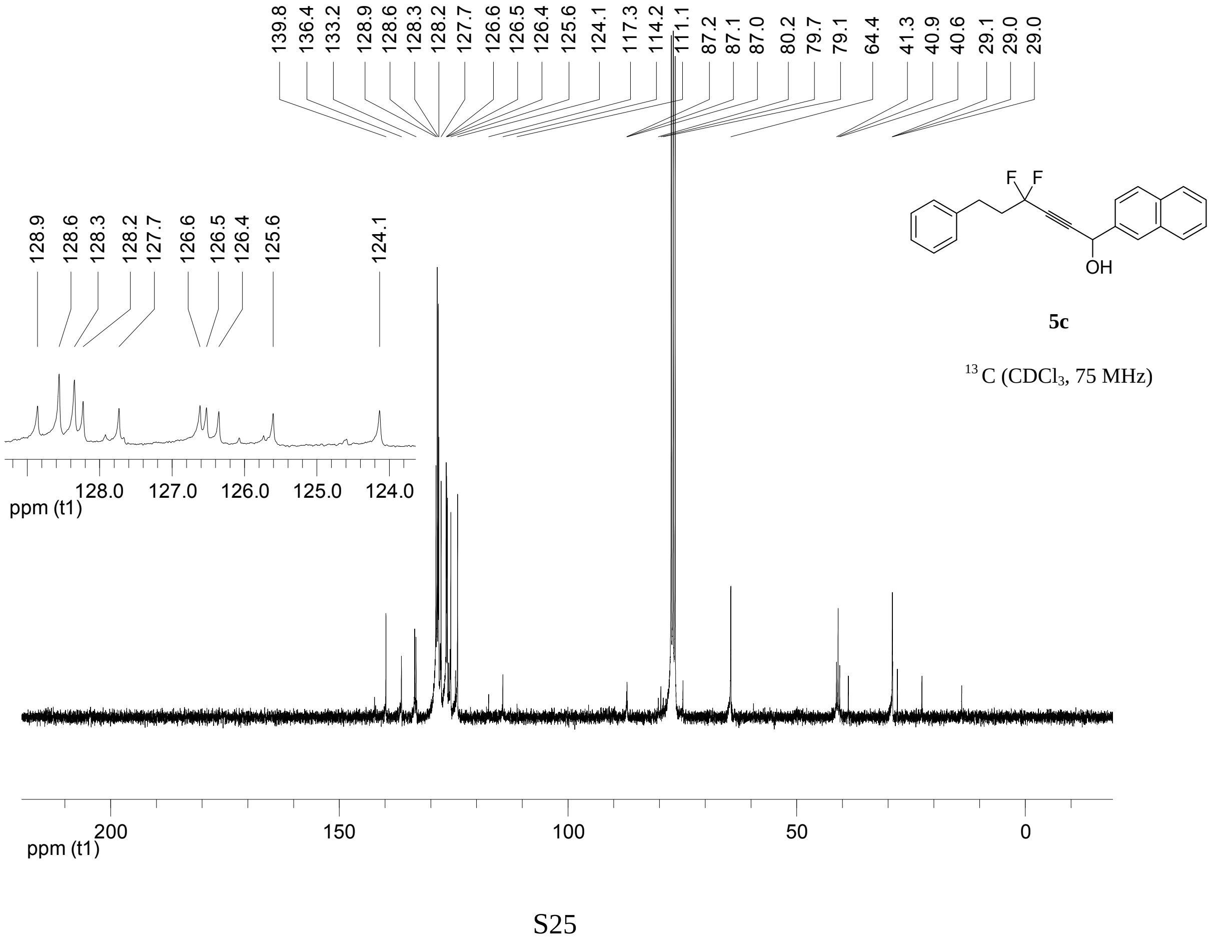

5c

^{13}C ($CDCl_3$, 75 MHz)

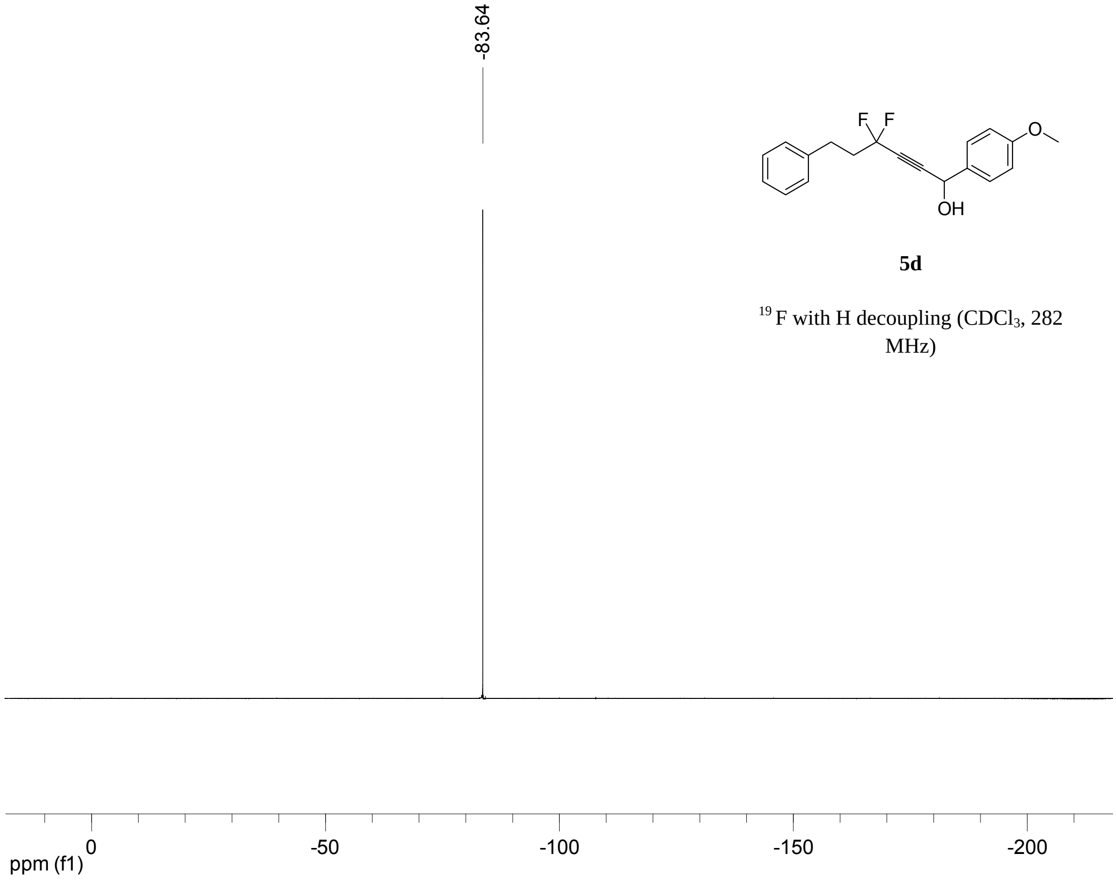

5d

[19] F with H decoupling ($CDCl_3$, 282 MHz)

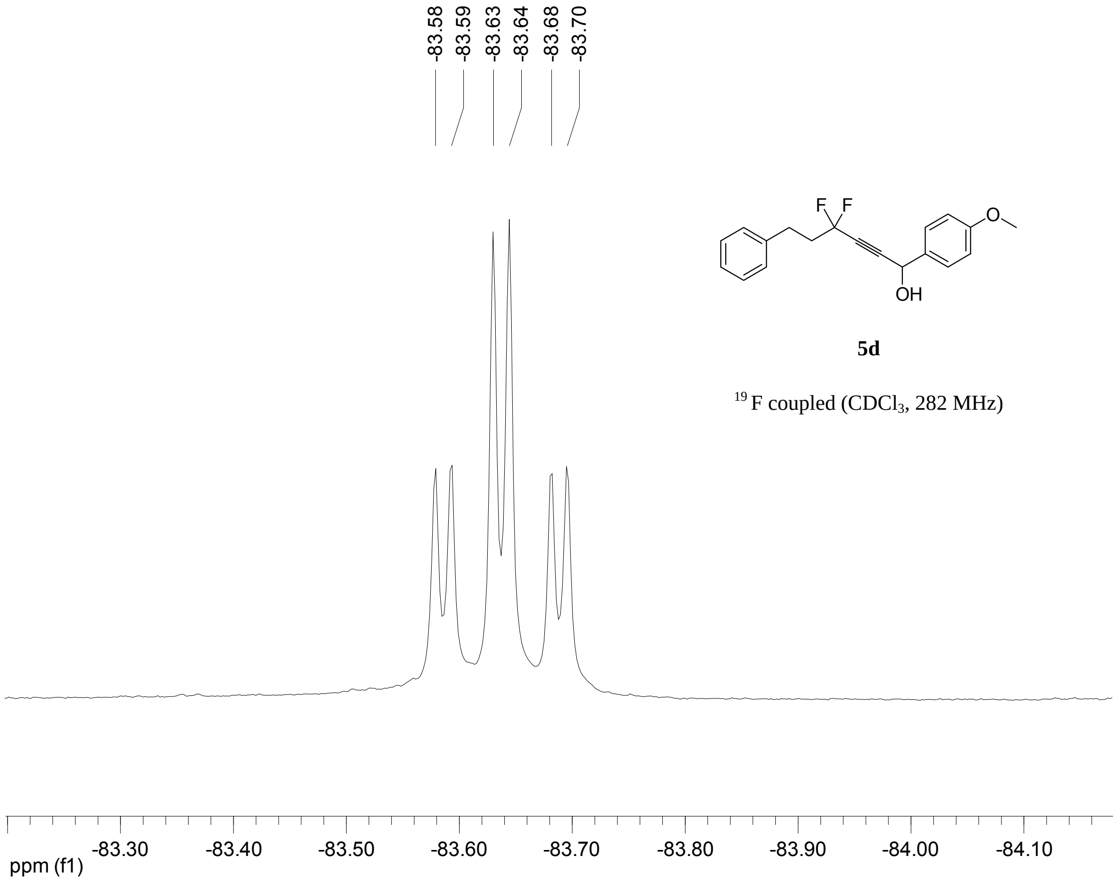

5d

[19] F coupled ($CDCl_3$, 282 MHz)

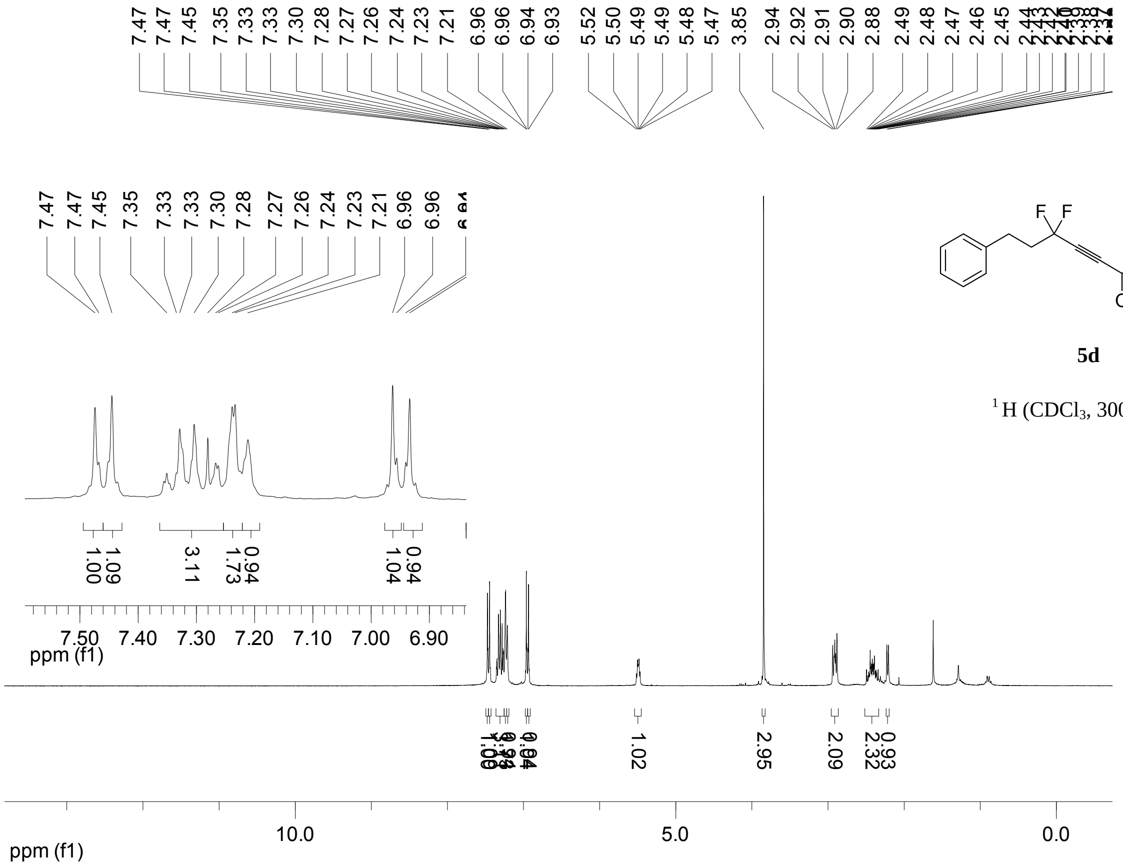

F F
O
OH
5d
^{1}H (CDCl$_3$, 300 MHz)
ppm (f1)
10.0
5.0
0.0

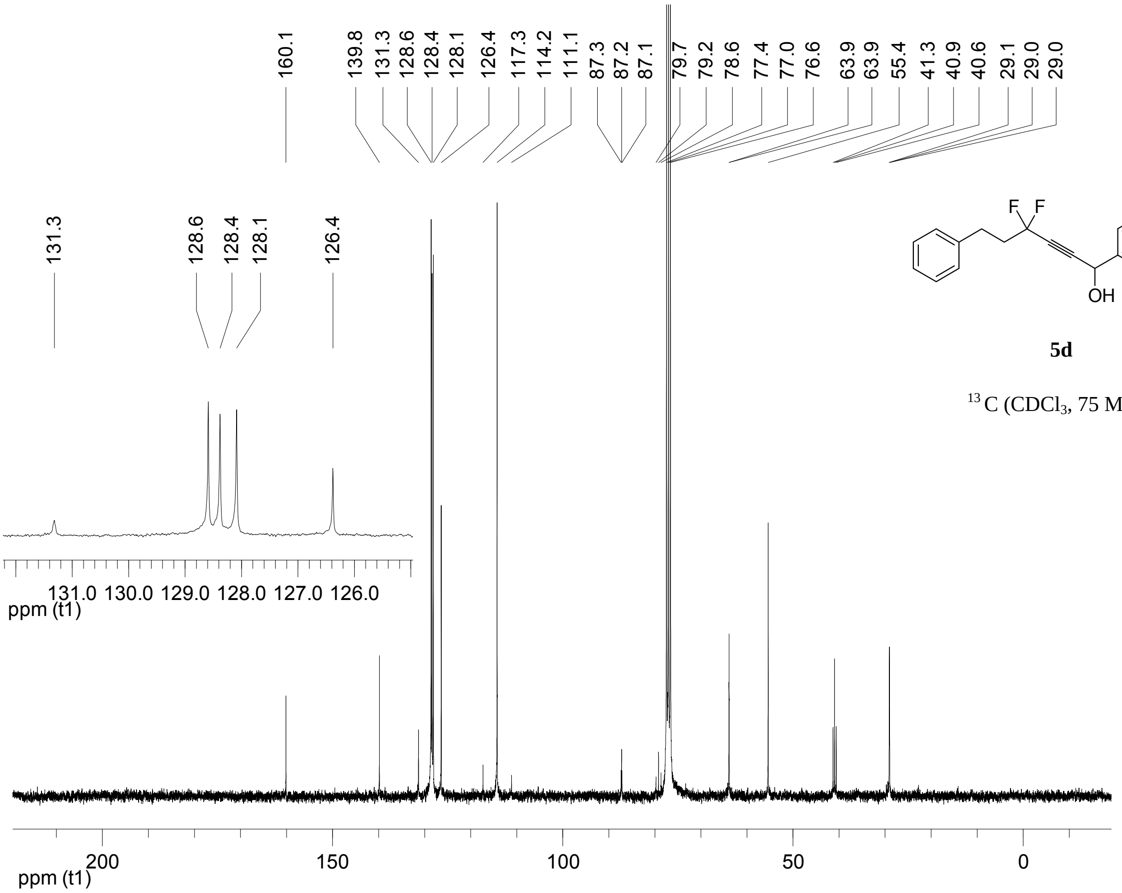

F F
O
OH
5d
^{13}C (CDCl$_3$, 75 MHz)
ppm (t1)
200
150
100
50
0

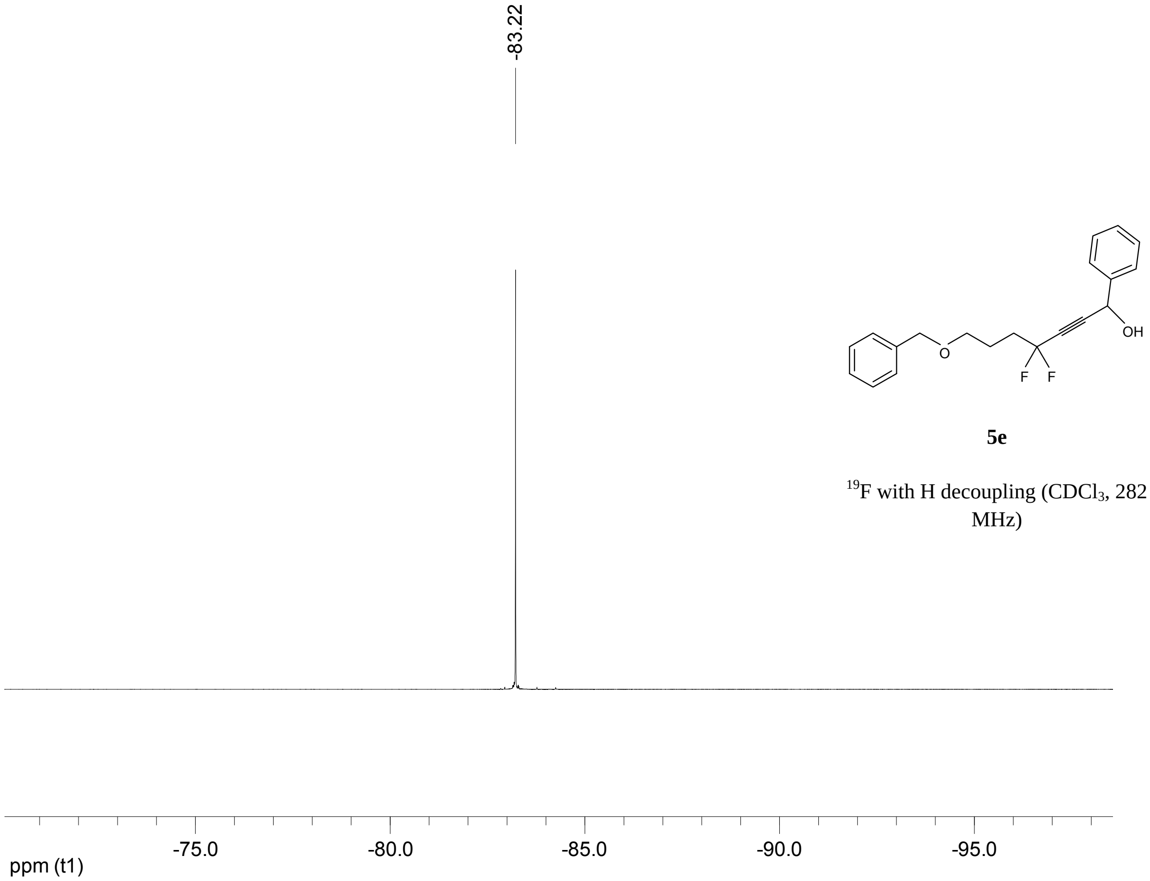

5e

^{19}F with H decoupling ($CDCl_3$, 282 MHz)

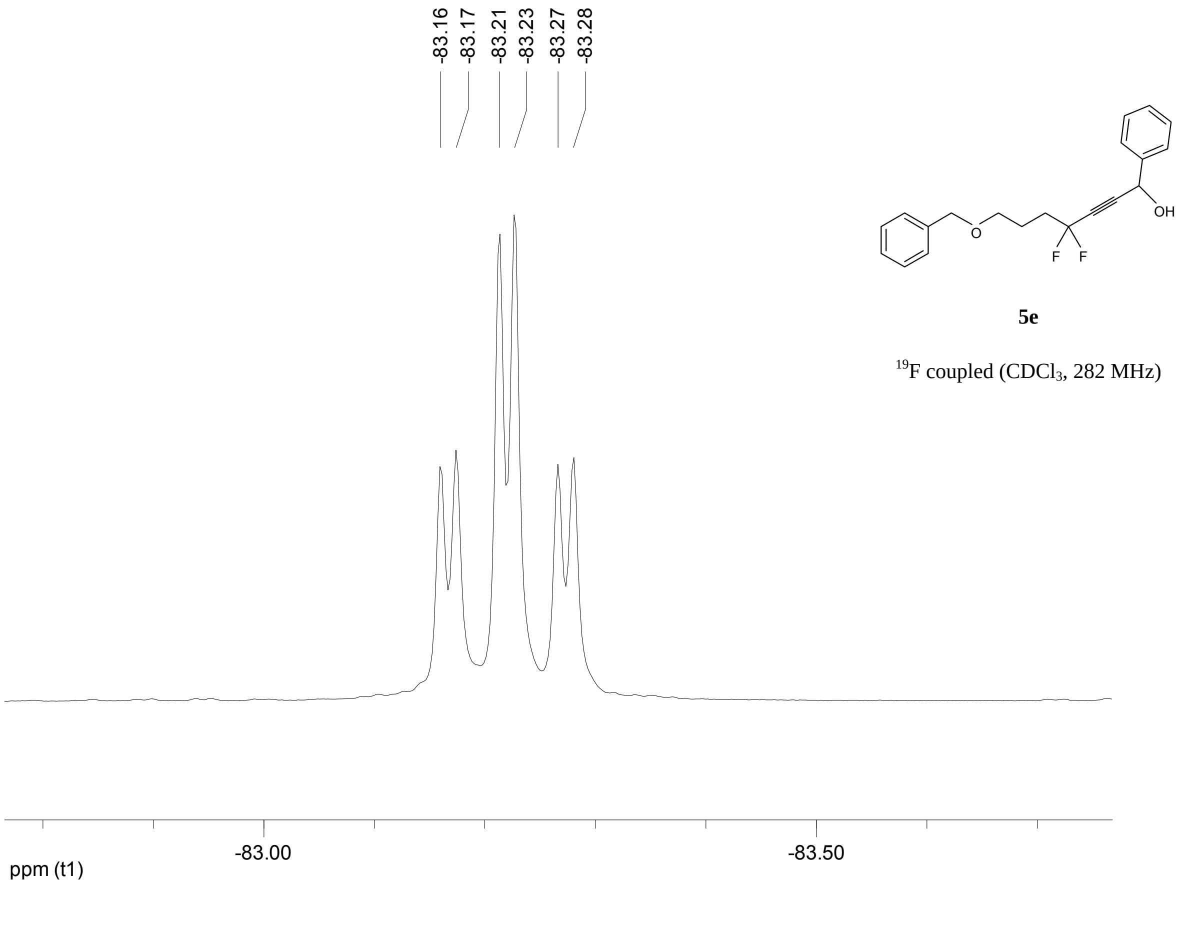

5e

^{19}F coupled ($CDCl_3$, 282 MHz)

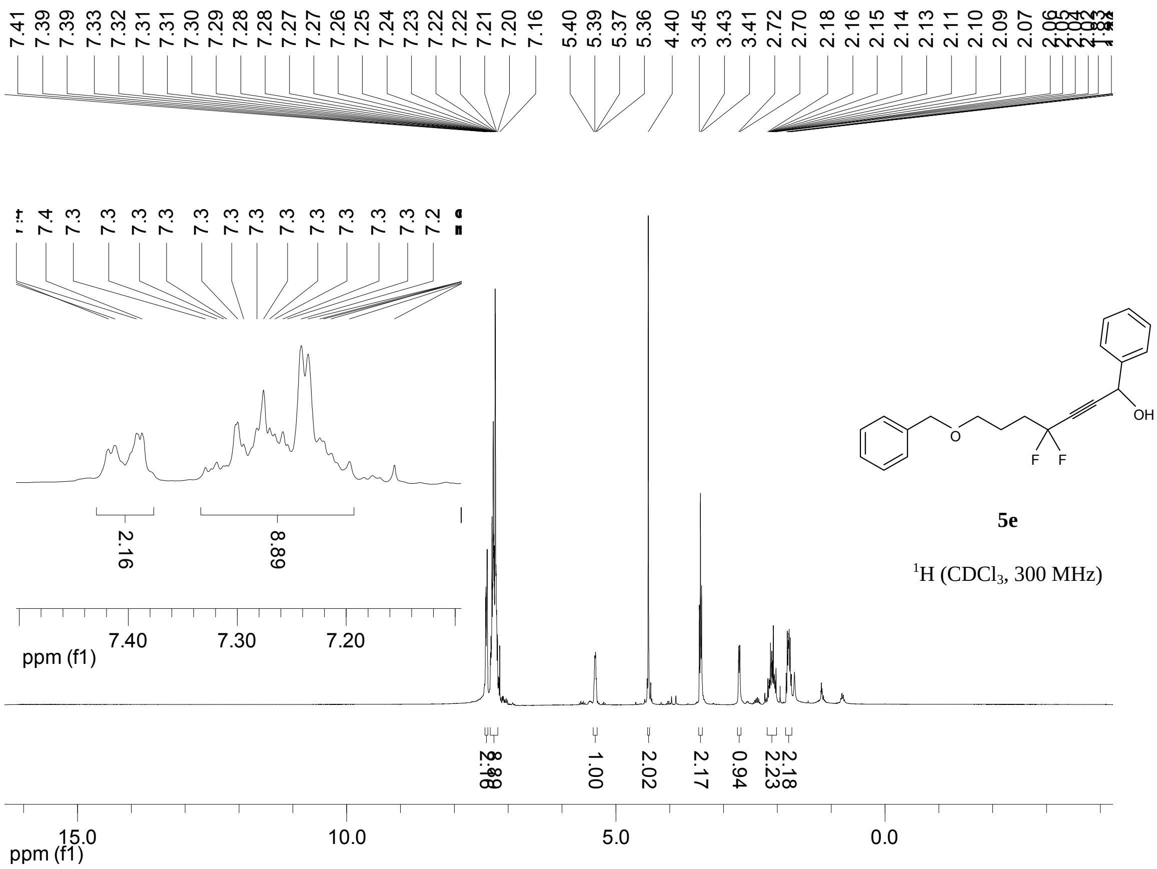

5e

^{1}H ($CDCl_3$, 300 MHz)

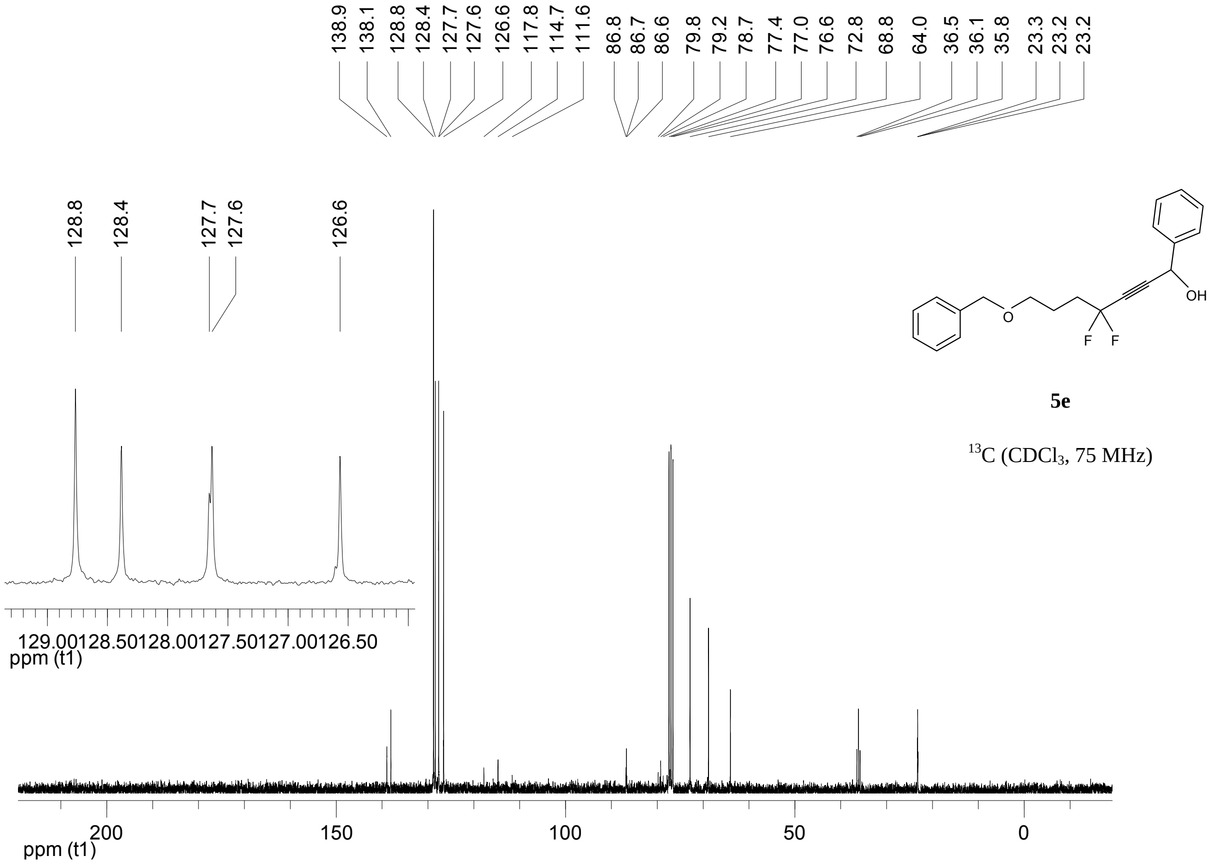

5e

^{13}C ($CDCl_3$, 75 MHz)

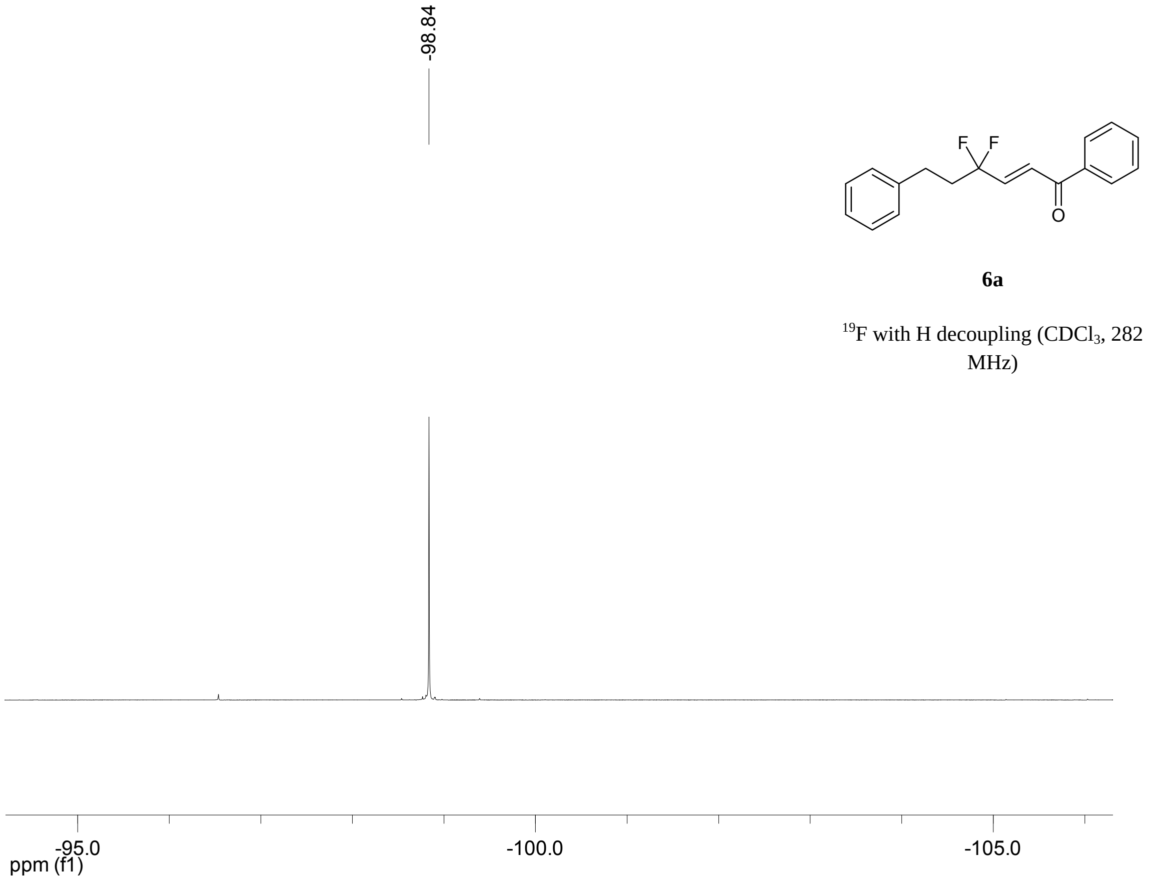

6a

^{19}F with H decoupling ($CDCl_3$, 282 MHz)

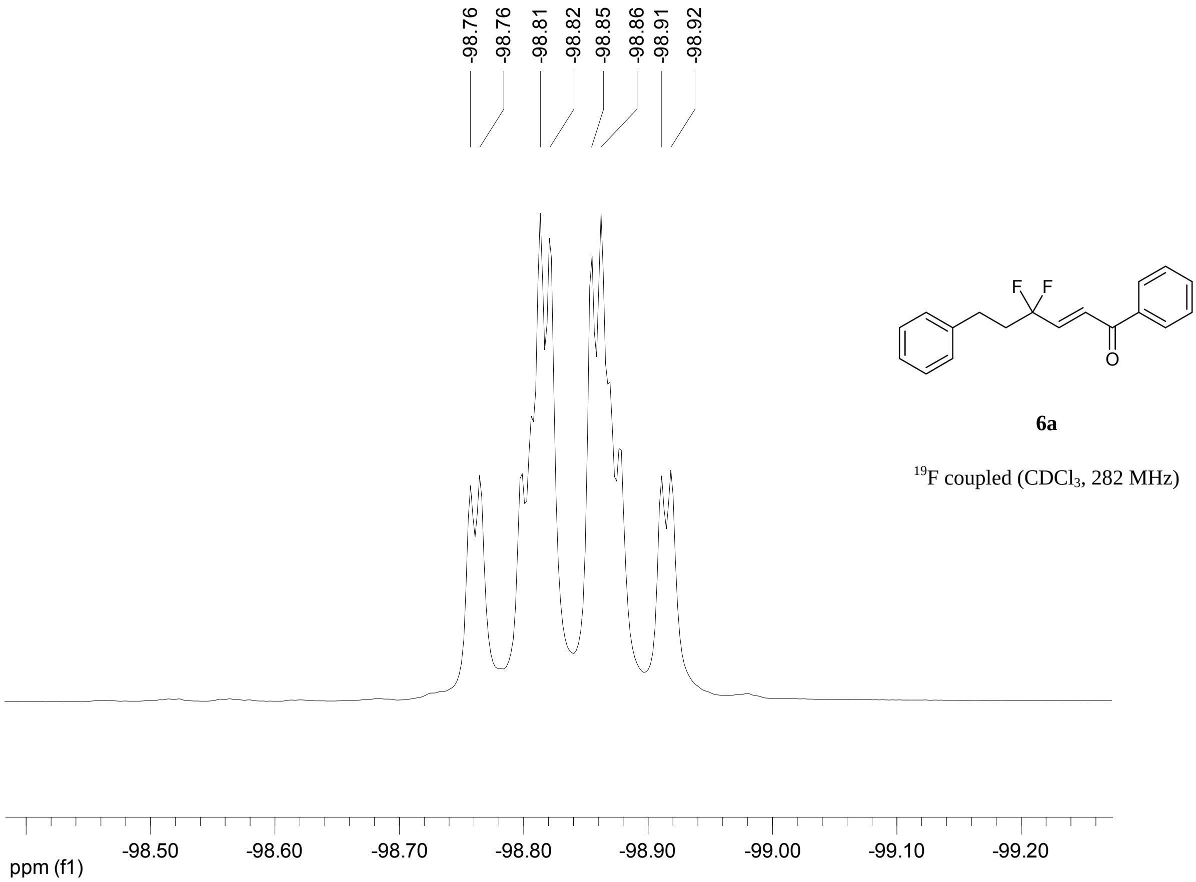

6a

^{19}F coupled ($CDCl_3$, 282 MHz)

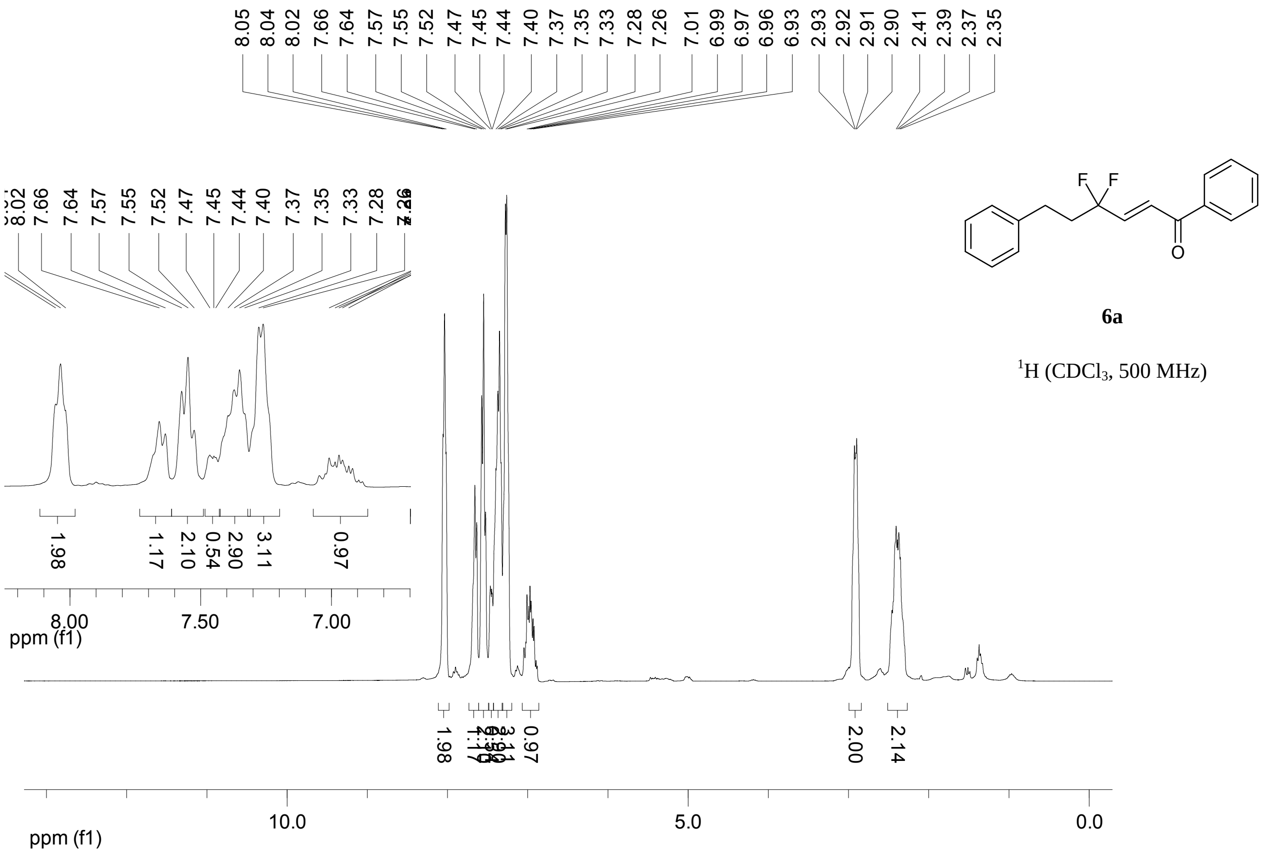
F F
O
6a
1H (CDCl3, 500 MHz)
ppm (f1)
10.0
5.0
0.0
8.00
7.50
7.00
1.98
1.17
2.10
0.54
2.90
3.11
0.97
2.00
2.14
8.05 8.04 8.02 7.66 7.64 7.57 7.55 7.52 7.47 7.45 7.44 7.40 7.37 7.35 7.33 7.28 7.26 7.01 6.99 6.97 6.96 6.93 2.93 2.92 2.91 2.90 2.41 2.39 2.37 2.35

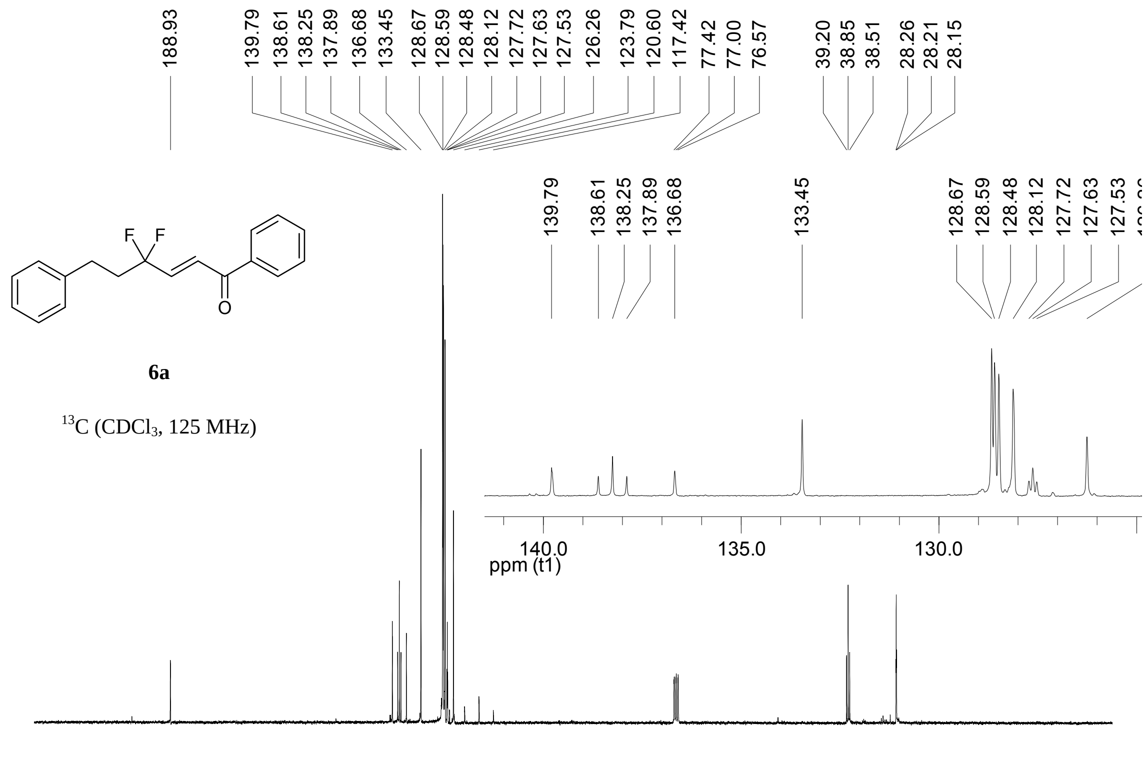
F F
O
6a
13C (CDCl3, 125 MHz)
ppm (t1)
200
150
100
50
0
140.0
135.0
130.0
188.93
139.79 138.61 138.25 137.89 136.68 133.45 128.67 128.59 128.48 128.12 127.72 127.63 127.53 126.26 123.79 120.60 117.42 77.42 77.00 76.57 39.20 38.85 38.51 28.26 28.21 28.15

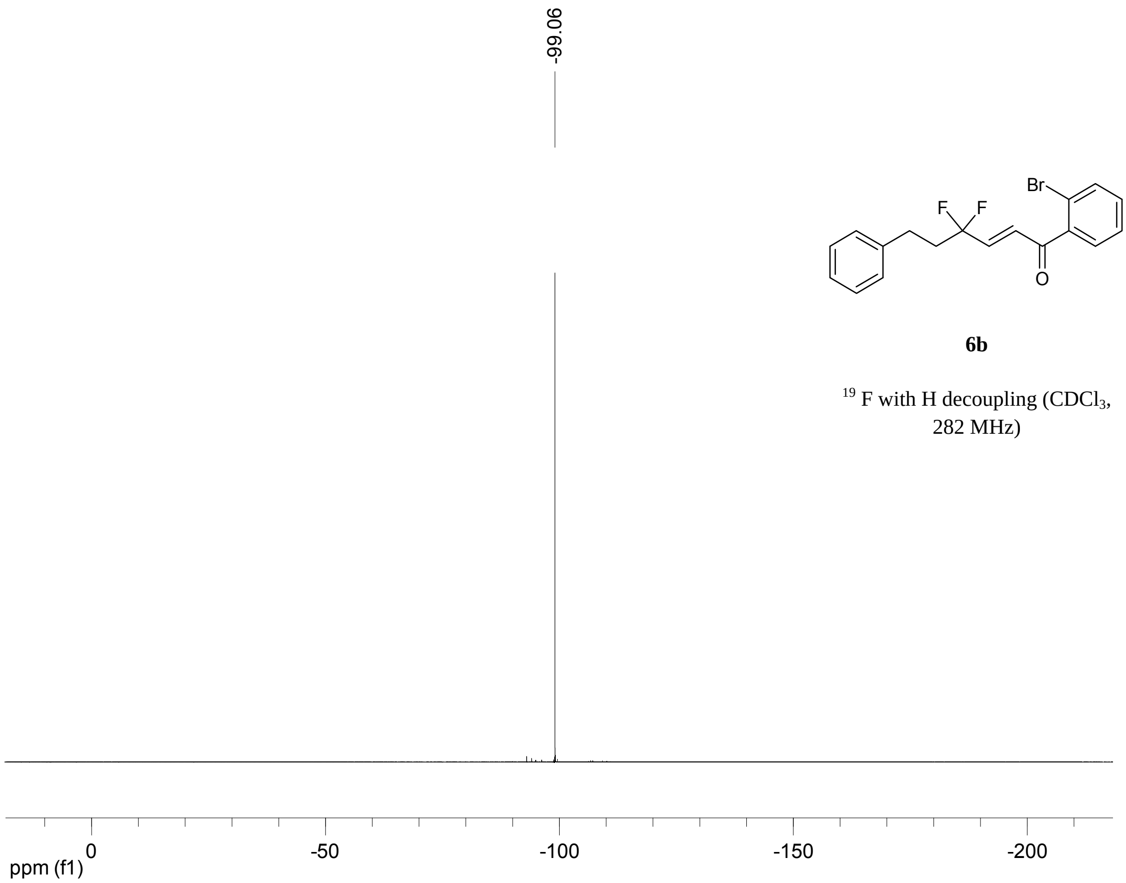

6b

[19] F with H decoupling ($CDCl_3$, 282 MHz)

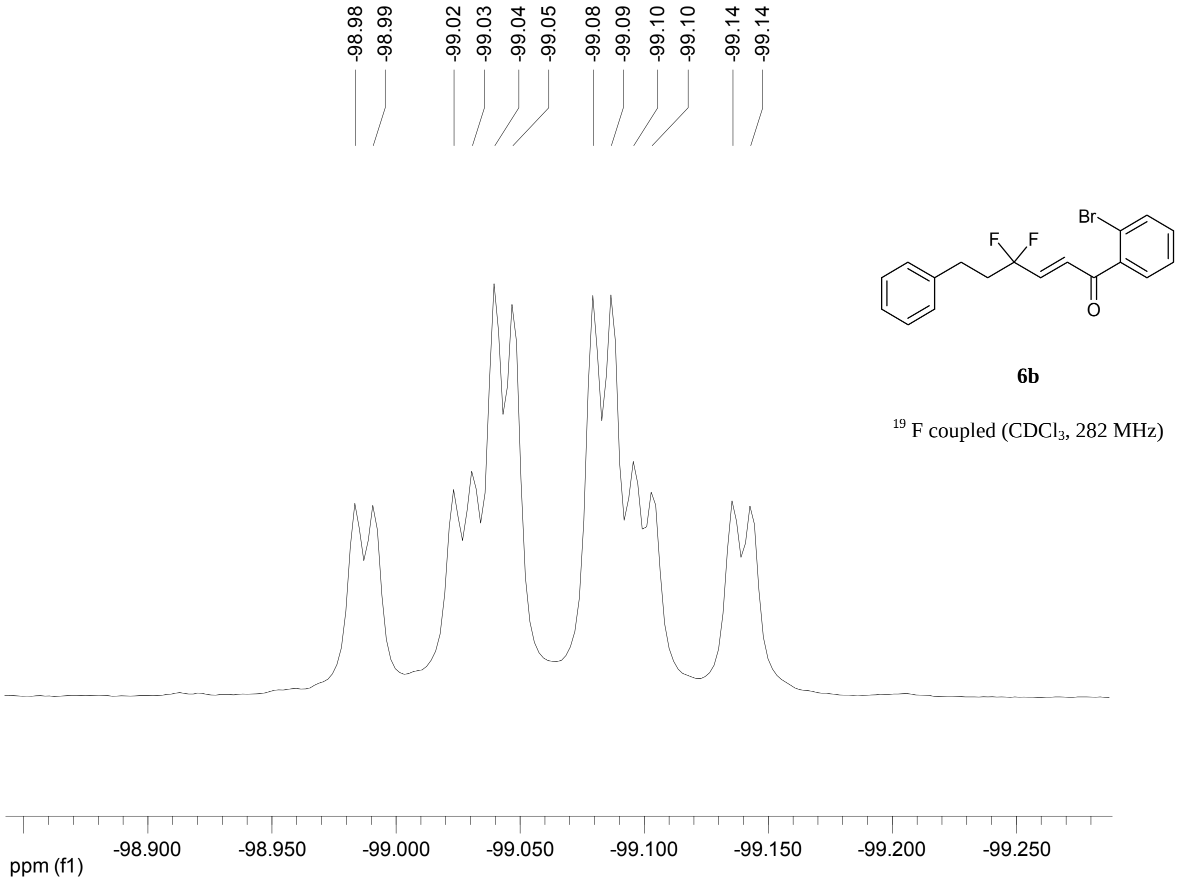

6b

[19] F coupled ($CDCl_3$, 282 MHz)

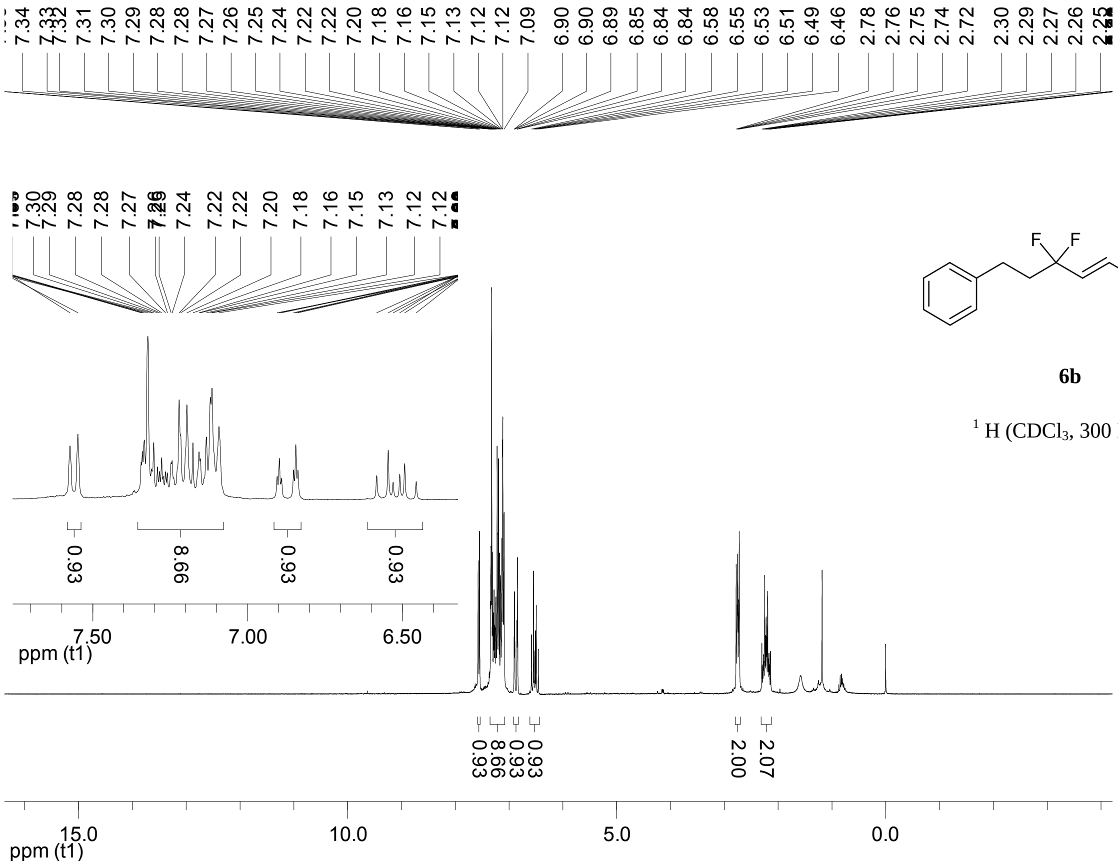
7.34 7.33 7.32 7.31 7.30 7.29 7.28 7.28 7.27 7.26 7.25 7.24 7.22 7.22 7.20 7.18 7.16 7.15 7.13 7.12 7.12 7.09 6.90 6.90 6.89 6.85 6.84 6.84 6.58 6.55 6.53 6.51 6.49 6.46 2.78 2.76 2.75 2.74 2.72 2.30 2.29 2.27 2.26 2.25
7.30 7.29 7.28 7.28 7.27 7.26 7.24 7.22 7.22 7.20 7.18 7.16 7.15 7.13 7.12 7.12
Br
F F
O
6b
^{1}H ($CDCl_3$, 300 MHz)
0.93 8.66 0.93 0.93
7.50 7.00 6.50
ppm (t1)
0.93 8.66 0.93 0.93 2.00 2.07
15.0 10.0 5.0 0.0
ppm (t1)

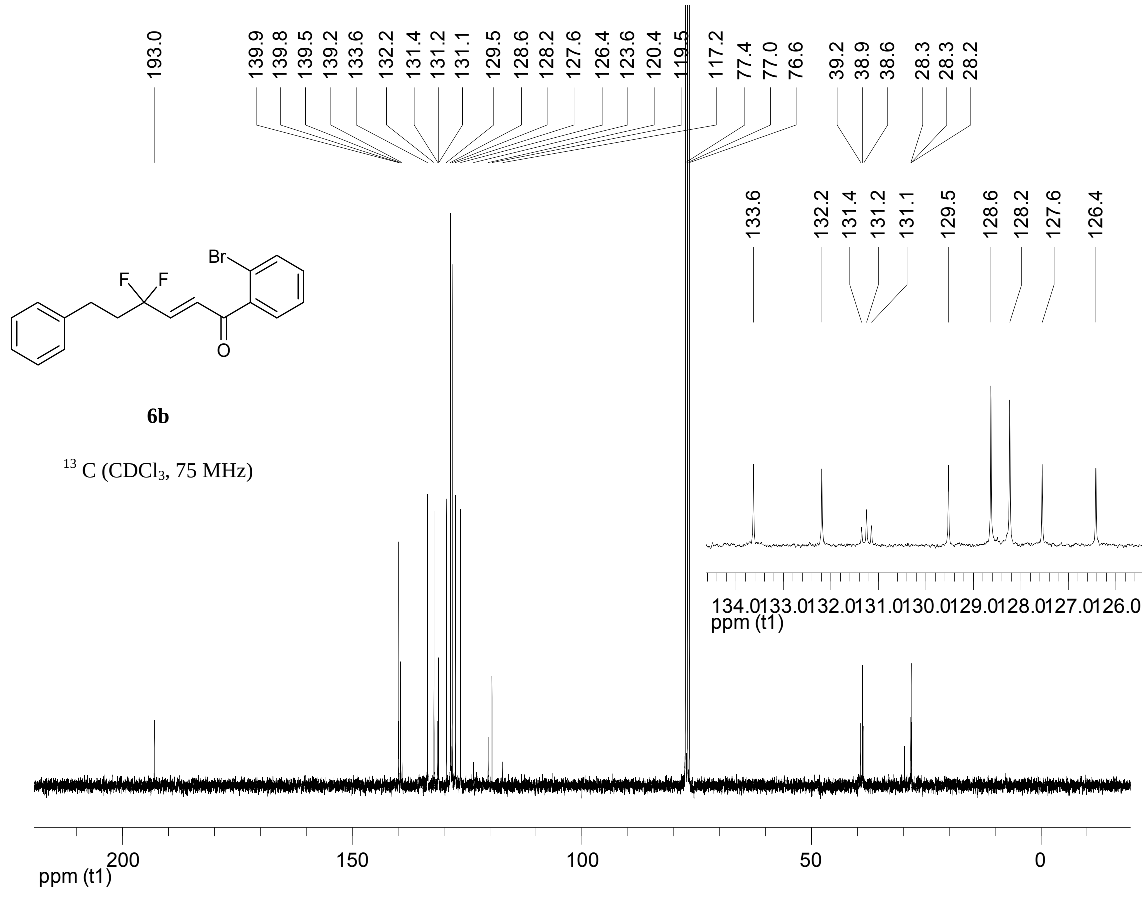
193.0
139.9 139.8 139.5 139.2 133.6 132.2 131.4 131.2 131.1 129.5 128.6 128.2 127.6 126.4 123.6 120.4 119.5 117.2 77.4 77.0 76.6
39.2 38.9 38.6 28.3 28.3 28.2
133.6 132.2 131.4 131.2 131.1 129.5 128.6 128.2 127.6 126.4
Br
F F
O
6b
^{13}C ($CDCl_3$, 75 MHz)
134.0133.0132.0131.0130.0129.0128.0127.0126.0
ppm (t1)
200 150 100 50 0
ppm (t1)

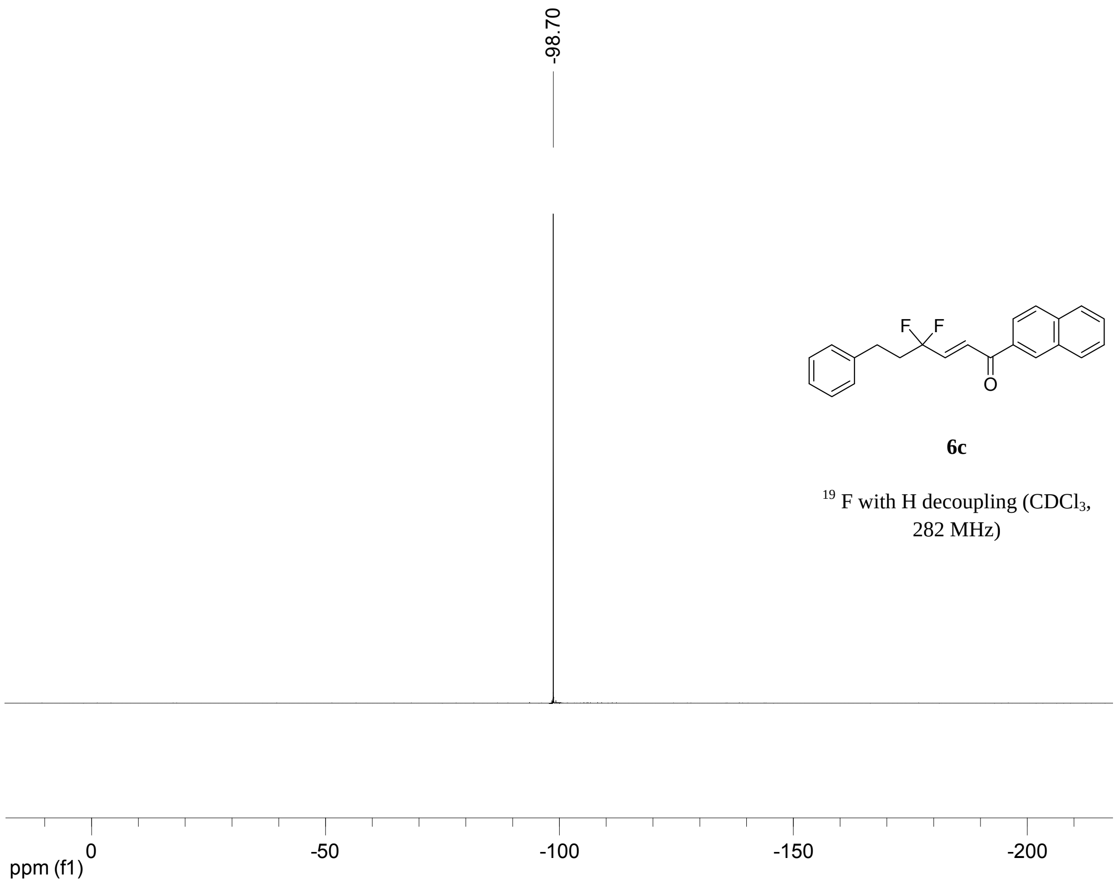

6c

[19] F with H decoupling ($CDCl_3$, 282 MHz)

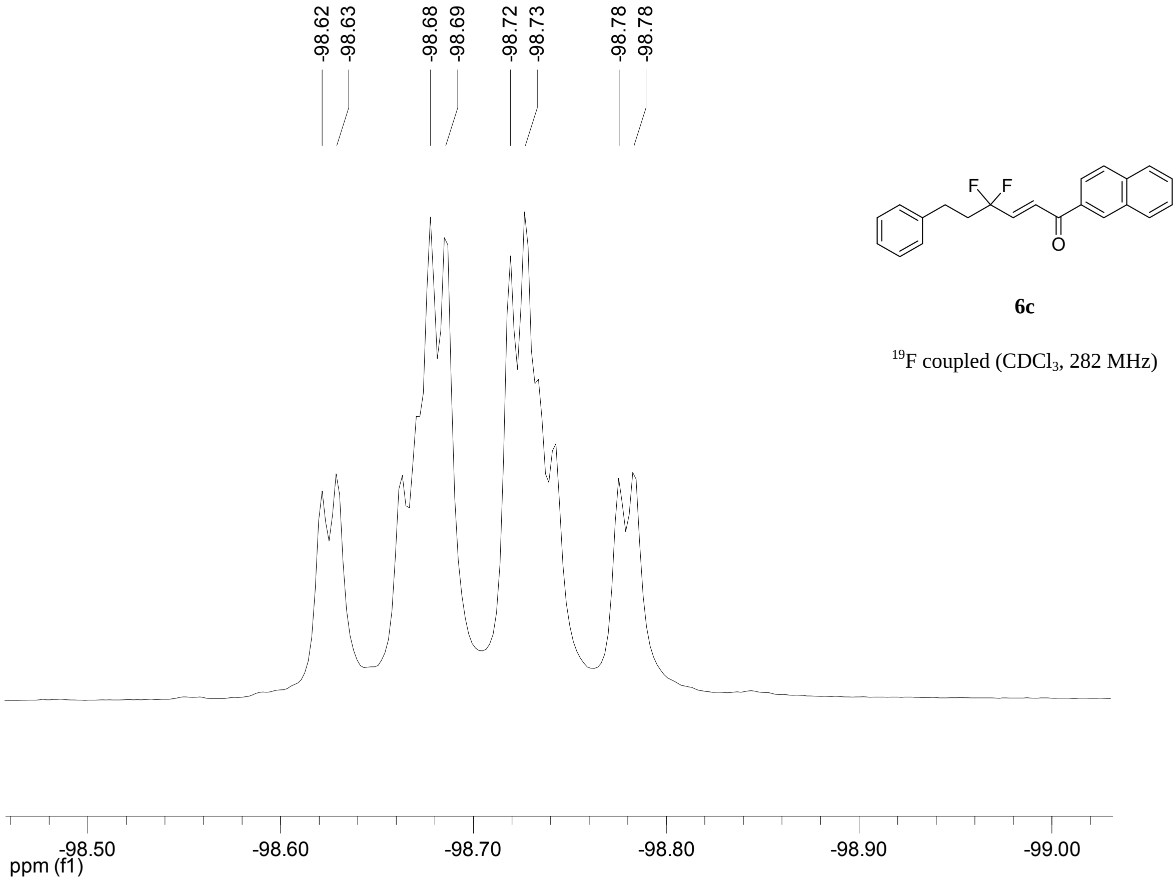

6c

^{19}F coupled ($CDCl_3$, 282 MHz)

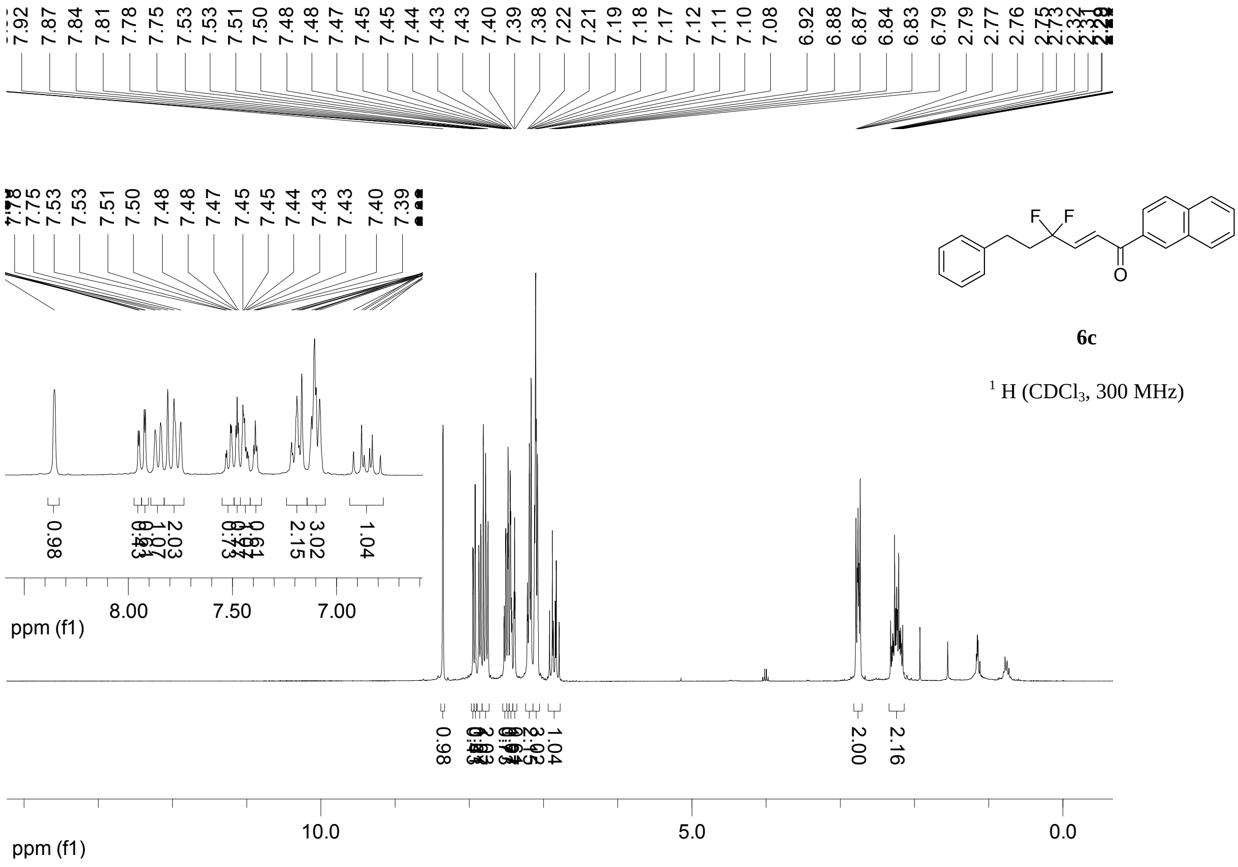
6c
1 H (CDCl3, 300 MHz)
ppm (f1)
10.0
5.0
0.0

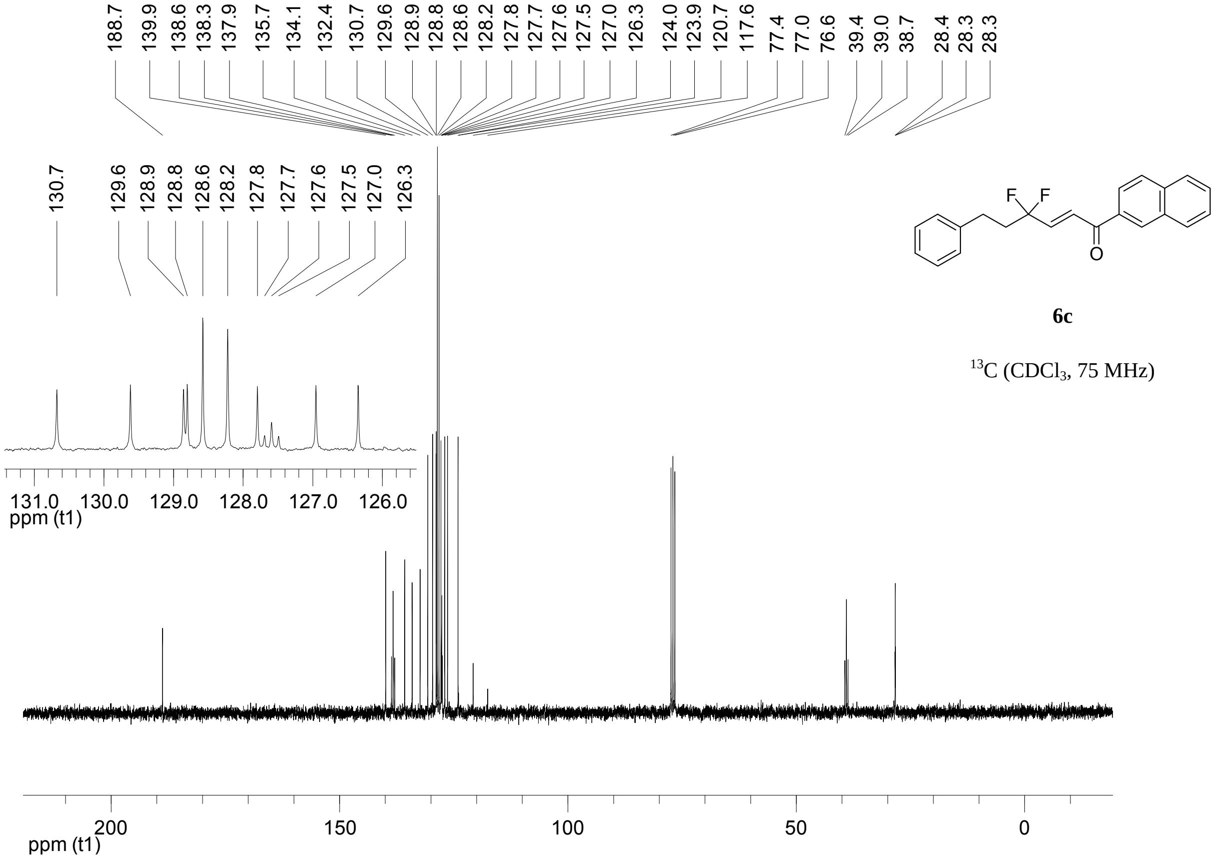
6c
13C (CDCl3, 75 MHz)
ppm (t1)
200
150
100
50
0

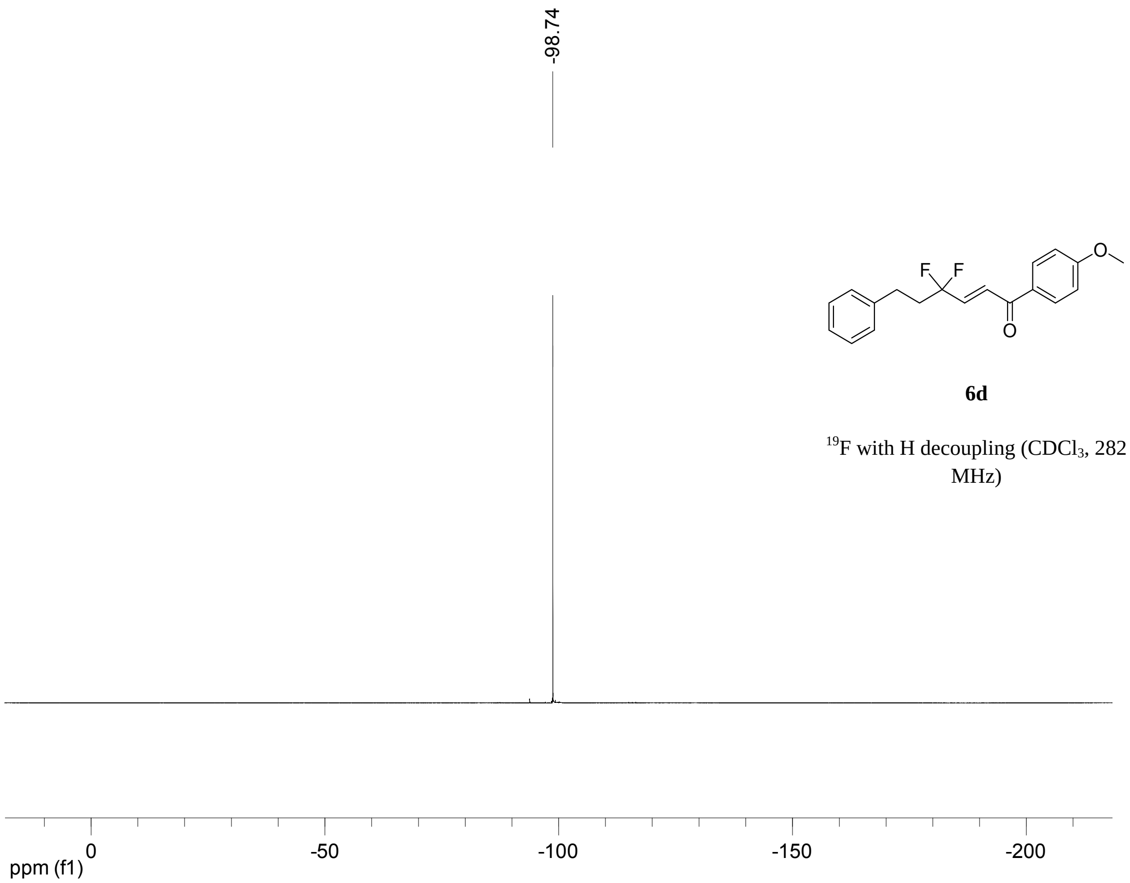

6d

^{19}F with H decoupling ($CDCl_3$, 282 MHz)

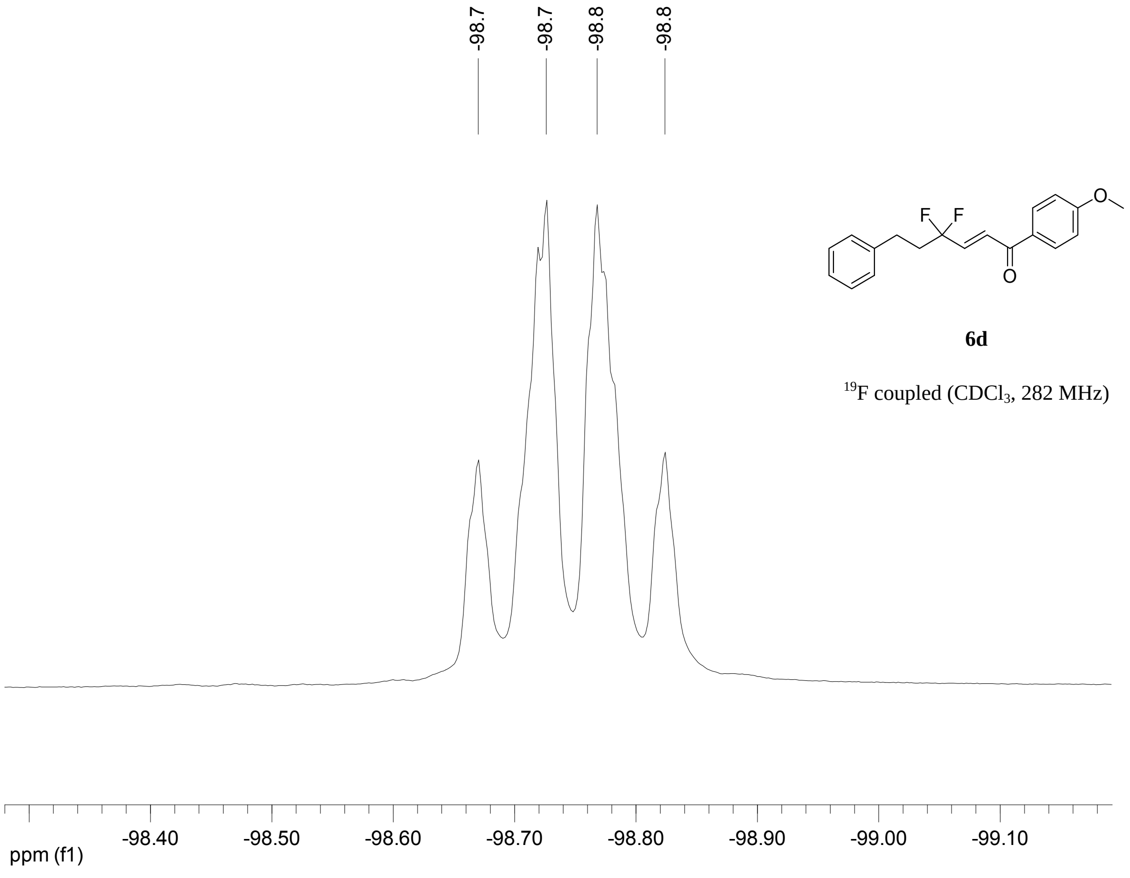

6d

^{19}F coupled ($CDCl_3$, 282 MHz)

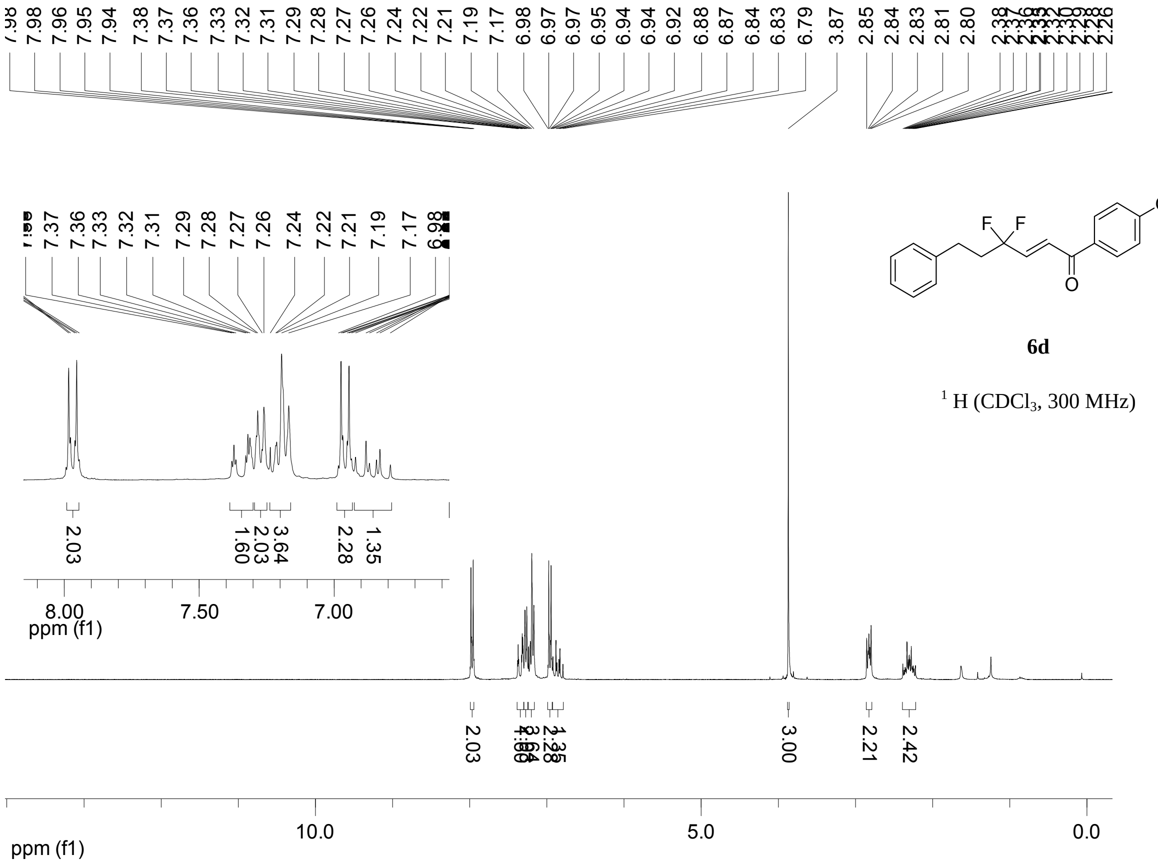
7.98 7.98 7.96 7.95 7.94 7.38 7.37 7.36 7.33 7.32 7.31 7.29 7.28 7.27 7.26 7.24 7.22 7.21 7.19 7.17 6.98 6.97 6.97 6.95 6.94 6.94 6.92 6.88 6.87 6.84 6.83 6.79 3.87 2.85 2.84 2.83 2.81 2.80
2.03
1.60
2.03
3.64
2.28
1.35
8.00
7.50
7.00
ppm (f1)
3.00
2.21
2.42
10.0
5.0
0.0
ppm (f1)
F F
O
6d
1 H (CDCl3, 300 MHz)

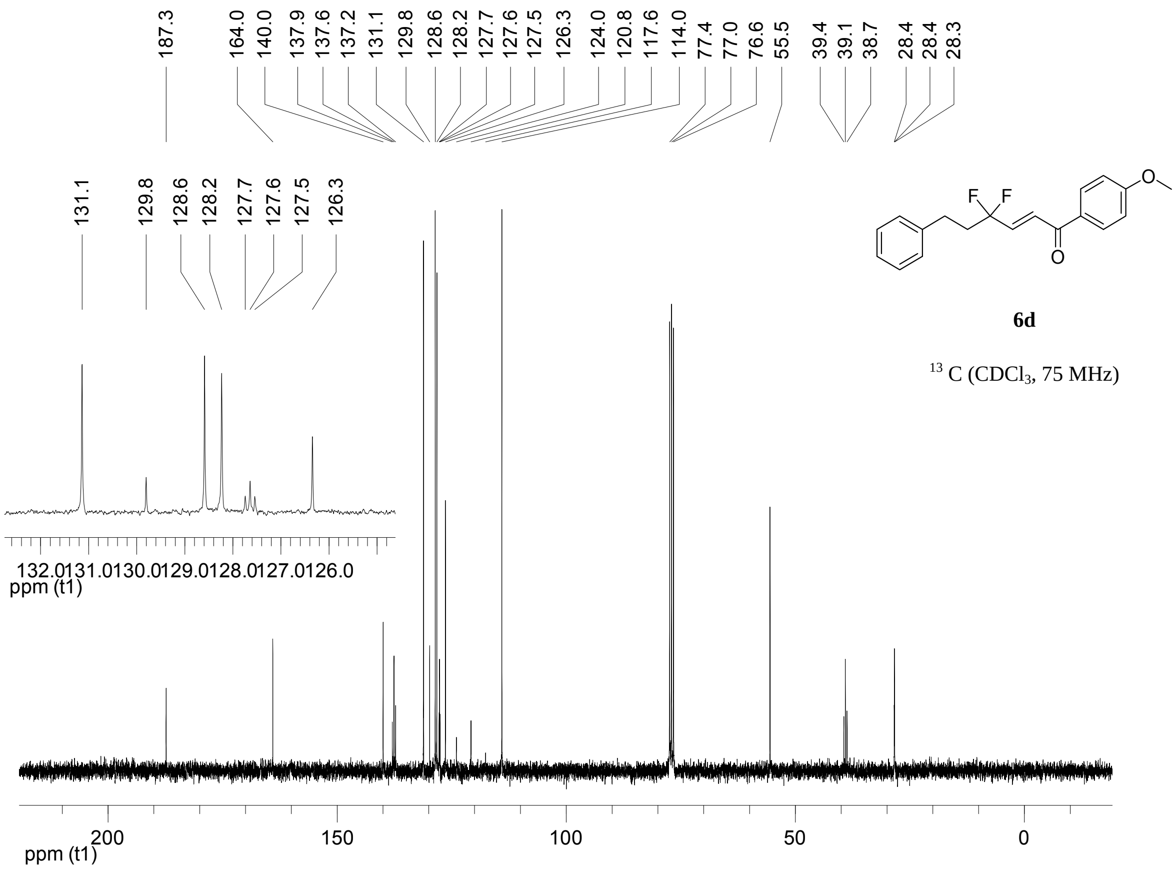
187.3 164.0 140.0 137.9 137.6 137.2 131.1 129.8 128.6 128.2 127.7 127.6 127.5 126.3 124.0 120.8 117.6 114.0 77.4 77.0 76.6 55.5 39.4 39.1 38.7 28.4 28.4 28.3
131.1 129.8 128.6 128.2 127.7 127.6 127.5 126.3
132.0 131.0 130.0 129.0 128.0 127.0 126.0
ppm (t1)
200
150
100
50
0
ppm (t1)
F F
O
6d
13 C (CDCl3, 75 MHz)

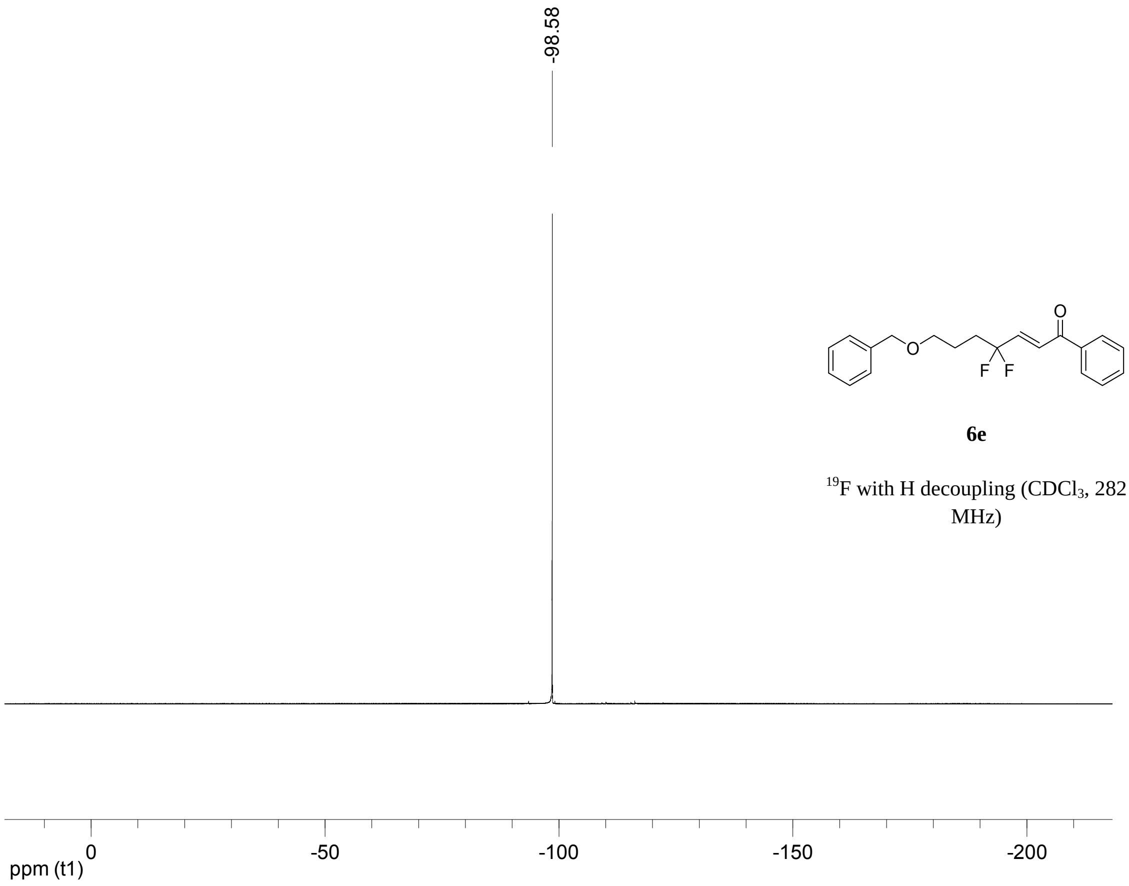

6e

^{19}F with H decoupling ($CDCl_3$, 282 MHz)

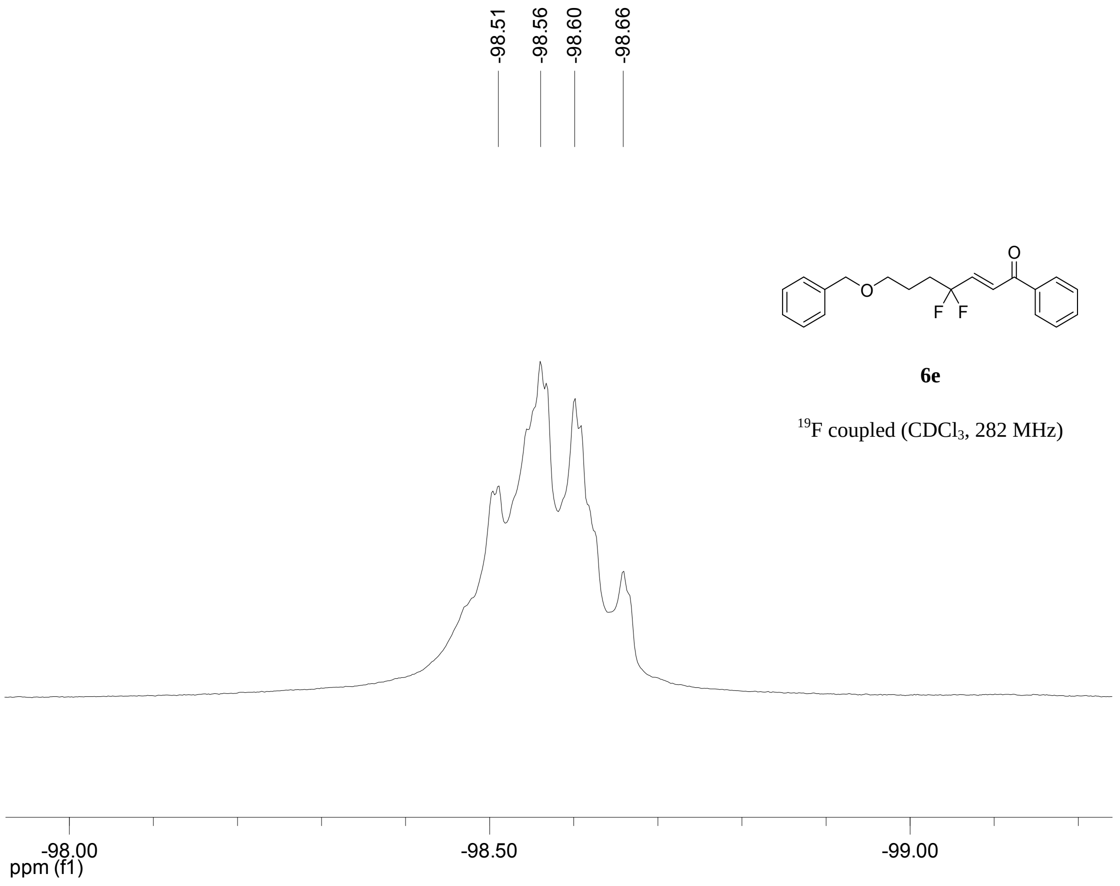

6e

^{19}F coupled ($CDCl_3$, 282 MHz)

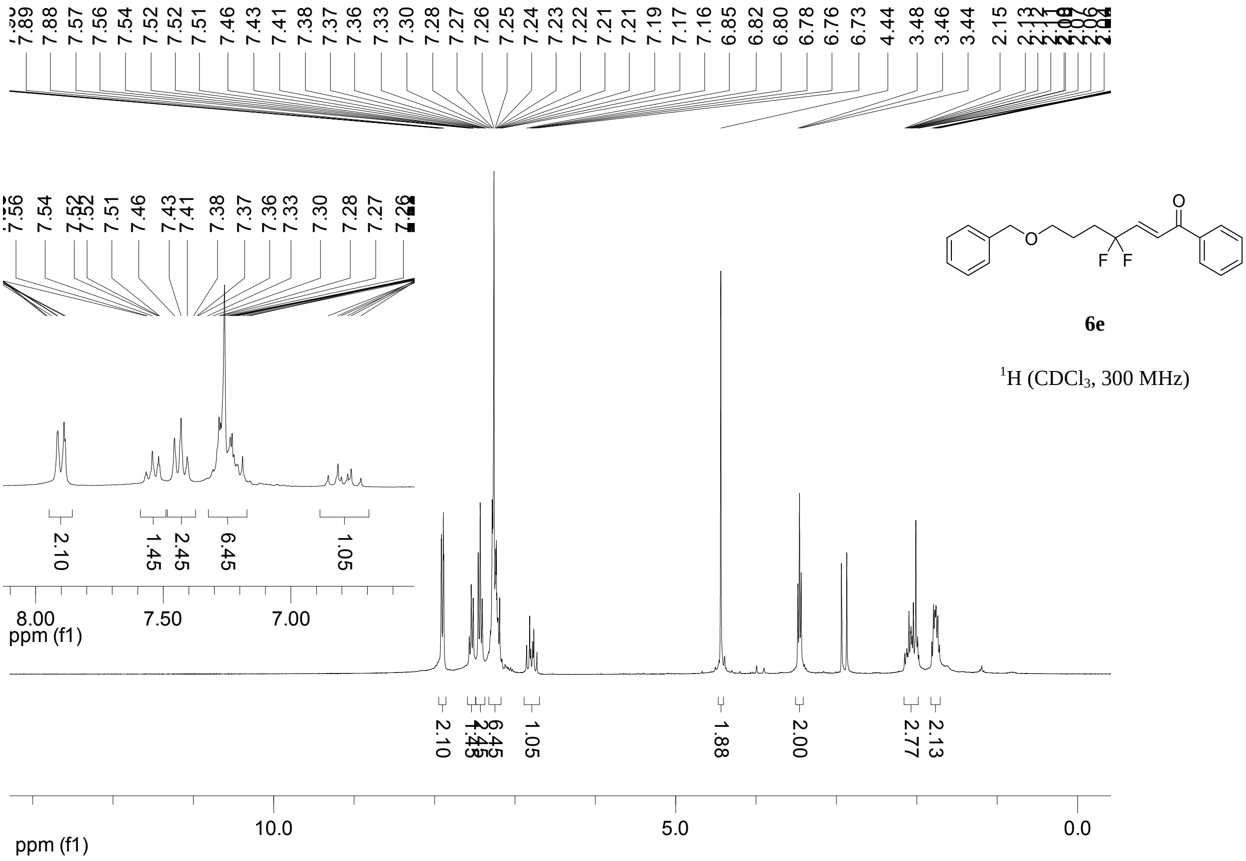

6e

^{1}H ($CDCl_3$, 300 MHz)

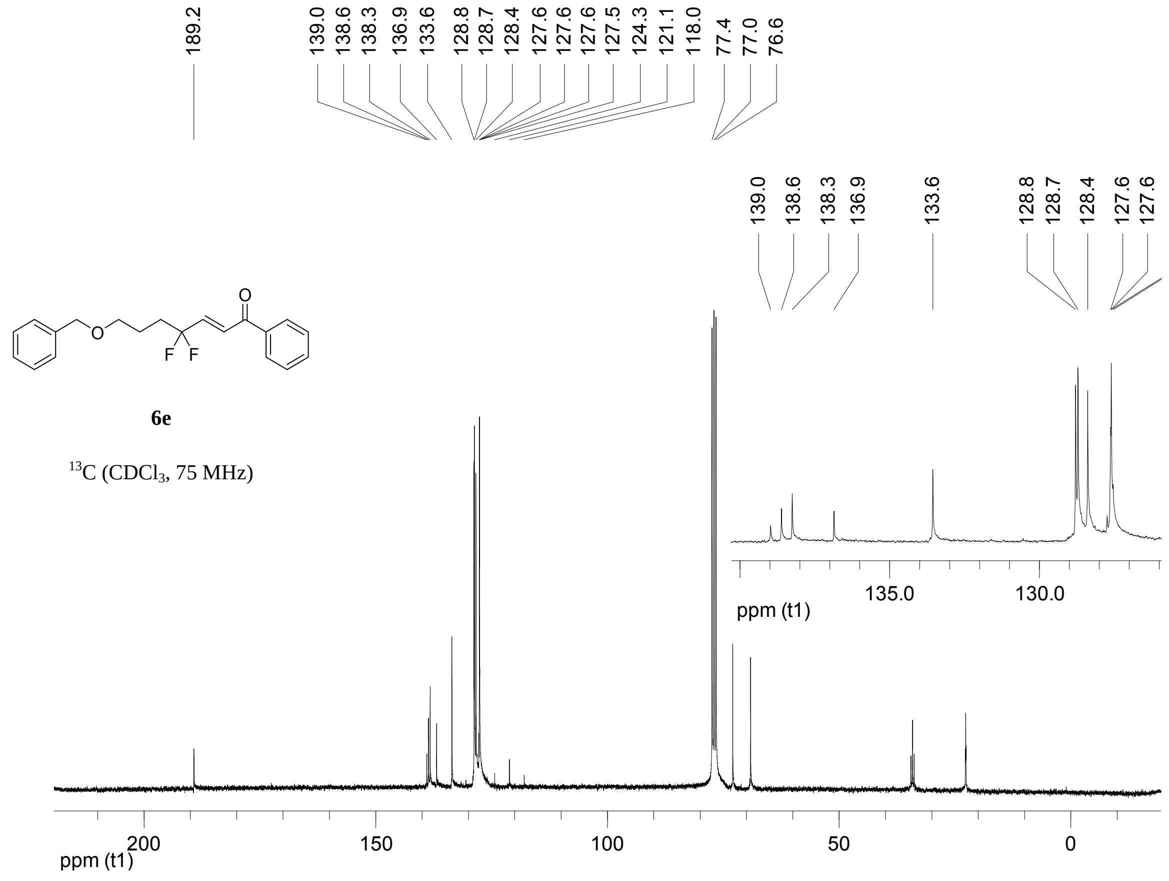

6e

^{13}C ($CDCl_3$, 75 MHz)

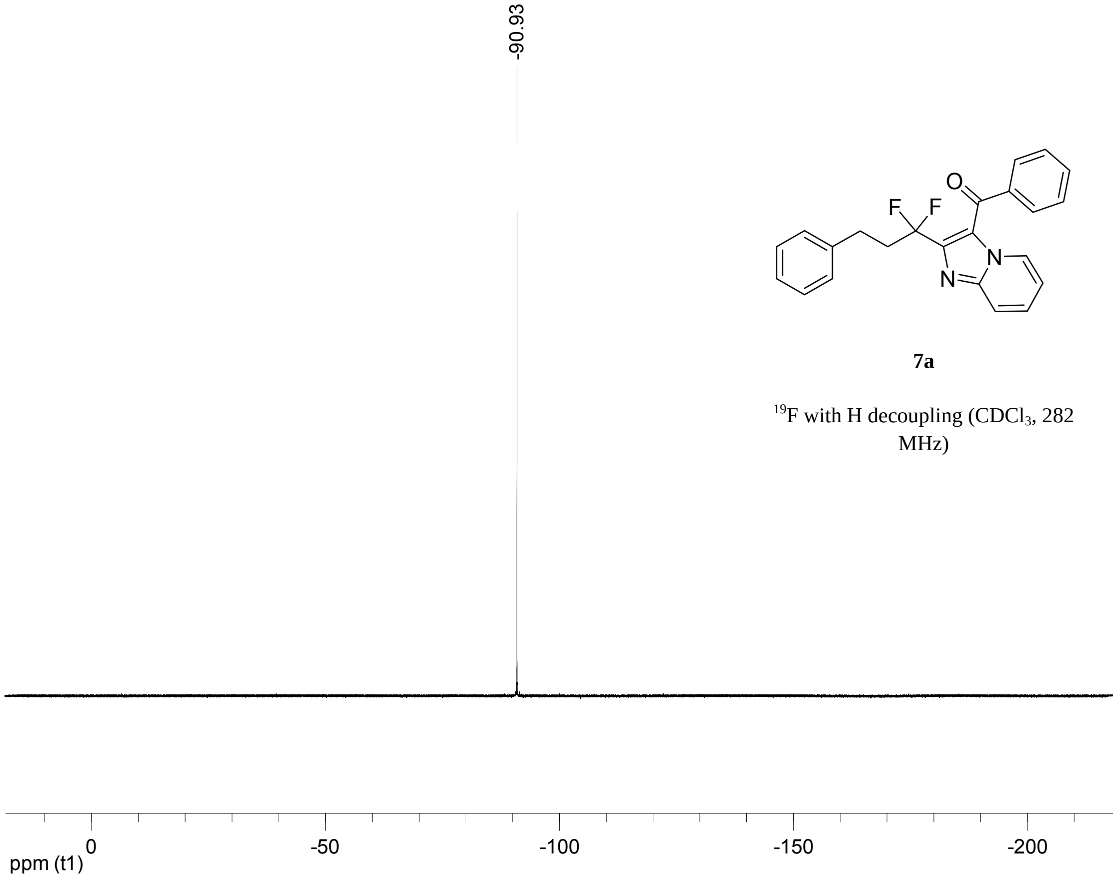

7a

^{19}F with H decoupling ($CDCl_3$, 282 MHz)

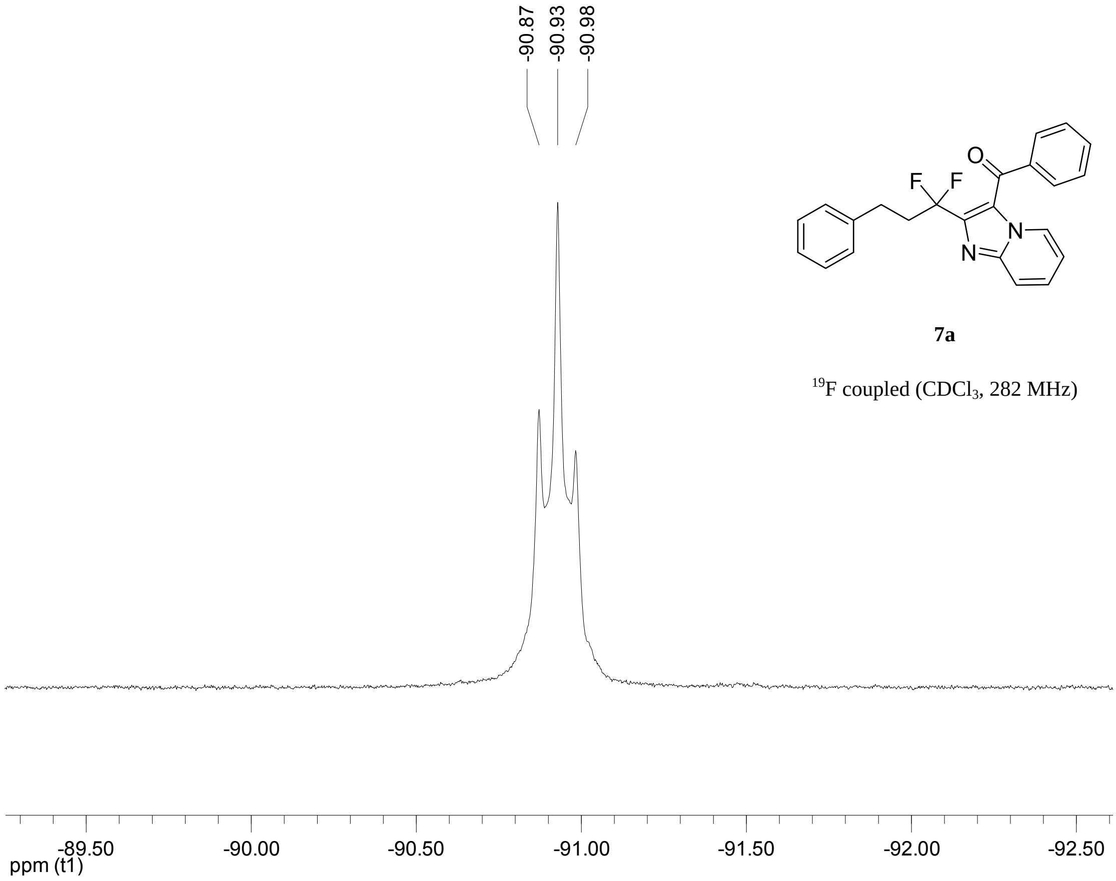

7a

^{19}F coupled ($CDCl_3$, 282 MHz)

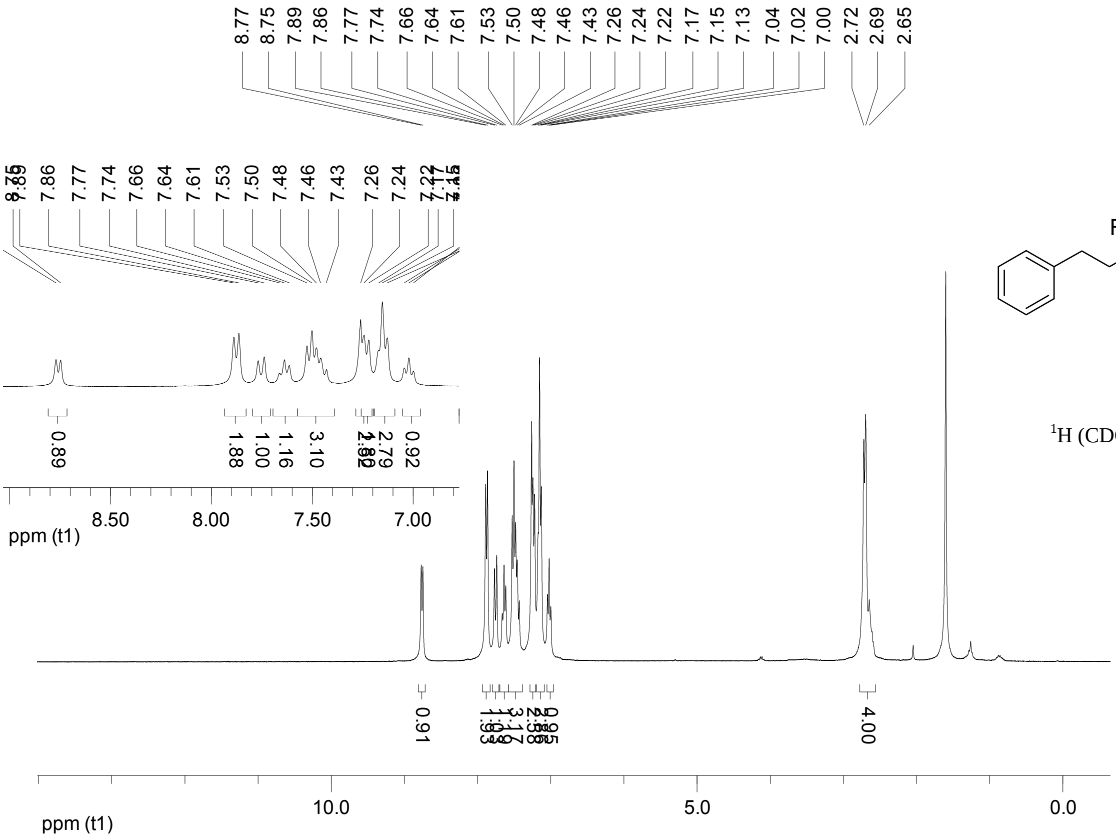
8.77 8.75 7.89 7.86 7.77 7.74 7.66 7.64 7.61 7.53 7.50 7.48 7.46 7.43 7.26 7.24 7.22 7.17 7.15 7.13 7.04 7.02 7.00 2.72 2.69 2.65
0.89 1.88 1.00 1.16 3.10 2.79 0.92
8.50 8.00 7.50 7.00
ppm (t1)
7a
^{1}H ($CDCl_3$, 300 MHz)
0.91 4.00
10.0 5.0 0.0
ppm (t1)

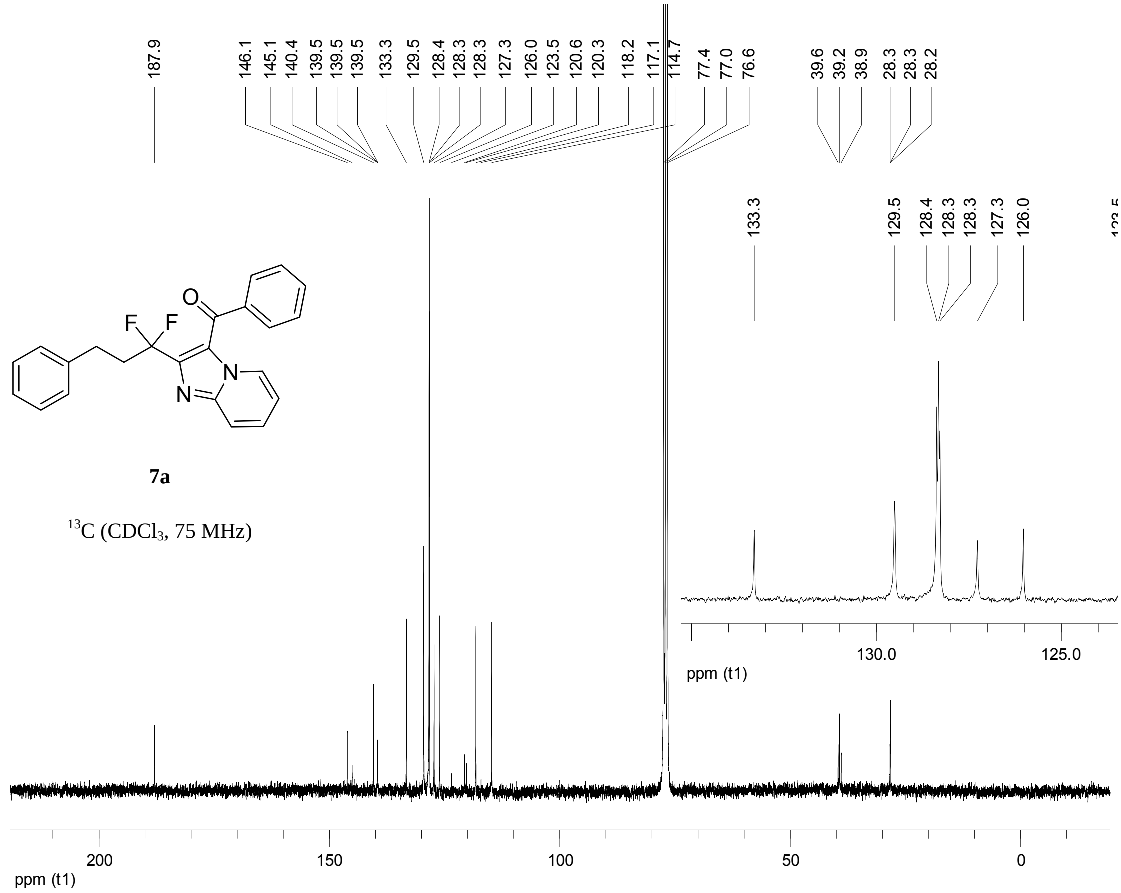
187.9 146.1 145.1 140.4 139.5 139.5 139.5 133.3 129.5 128.4 128.3 128.3 127.3 126.0 123.5 120.6 120.3 118.2 117.1 114.7 77.4 77.0 76.6 39.6 39.2 38.9 28.3 28.3 28.2
133.3 129.5 128.4 128.3 128.3 127.3 126.0
130.0 125.0
ppm (t1)
7a
^{13}C ($CDCl_3$, 75 MHz)
200 150 100 50 0
ppm (t1)

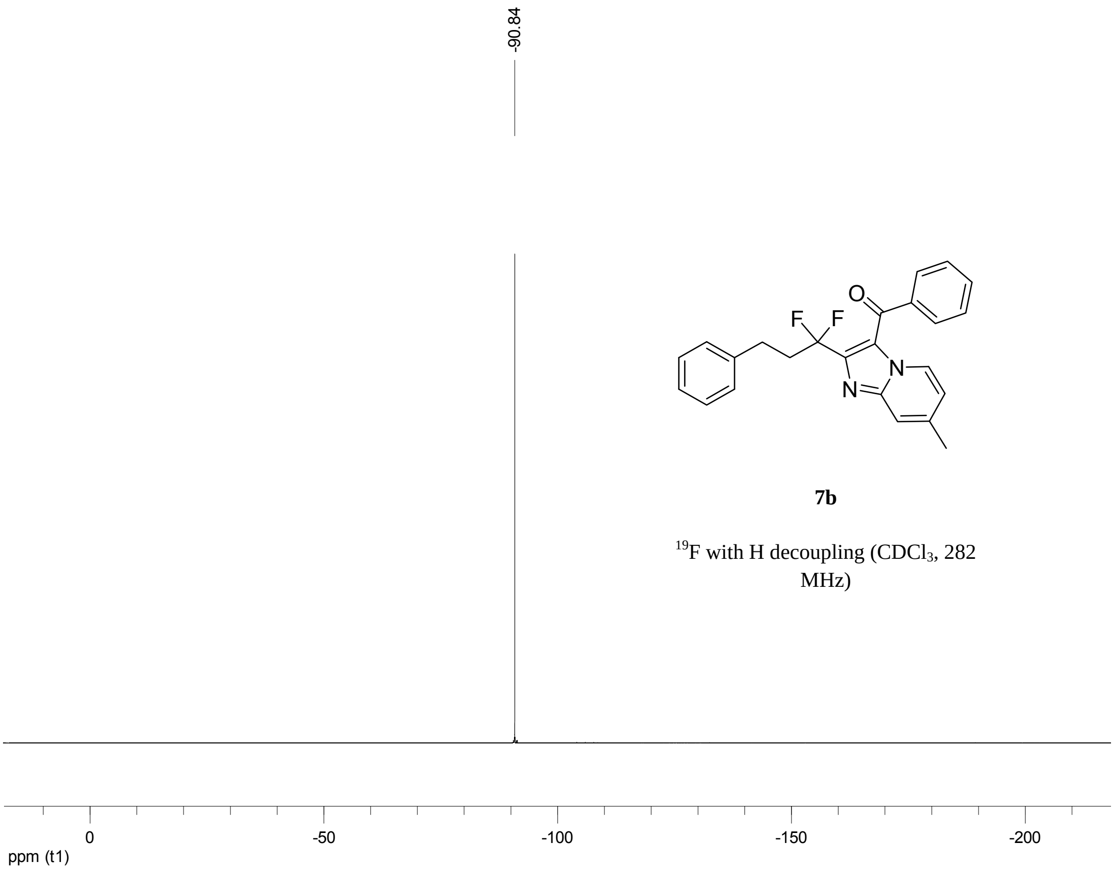

7b

^{19}F with H decoupling ($CDCl_3$, 282 MHz)

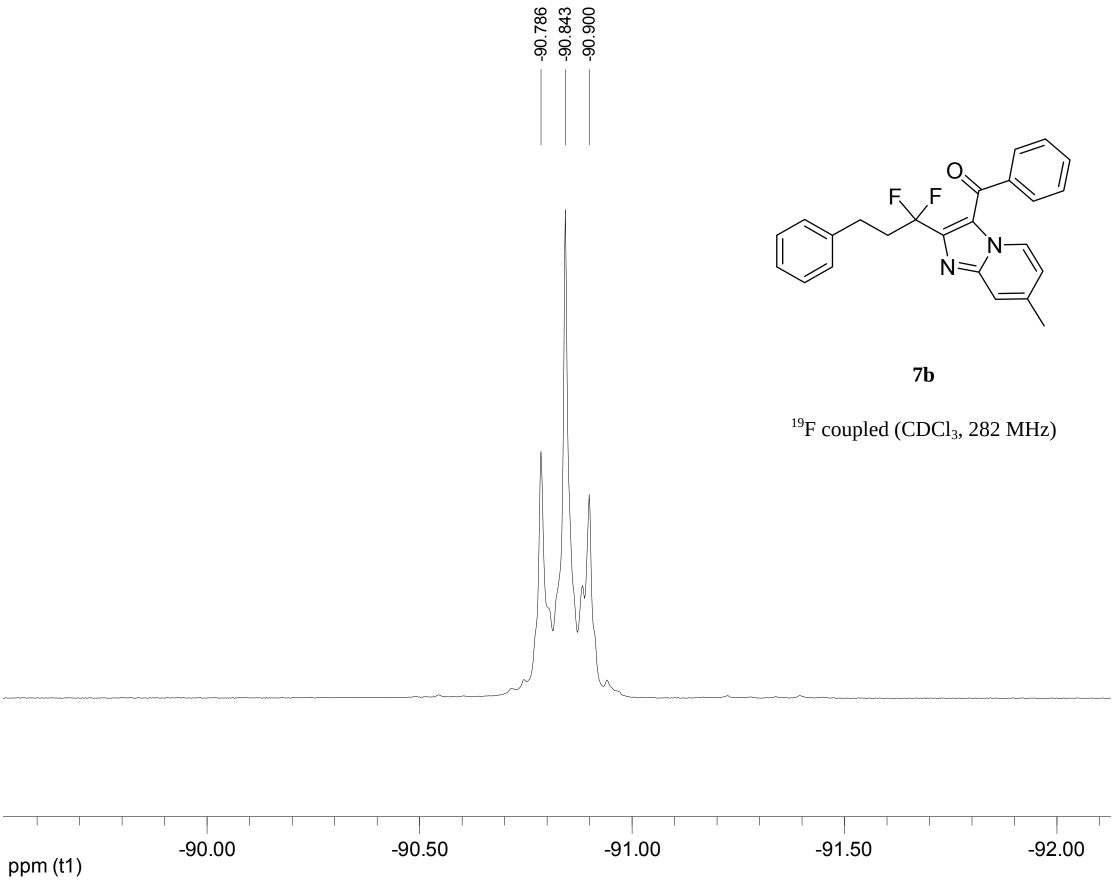

7b

^{19}F coupled ($CDCl_3$, 282 MHz)

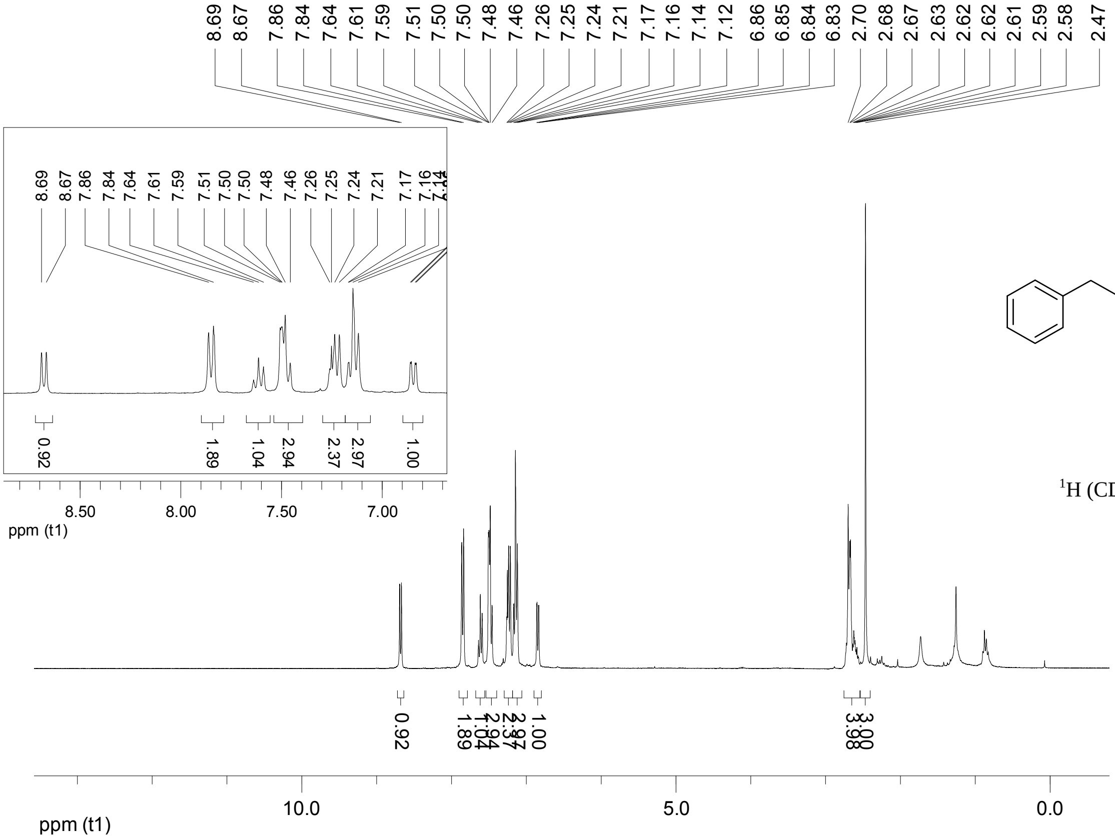

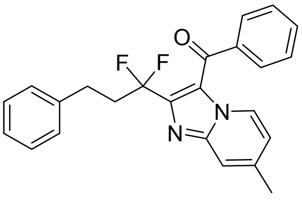

7b

^{1}H ($CDCl_3$, 300 MHz)

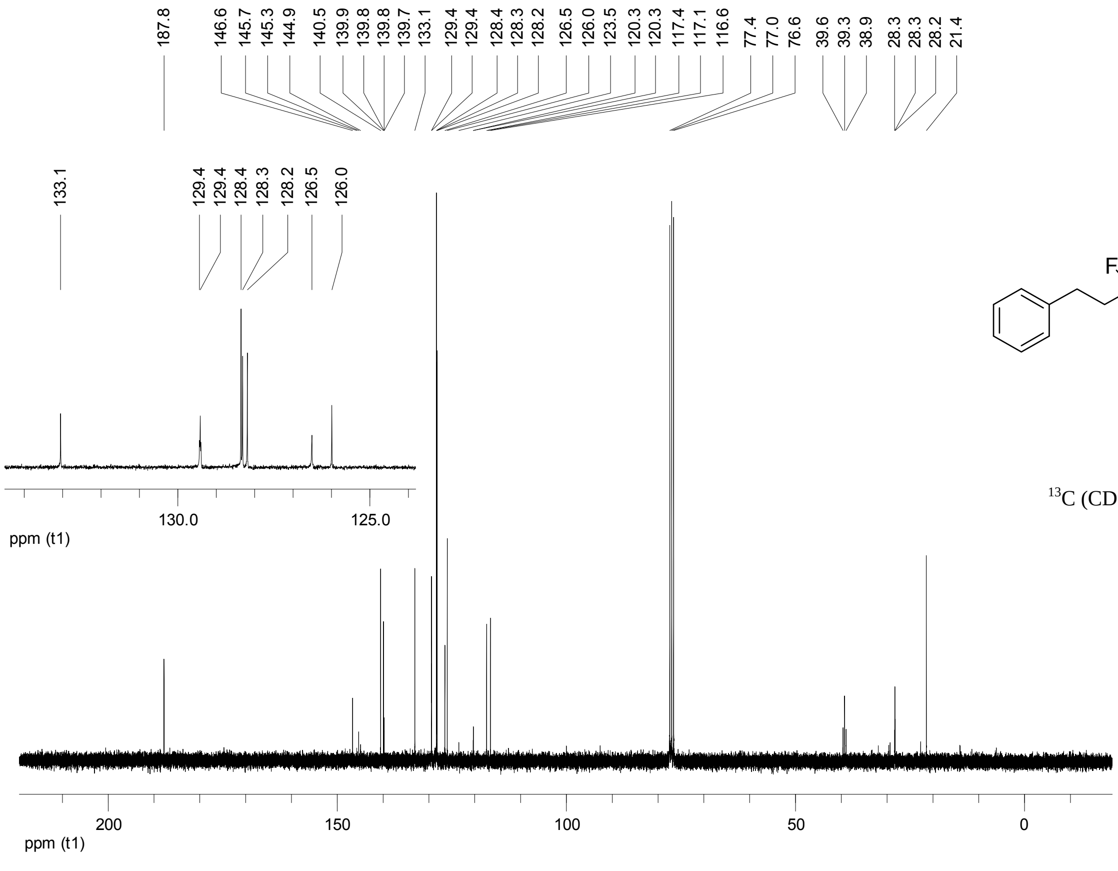

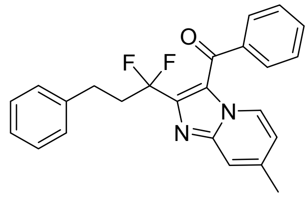

7b

^{13}C ($CDCl_3$, 75 MHz)

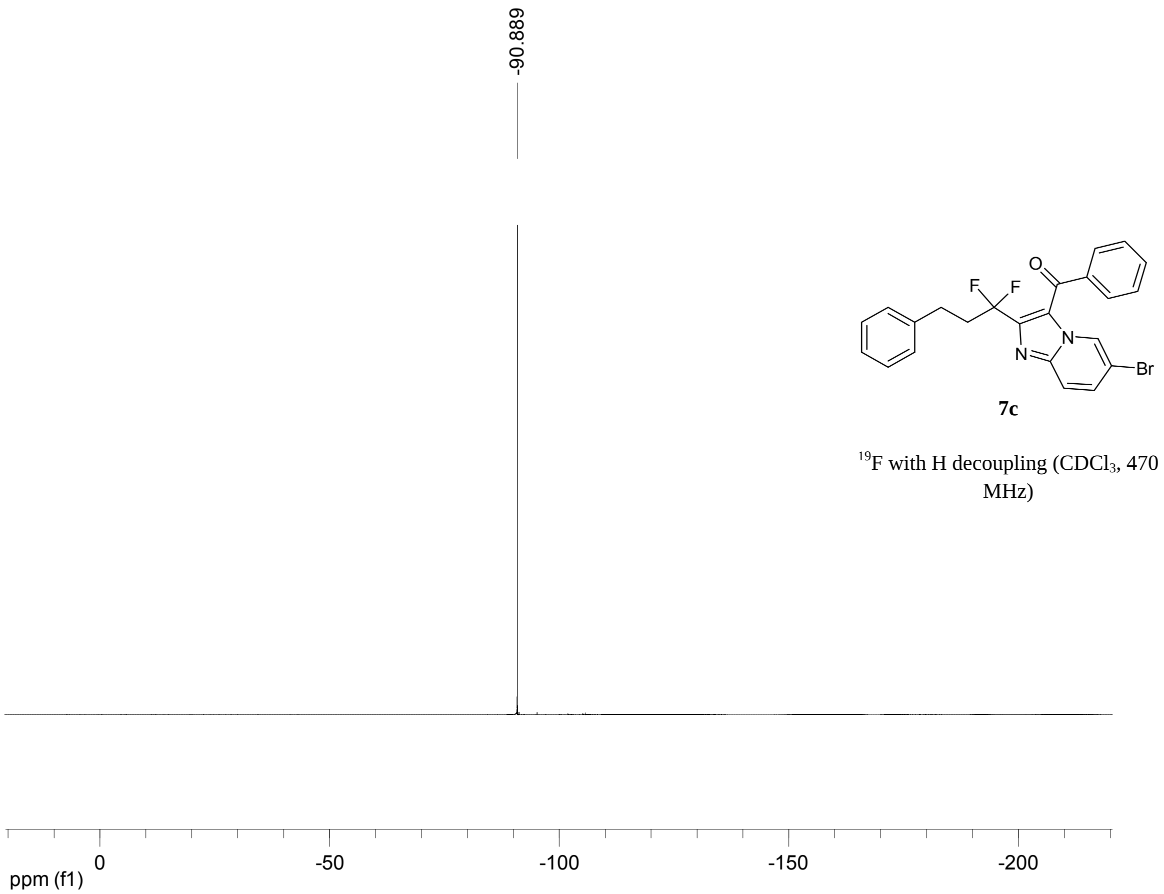
-90.889
7c
^{19}F with H decoupling ($CDCl_3$, 470 MHz)
ppm (f1)
0
-50
-100
-150
-200

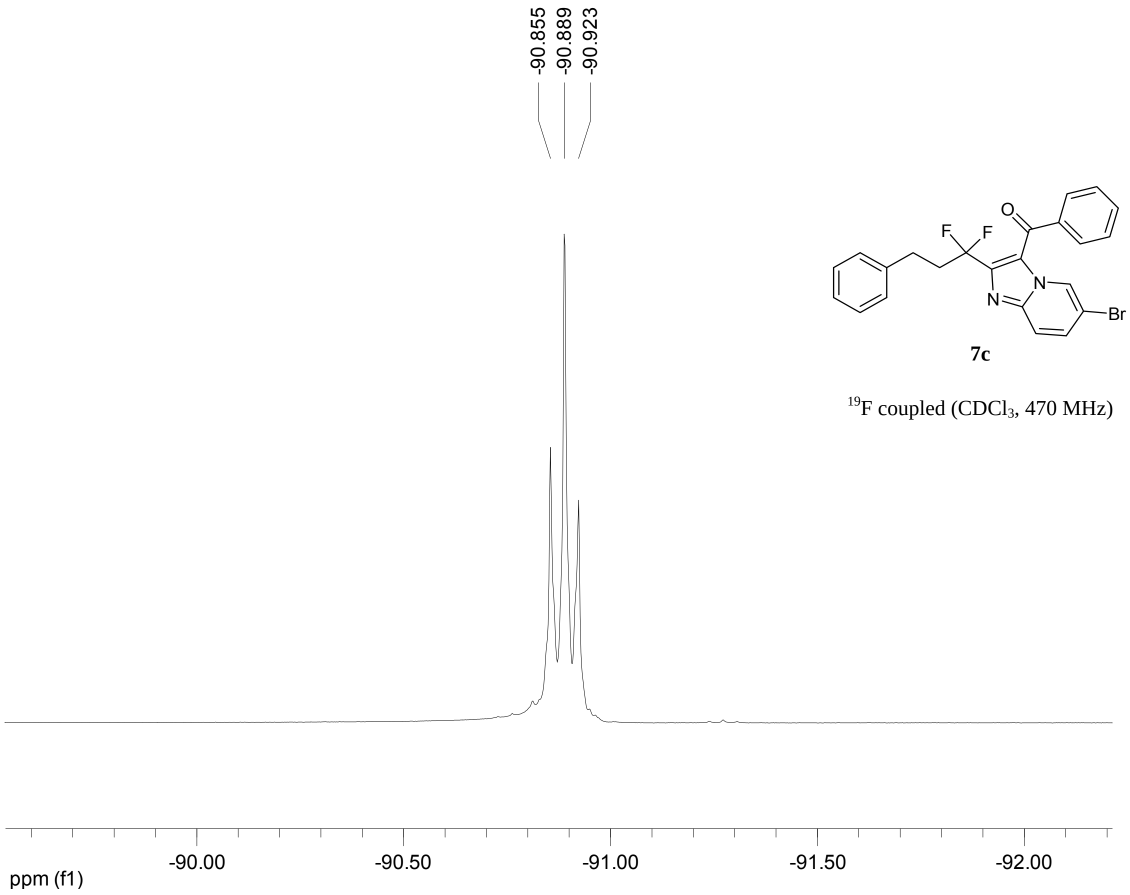
-90.855
-90.889
-90.923
7c
^{19}F coupled ($CDCl_3$, 470 MHz)
ppm (f1)
-90.00
-90.50
-91.00
-91.50
-92.00

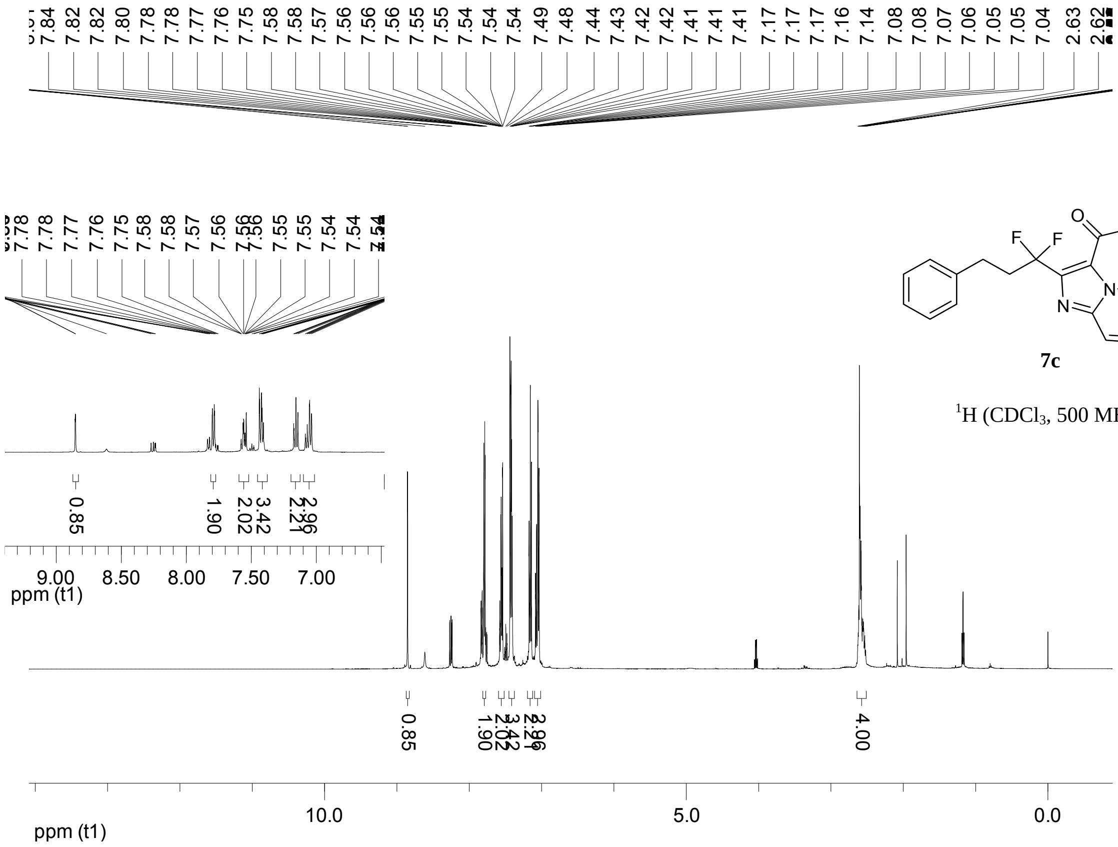

7c

^{1}H ($CDCl_3$, 500 MHz)

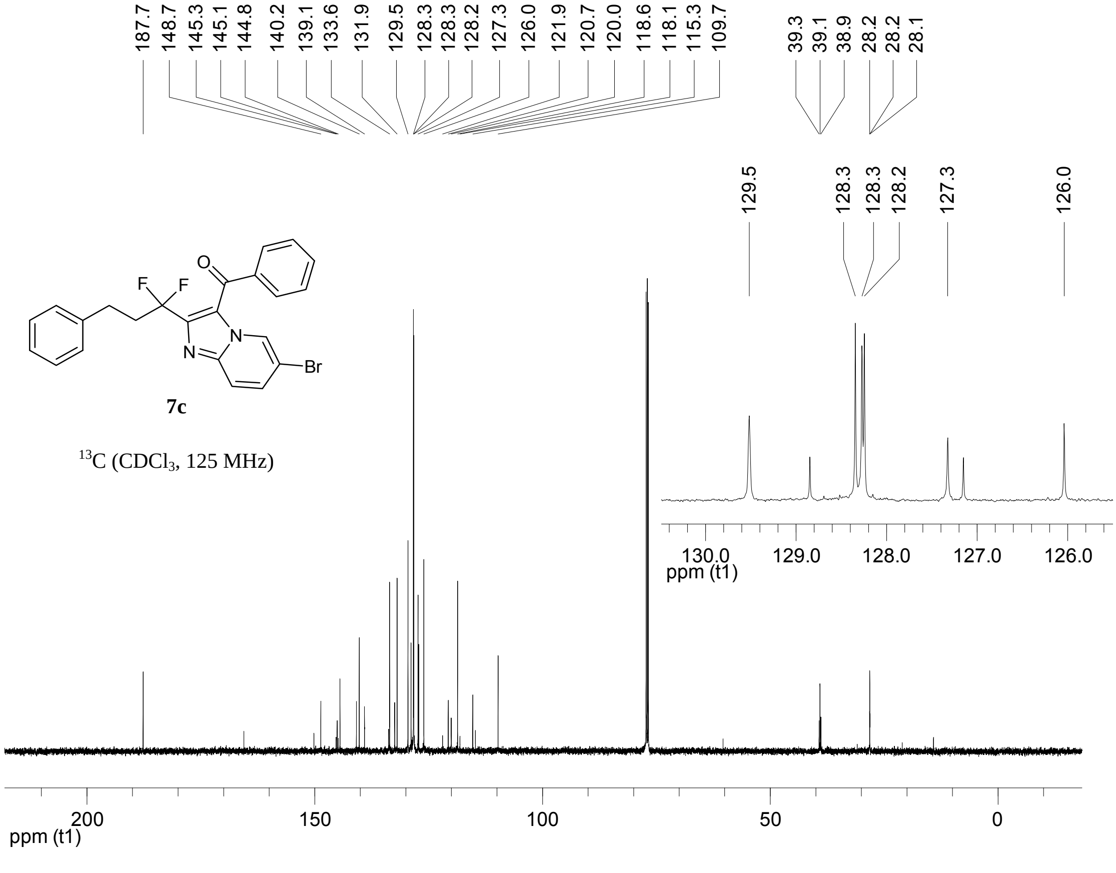

7c

^{13}C ($CDCl_3$, 125 MHz)

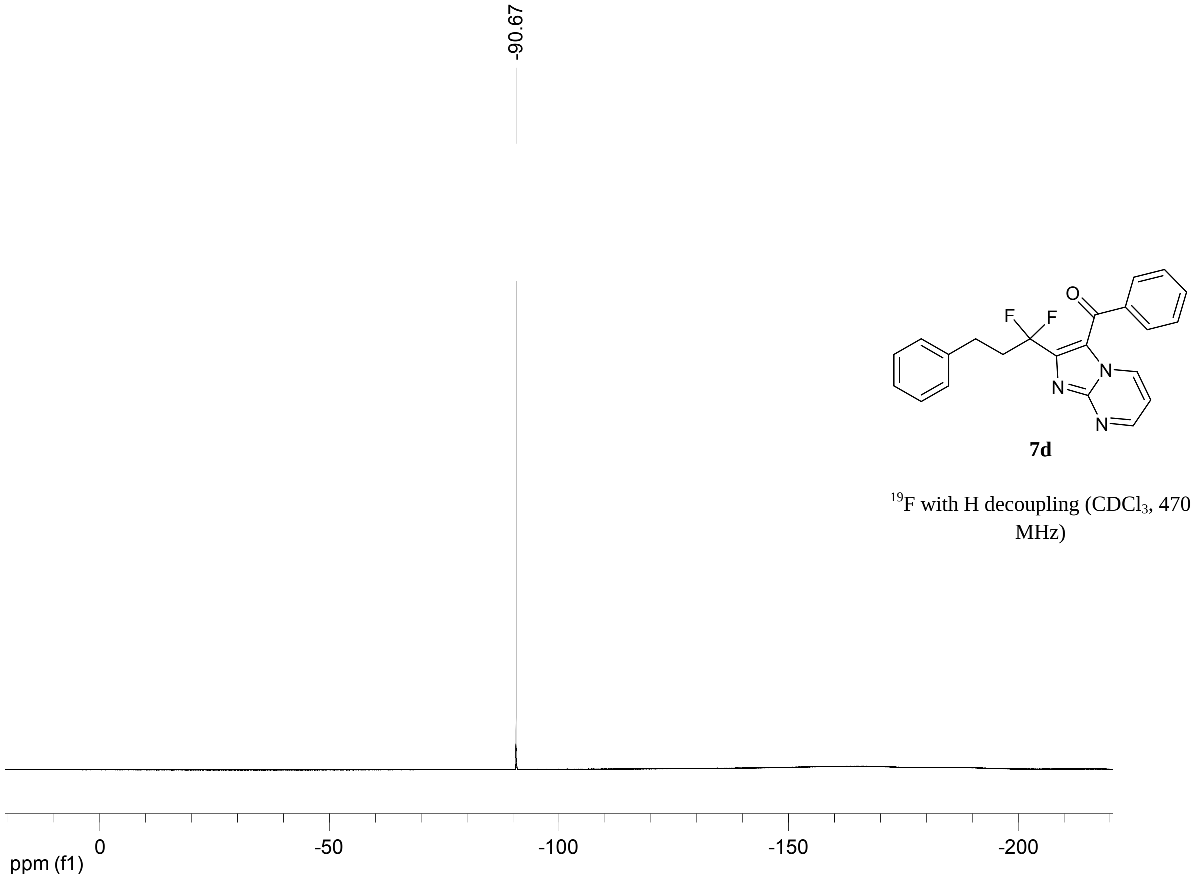

7d

^{19}F with H decoupling ($CDCl_3$, 470 MHz)

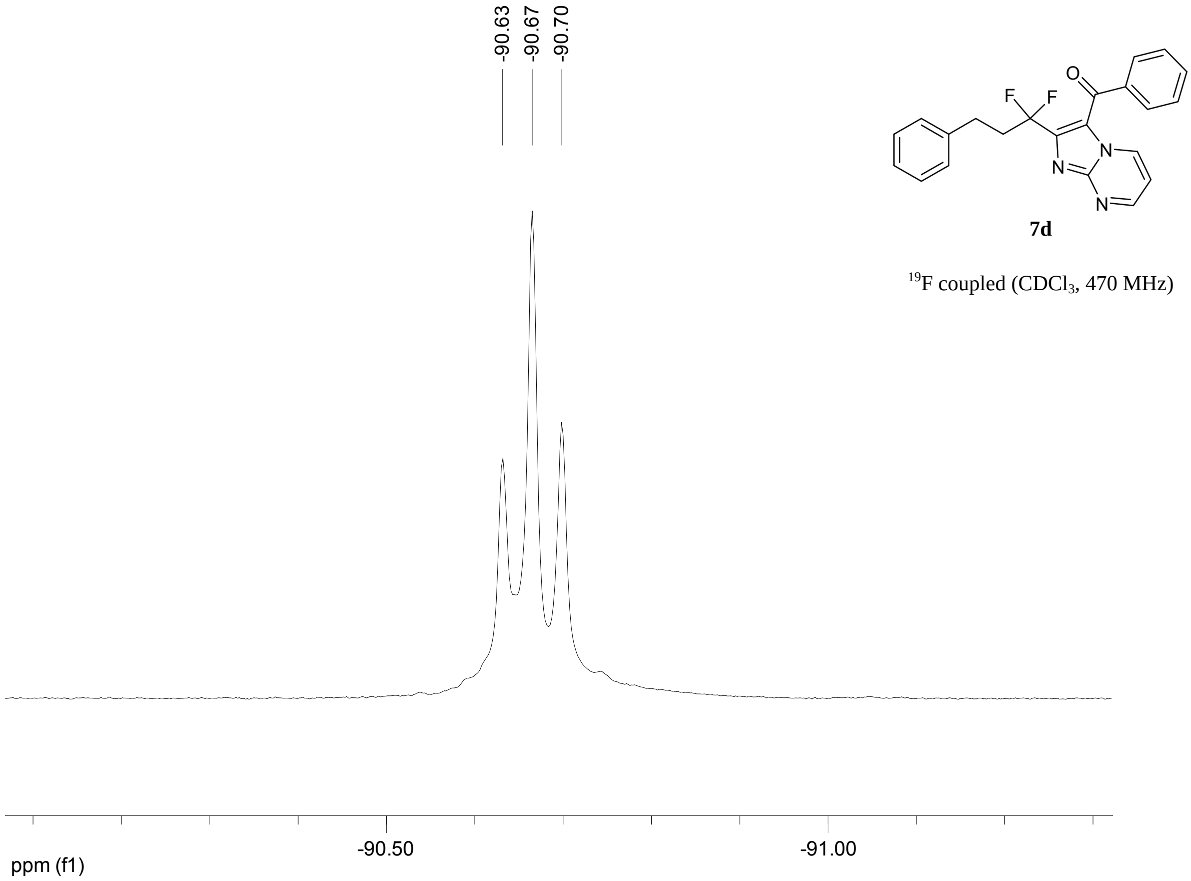

7d

^{19}F coupled ($CDCl_3$, 470 MHz)

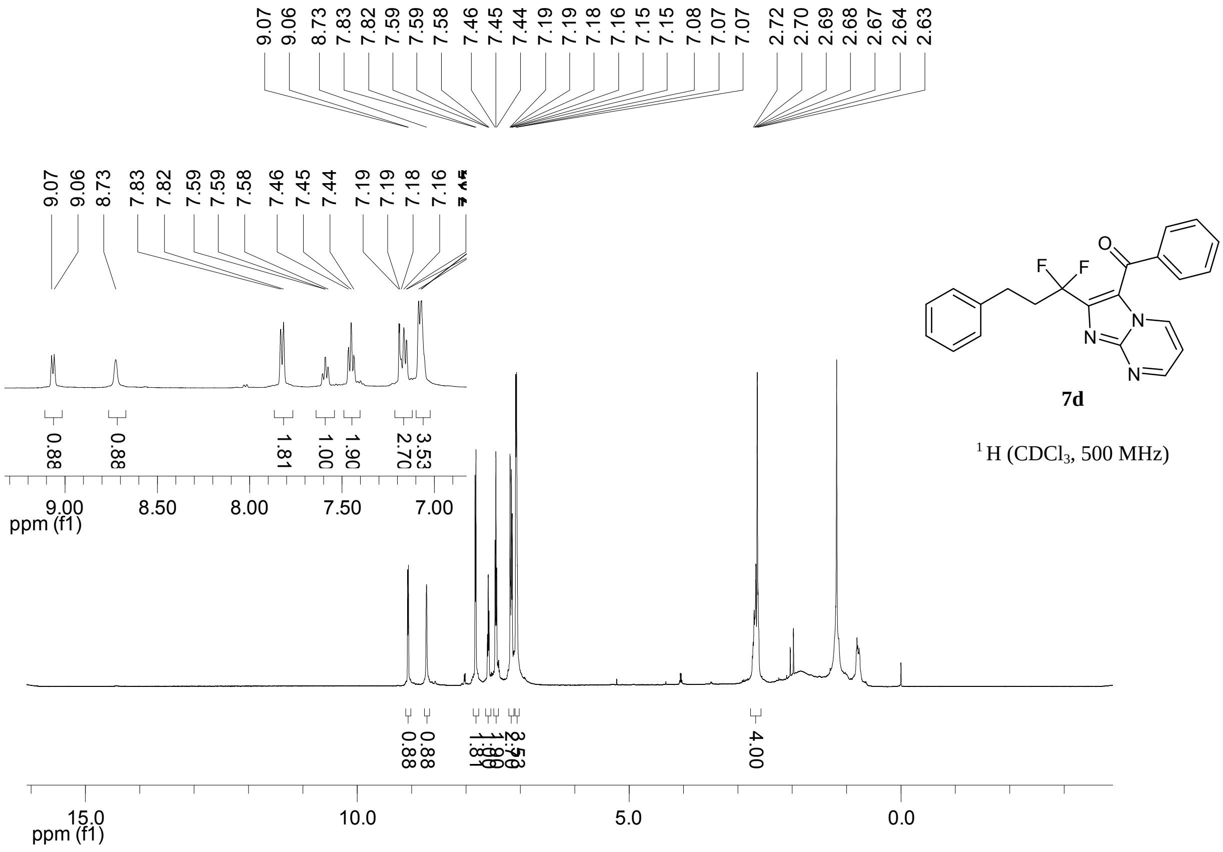

7d

^{1}H (CDCl$_3$, 500 MHz)

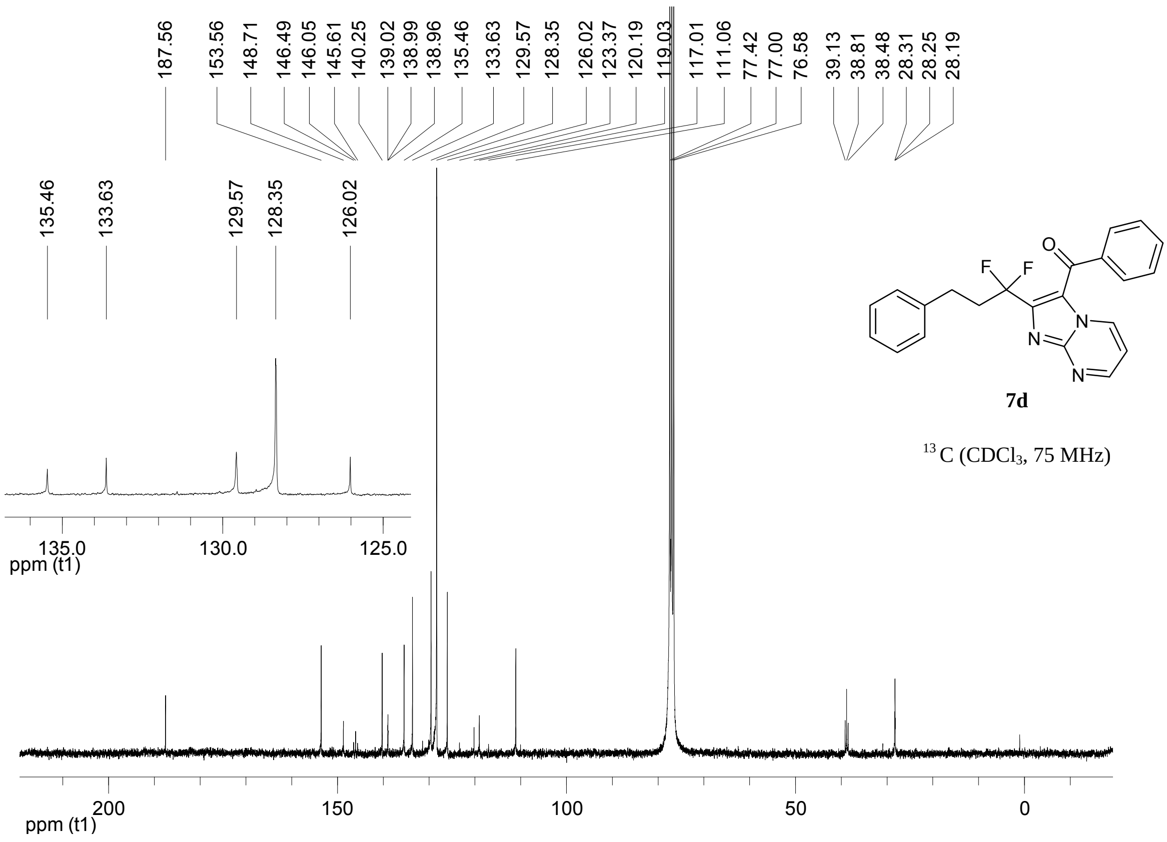

7d

^{13}C (CDCl$_3$, 75 MHz)

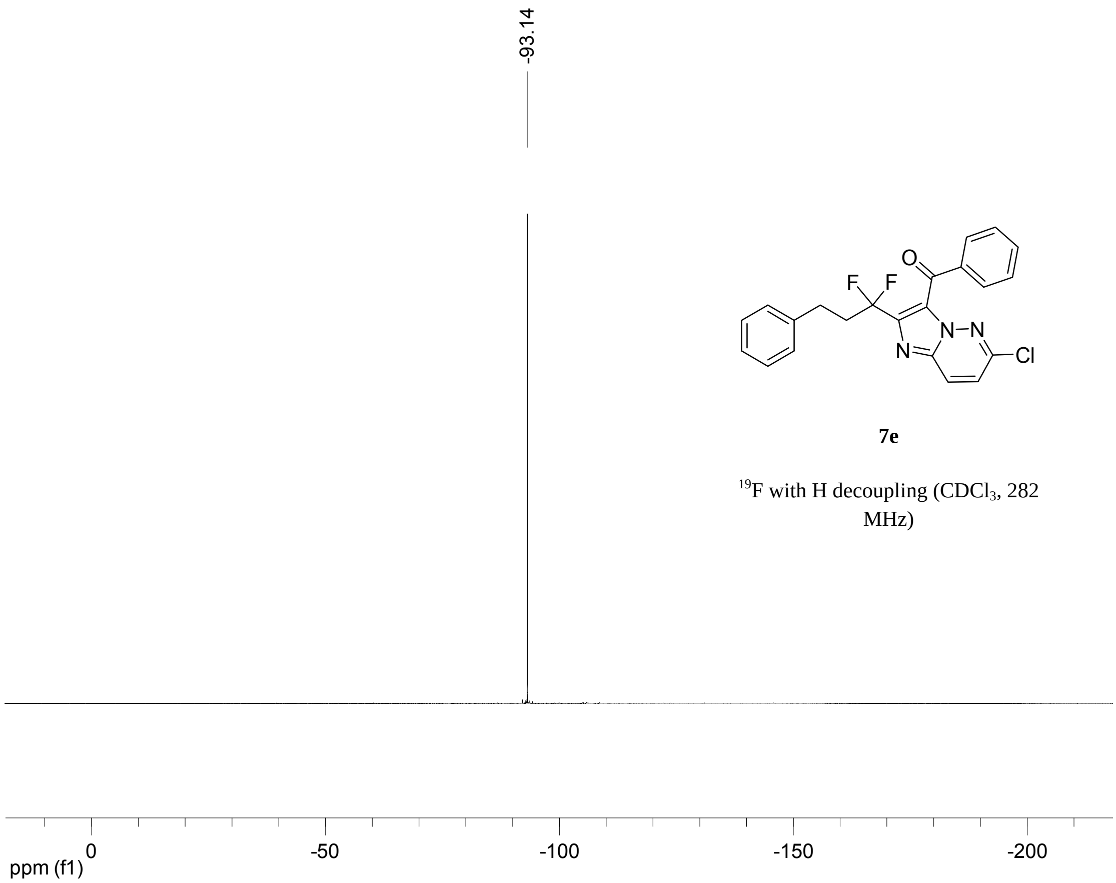

7e

^{19}F with H decoupling ($CDCl_3$, 282 MHz)

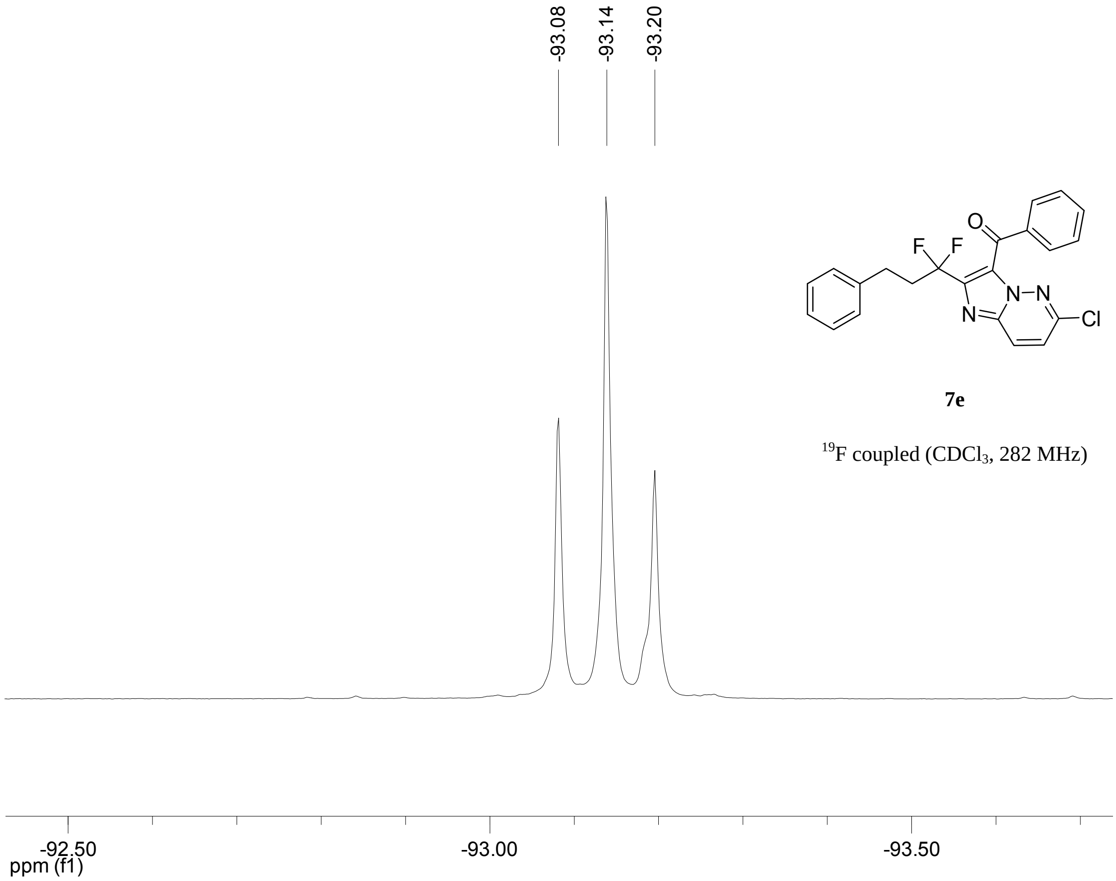

7e

^{19}F coupled ($CDCl_3$, 282 MHz)

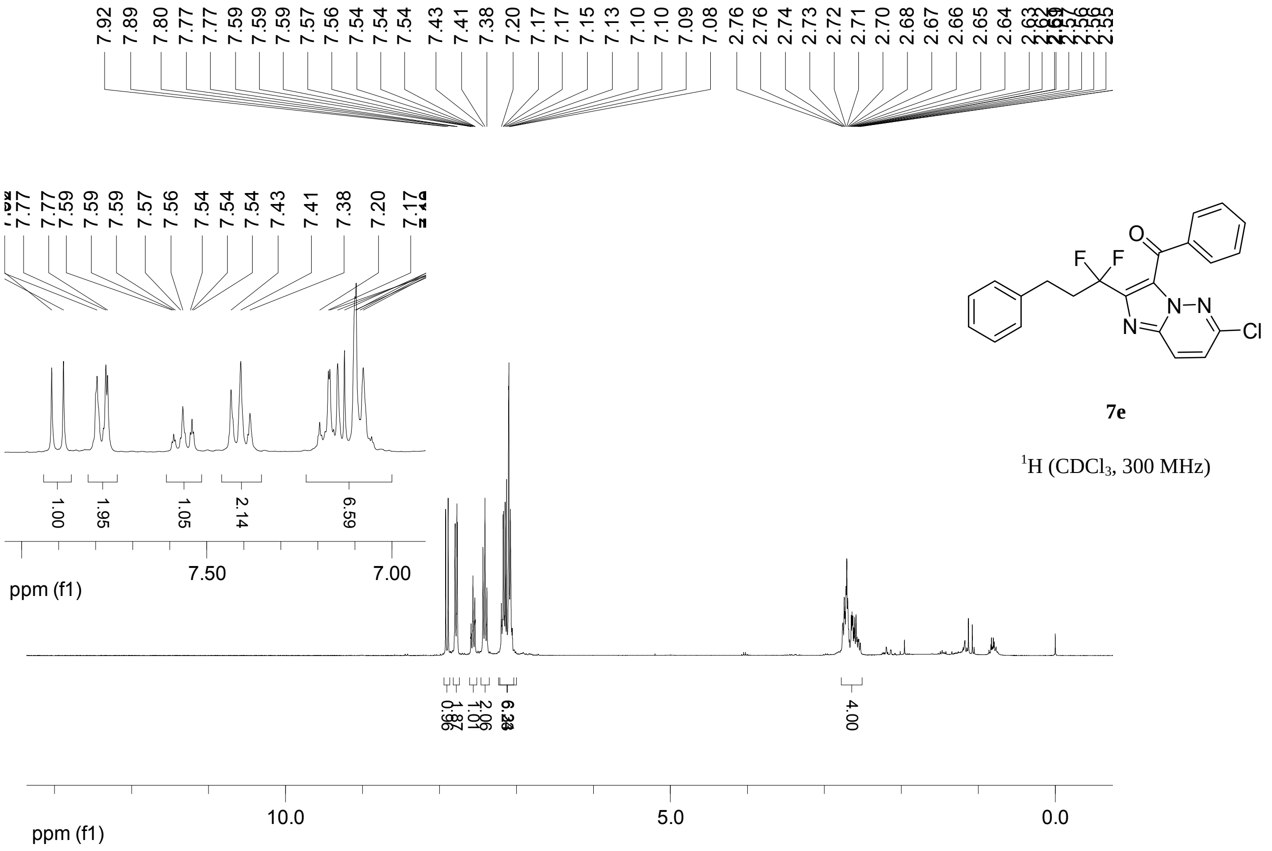

7e

^{1}H ($CDCl_3$, 300 MHz)

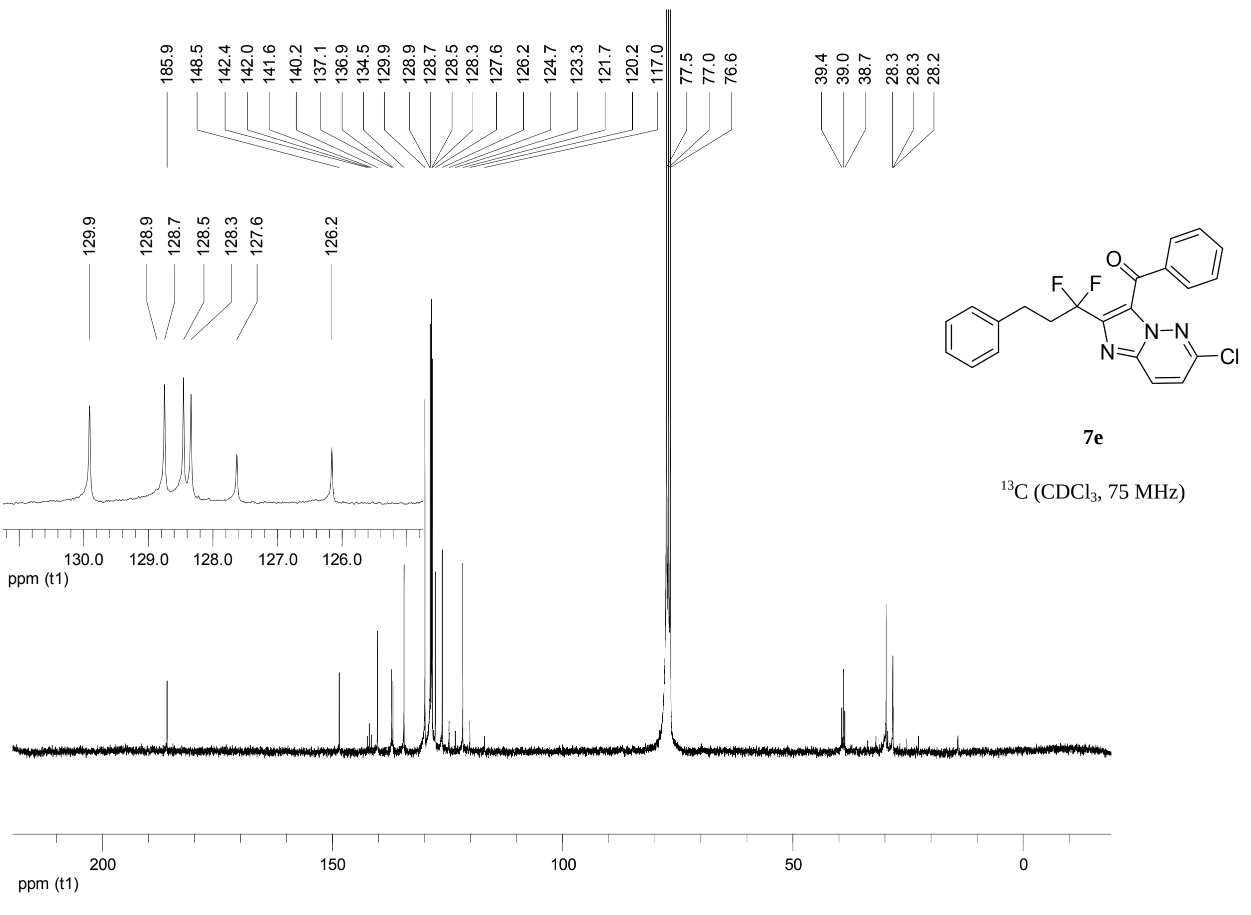

7e

^{13}C ($CDCl_3$, 75 MHz)

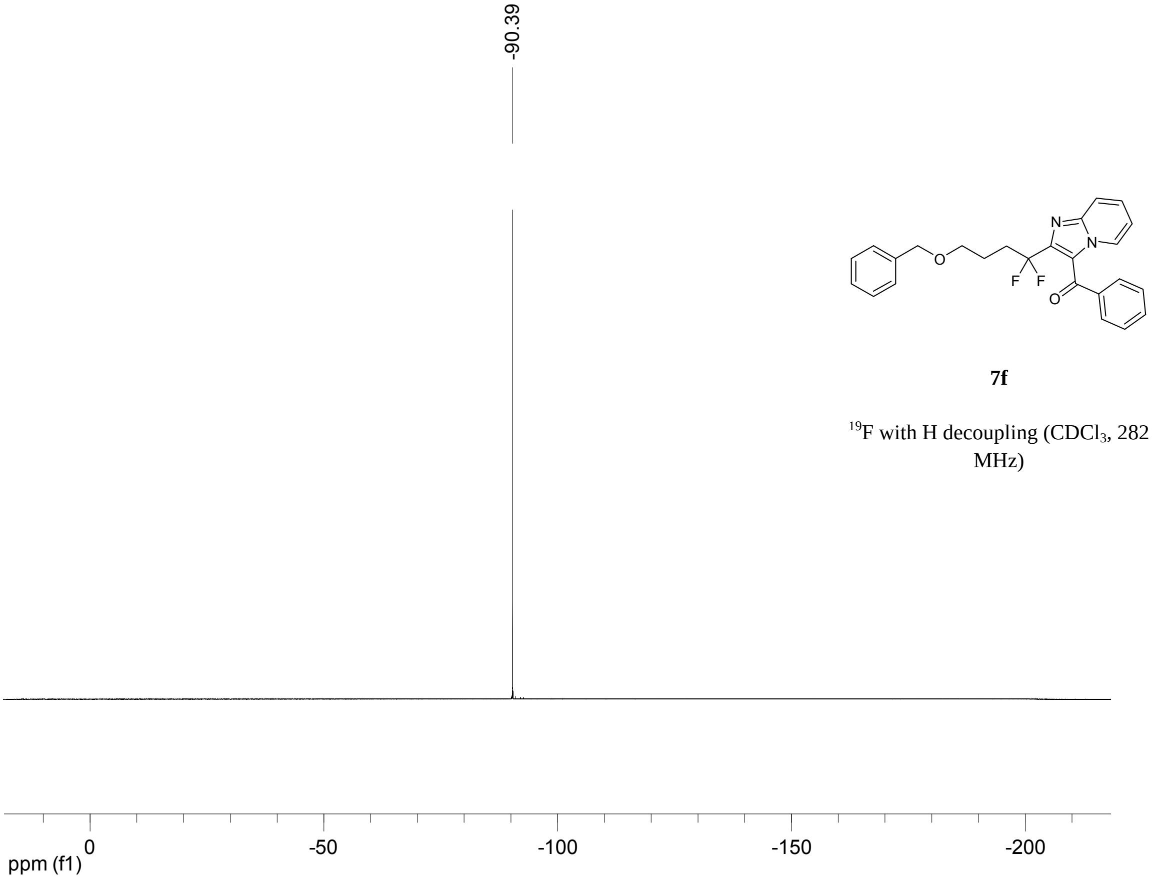

7f

^{19}F with H decoupling ($CDCl_3$, 282 MHz)

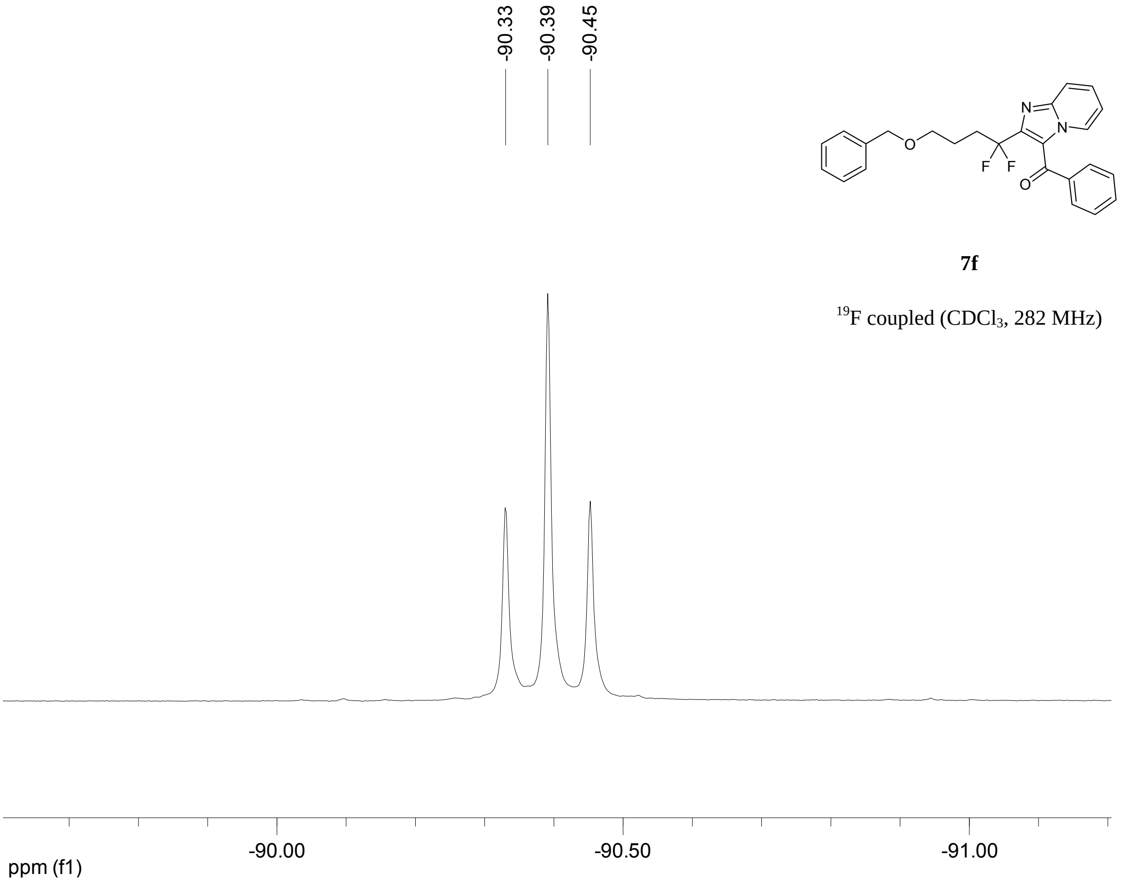

7f

^{19}F coupled ($CDCl_3$, 282 MHz)

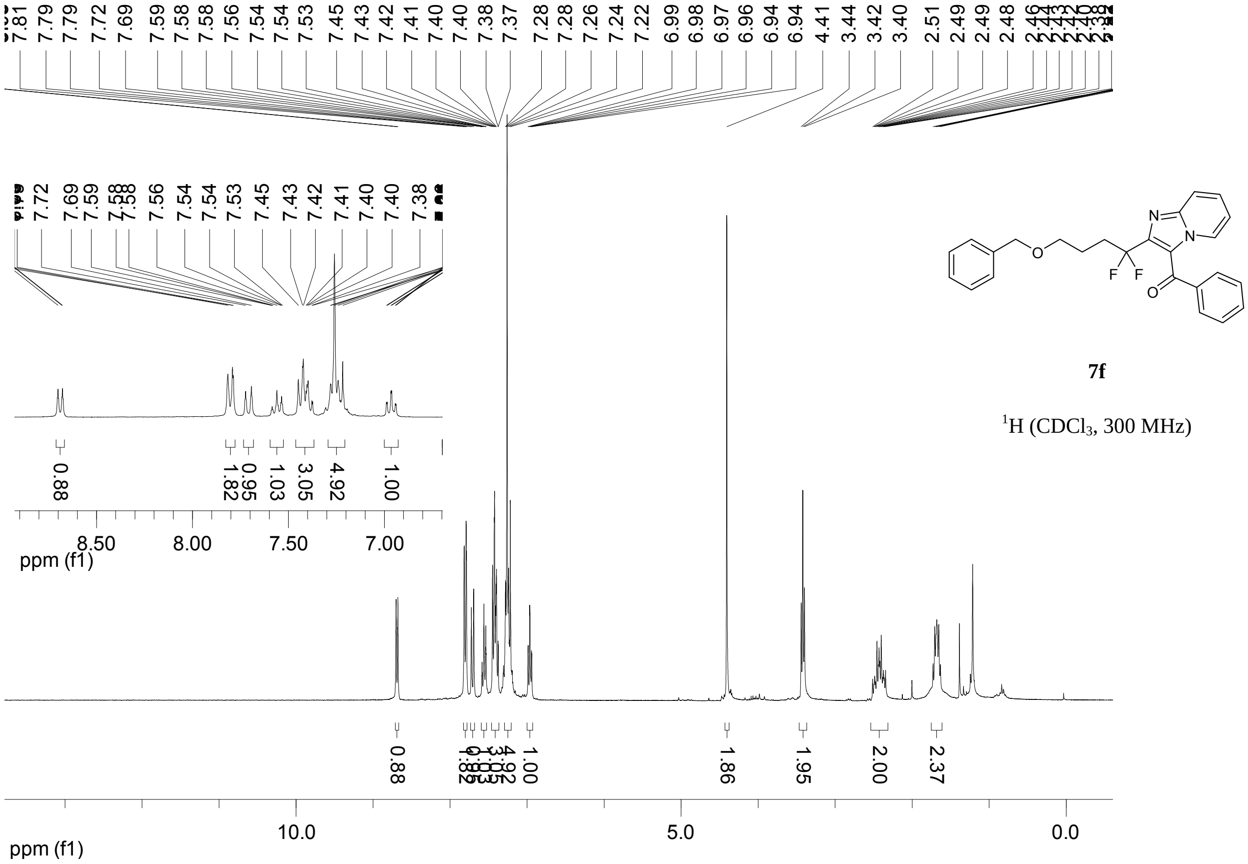

7f

^{1}H (CDCl$_3$, 300 MHz)

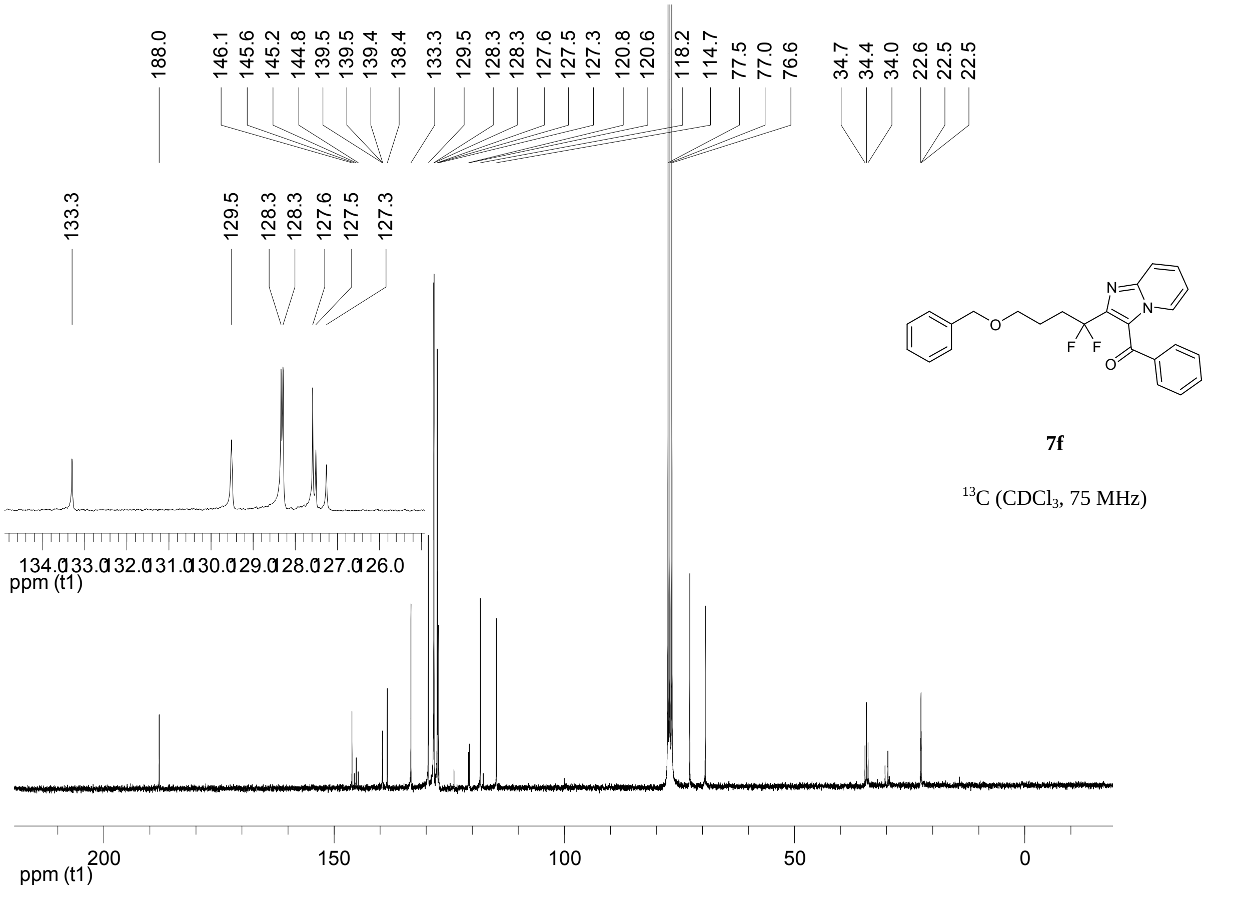

7f

^{13}C (CDCl$_3$, 75 MHz)

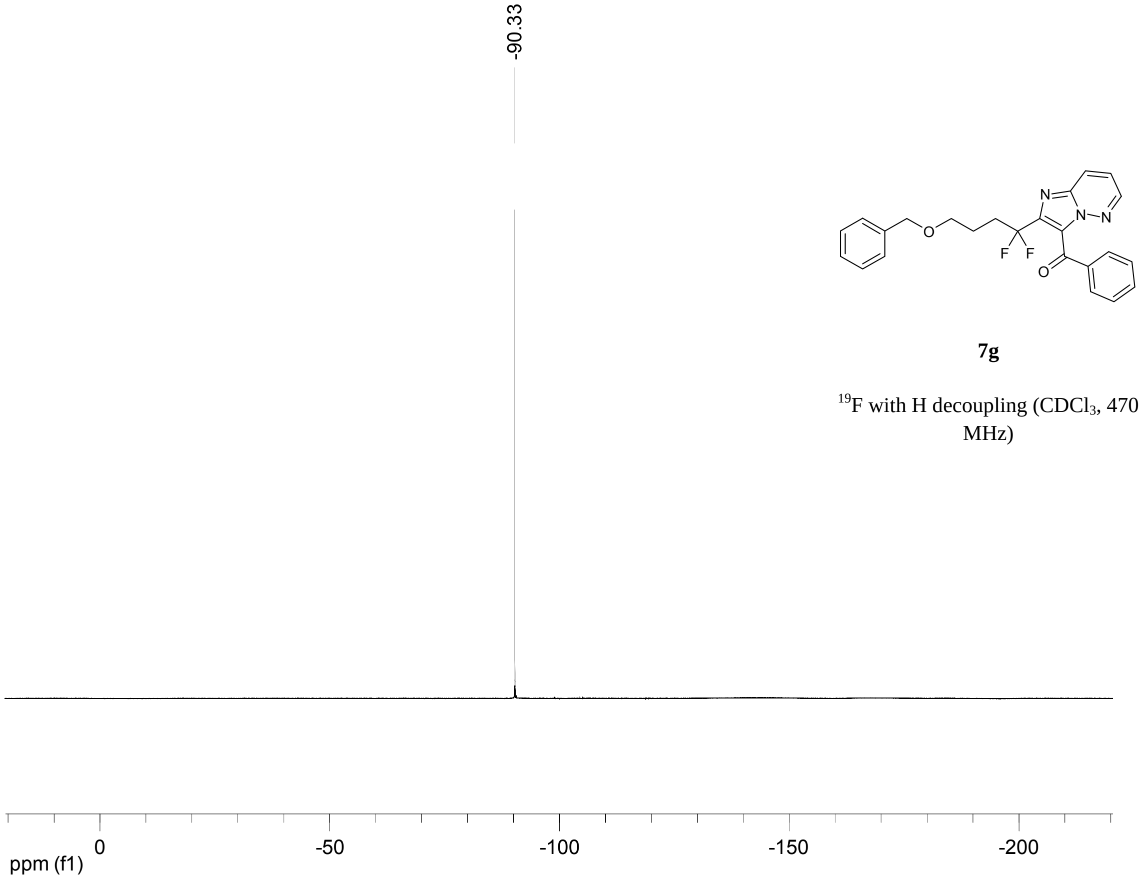

7g

^{19}F with H decoupling ($CDCl_3$, 470 MHz)

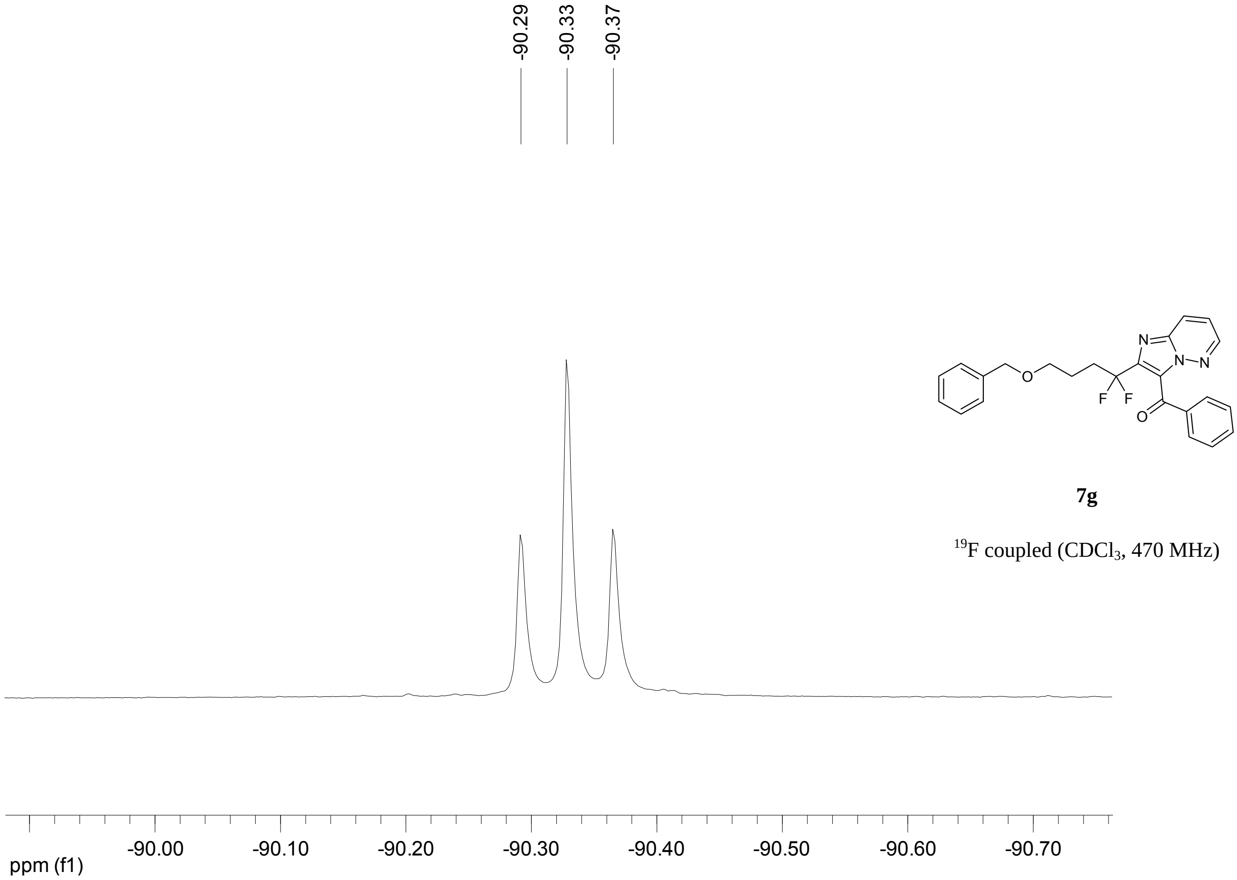

7g

^{19}F coupled ($CDCl_3$, 470 MHz)

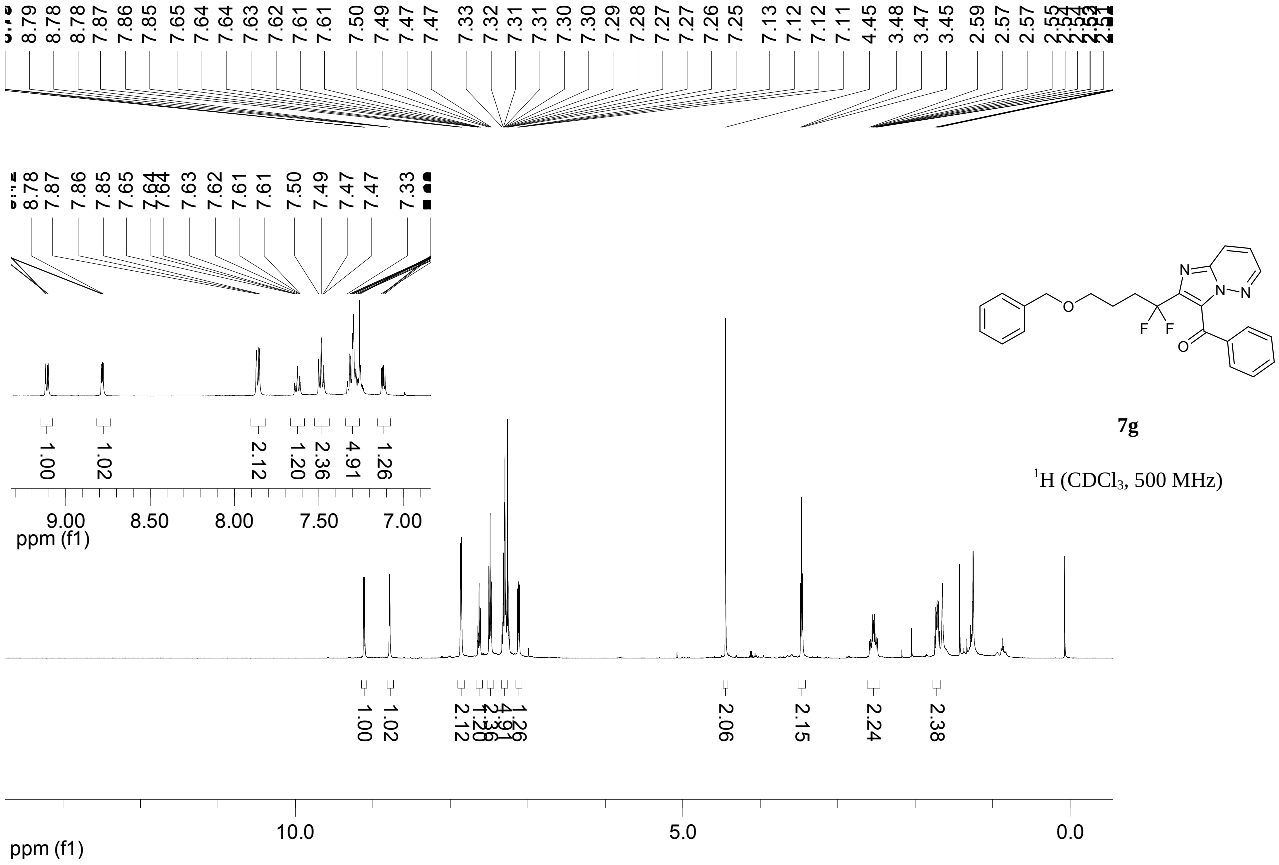

7g

^{1}H ($CDCl_3$, 500 MHz)

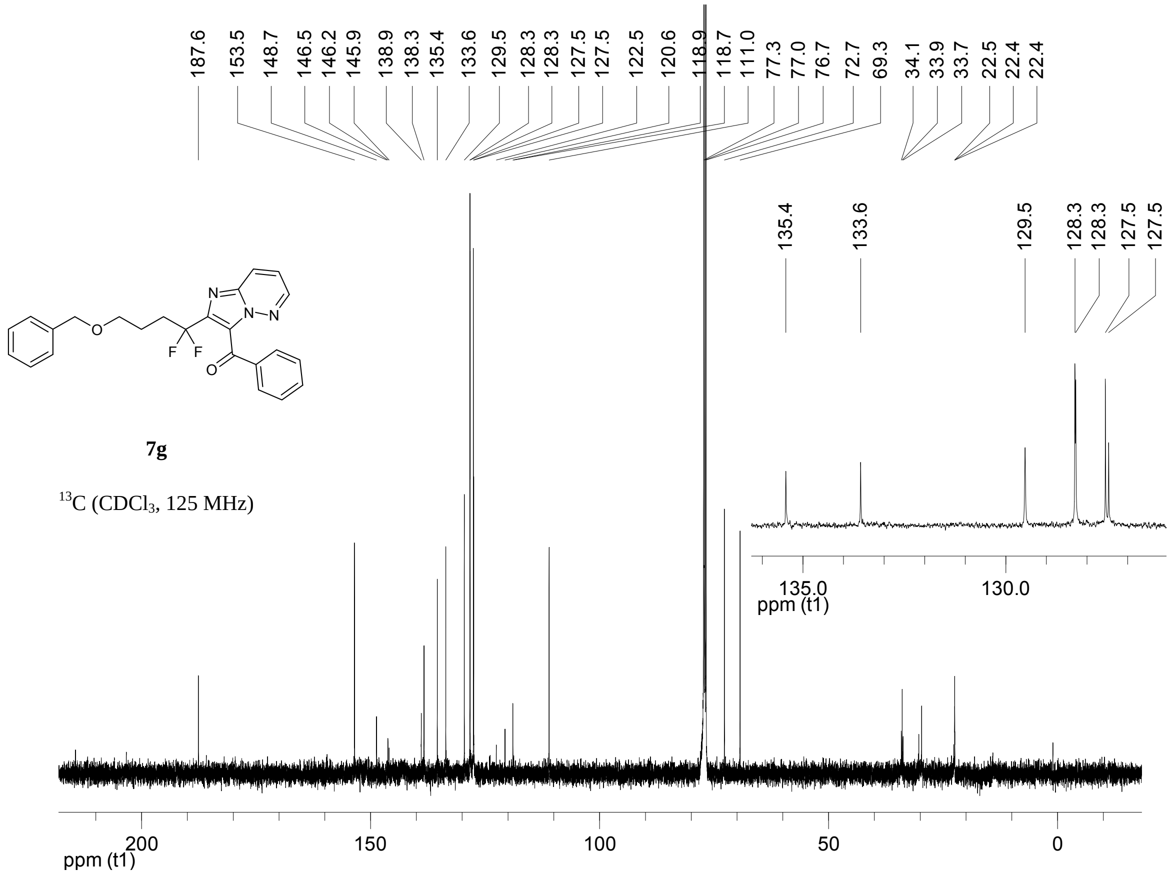

7g

^{13}C ($CDCl_3$, 125 MHz)

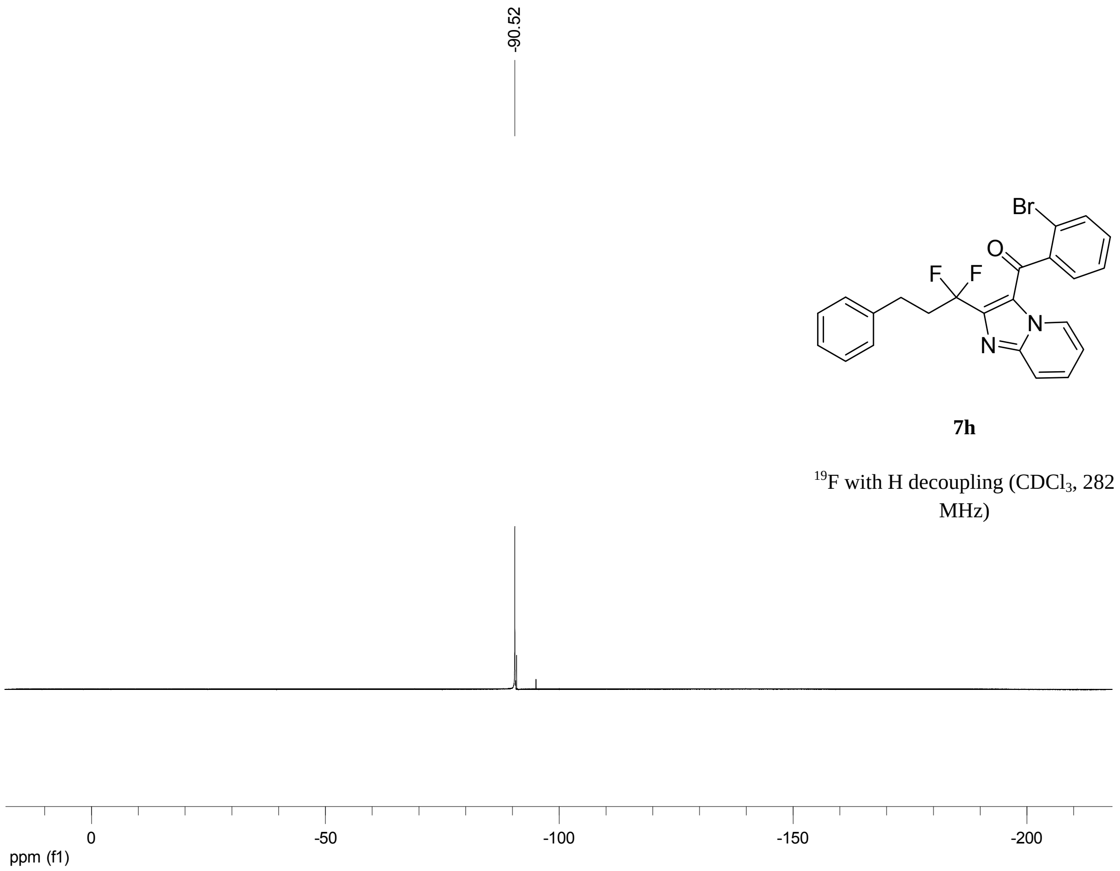
-90.52
Br
O
F F
N
N
7h
19F with H decoupling (CDCl3, 282 MHz)
0
-50
-100
-150
-200
ppm (f1)

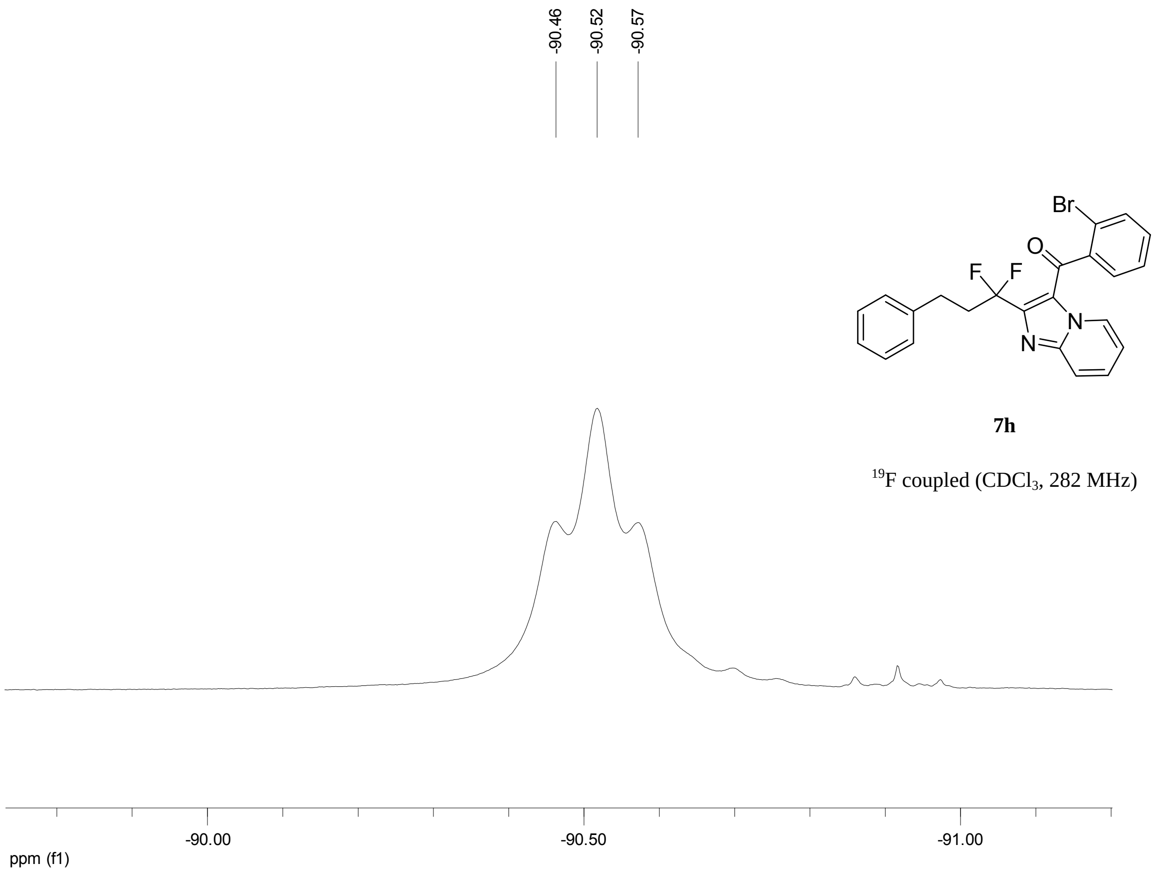
-90.46
-90.52
-90.57
Br
O
F F
N
N
7h
19F coupled (CDCl3, 282 MHz)
-90.00
-90.50
-91.00
ppm (f1)

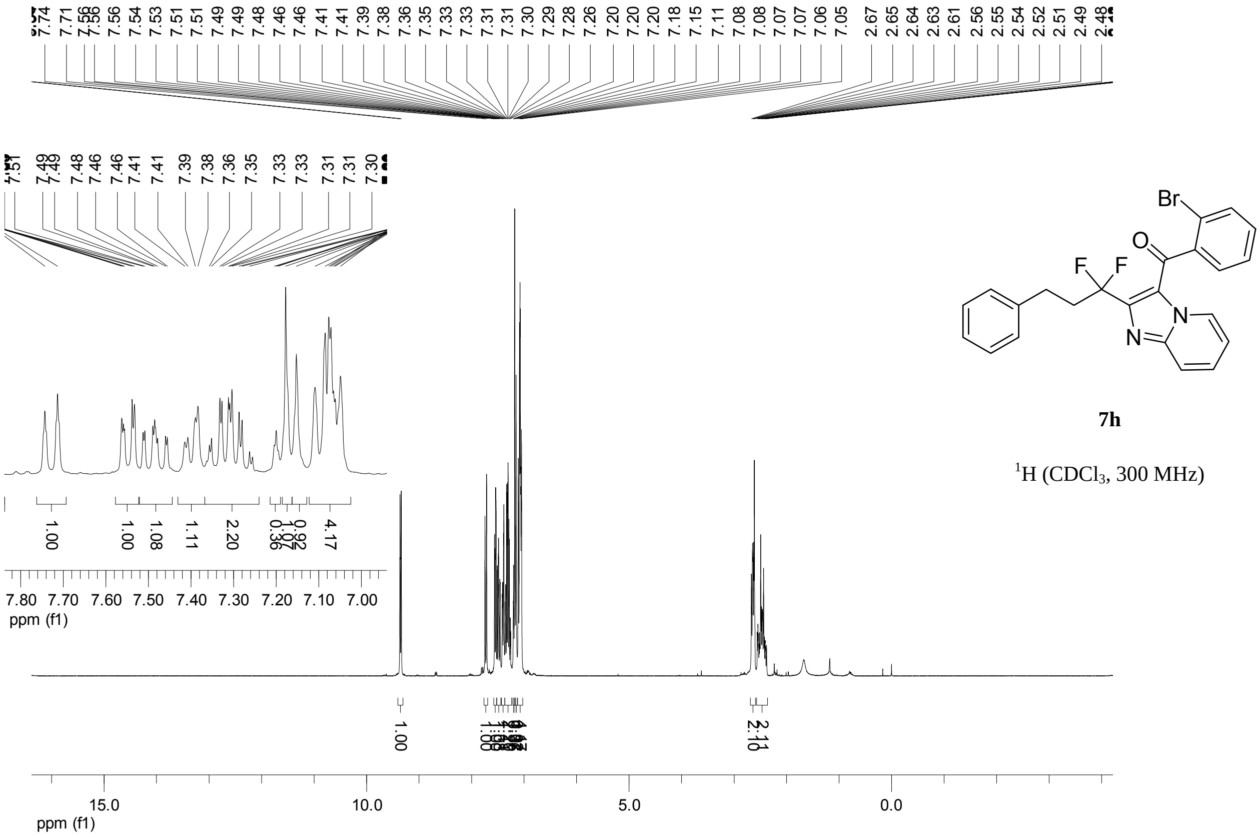

7h

^{1}H ($CDCl_3$, 300 MHz)

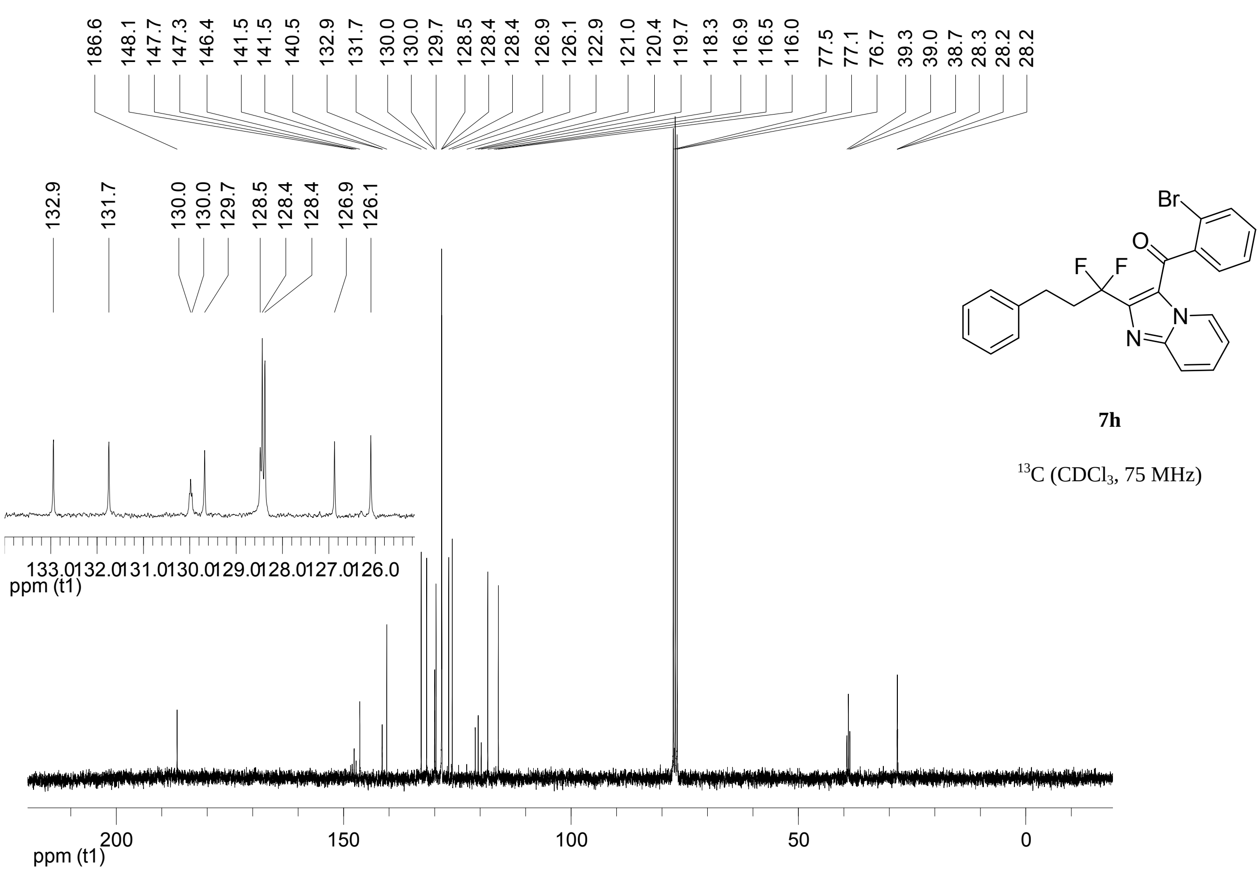

7h

^{13}C ($CDCl_3$, 75 MHz)

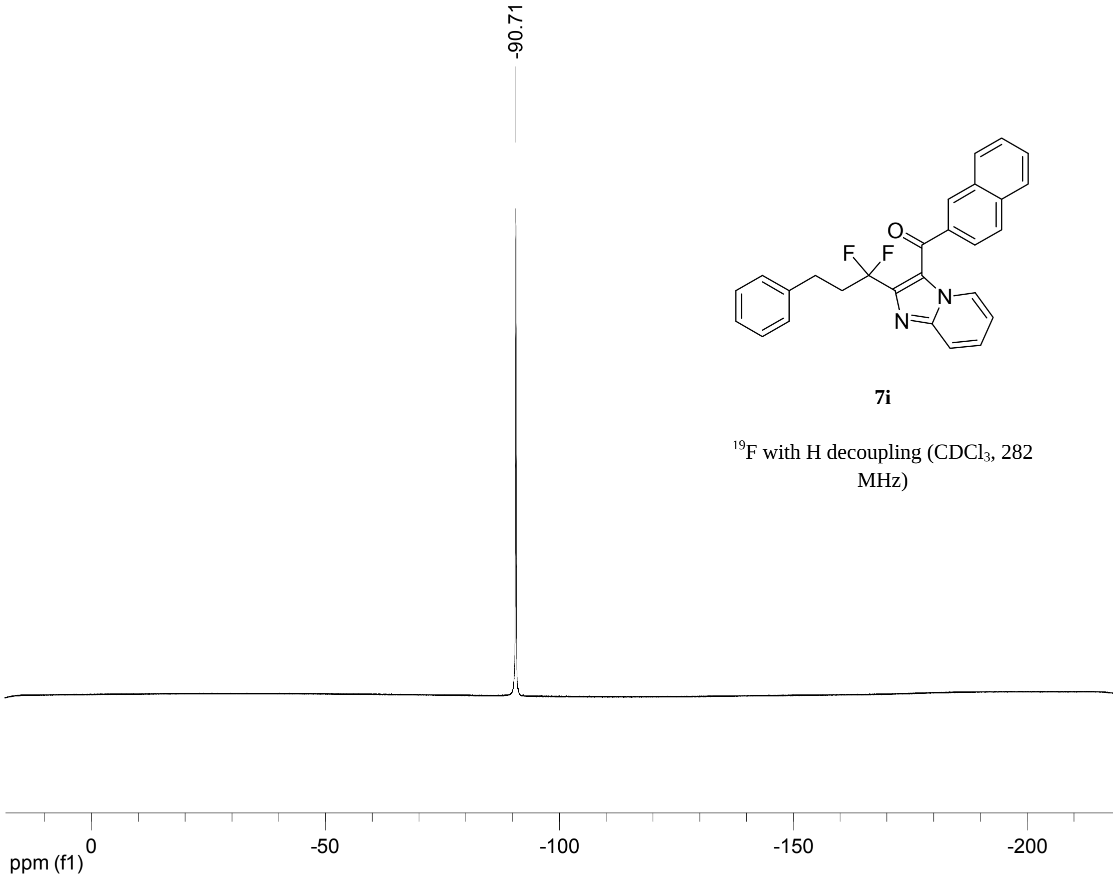

7i

^{19}F with H decoupling ($CDCl_3$, 282 MHz)

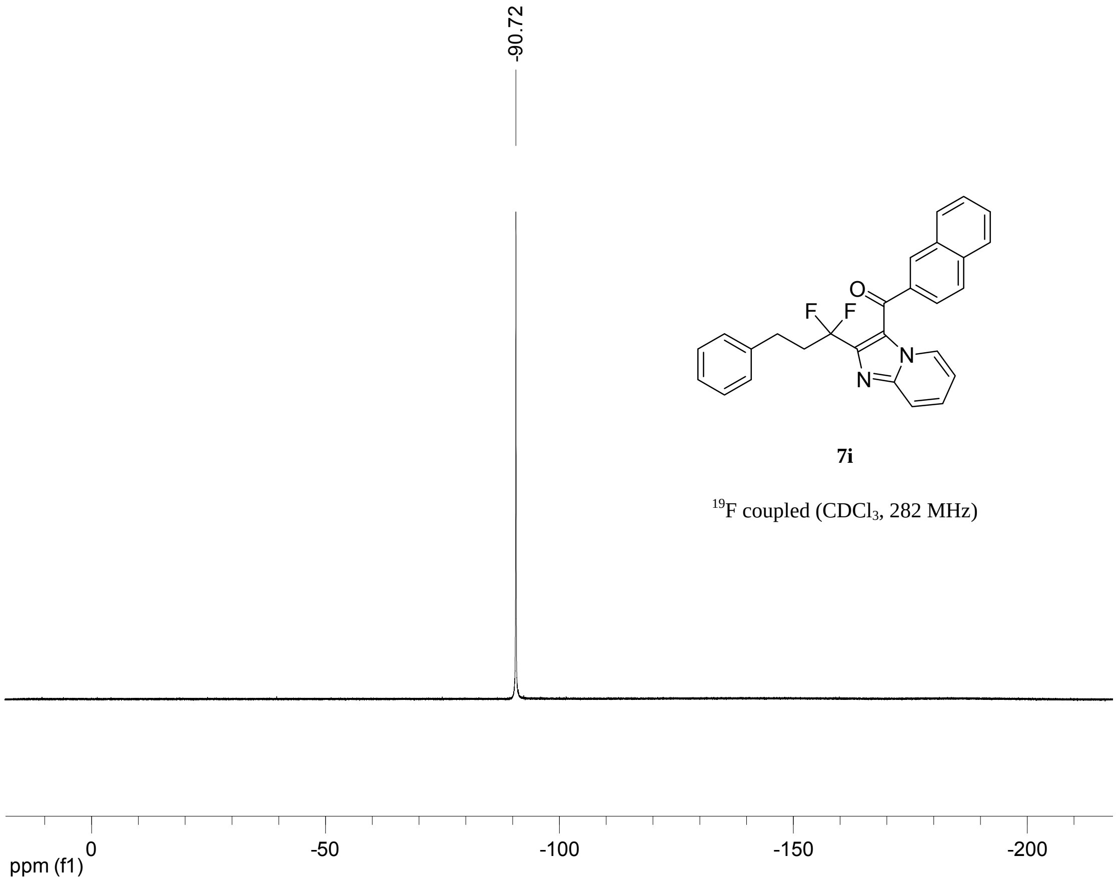

7i

^{19}F coupled ($CDCl_3$, 282 MHz)

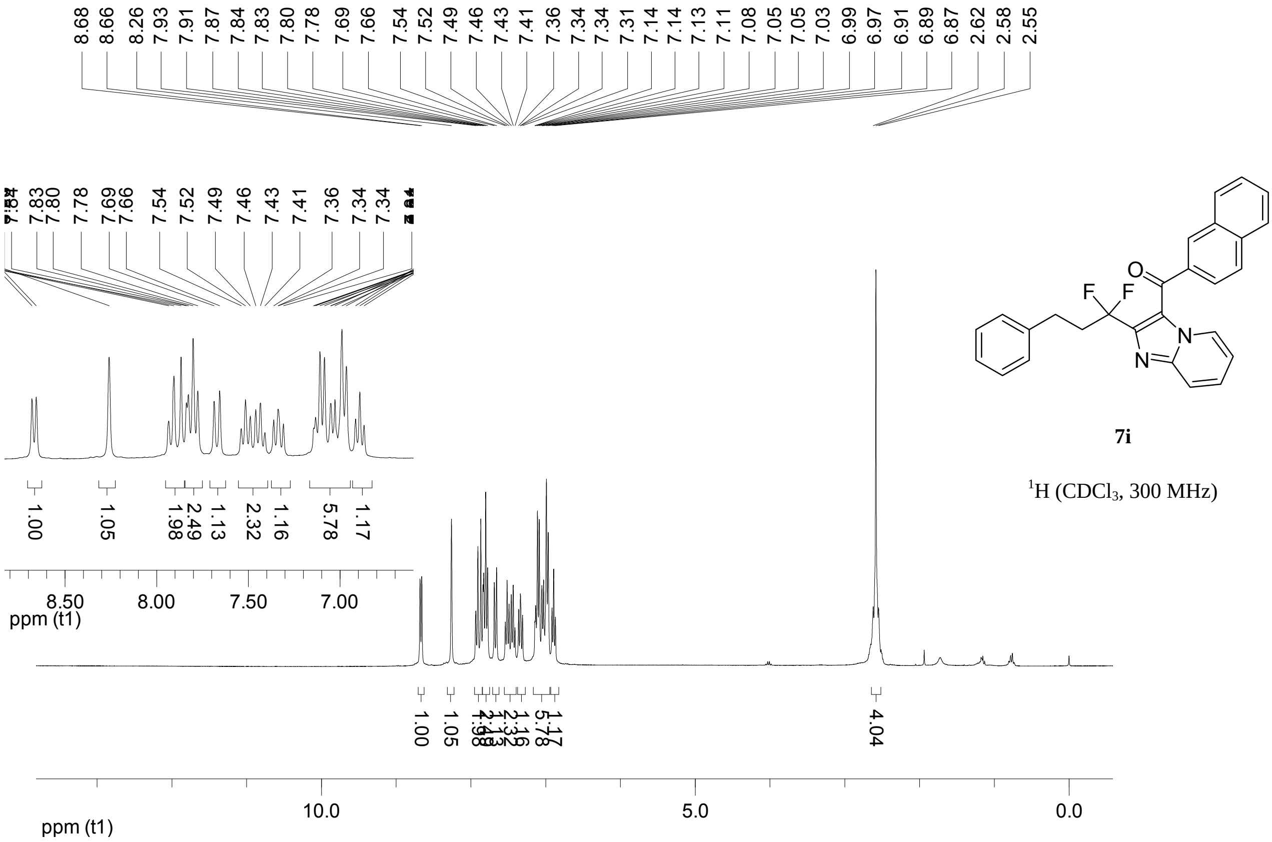

7i

^{1}H (CDCl$_3$, 300 MHz)

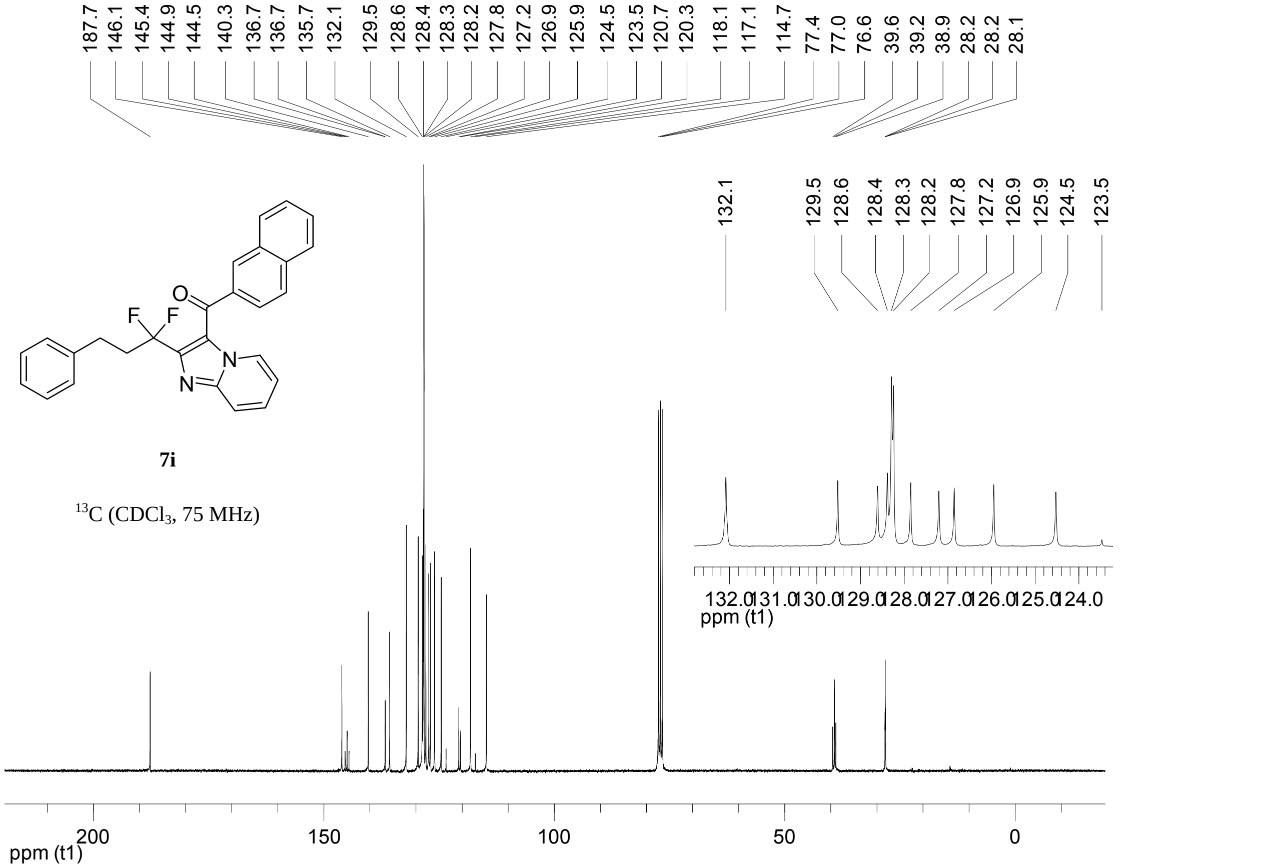

7i

^{13}C (CDCl$_3$, 75 MHz)

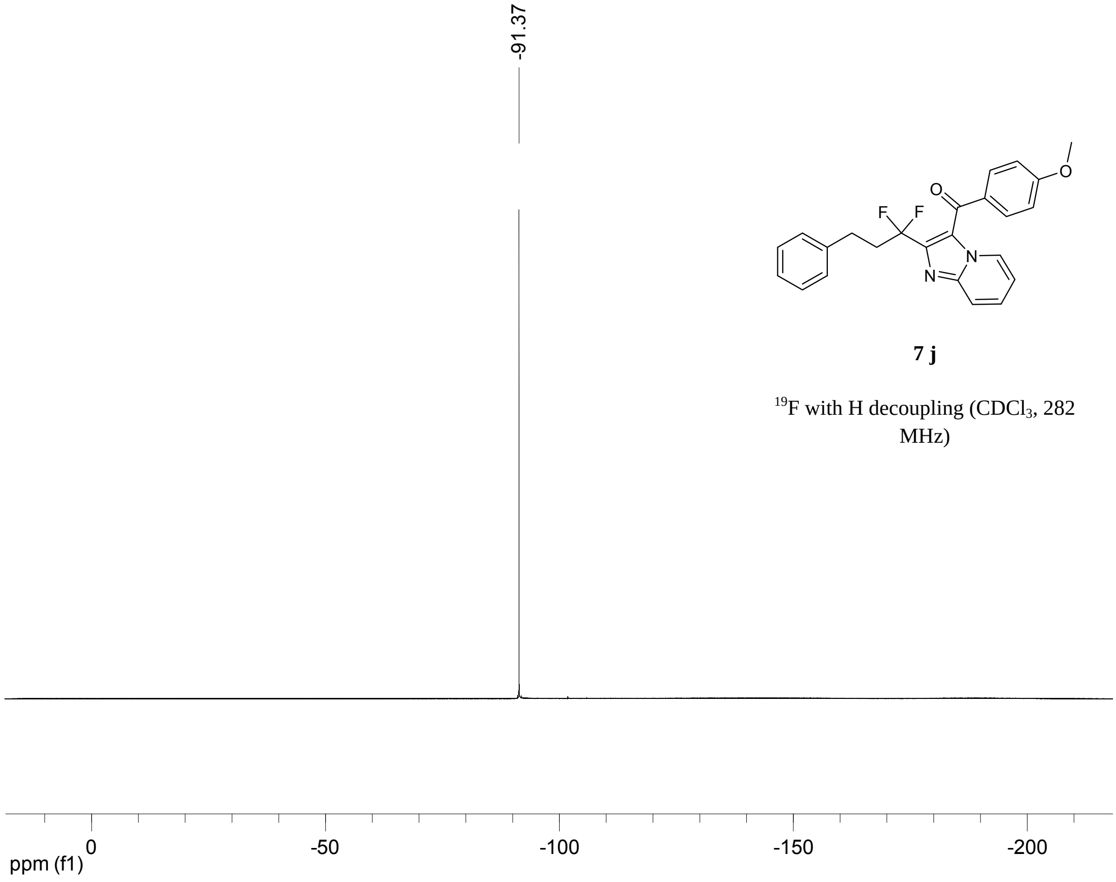

7 j

^{19}F with H decoupling ($CDCl_3$, 282 MHz)

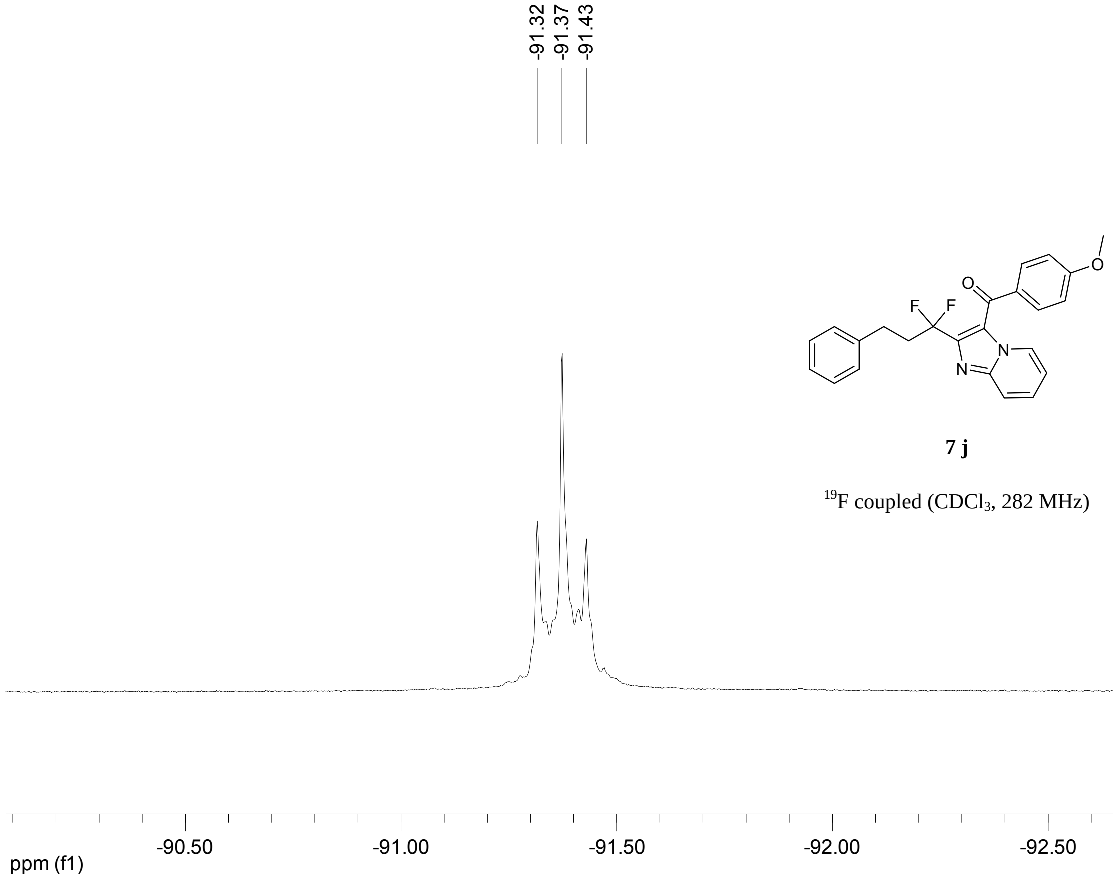

7 j

^{19}F coupled ($CDCl_3$, 282 MHz)

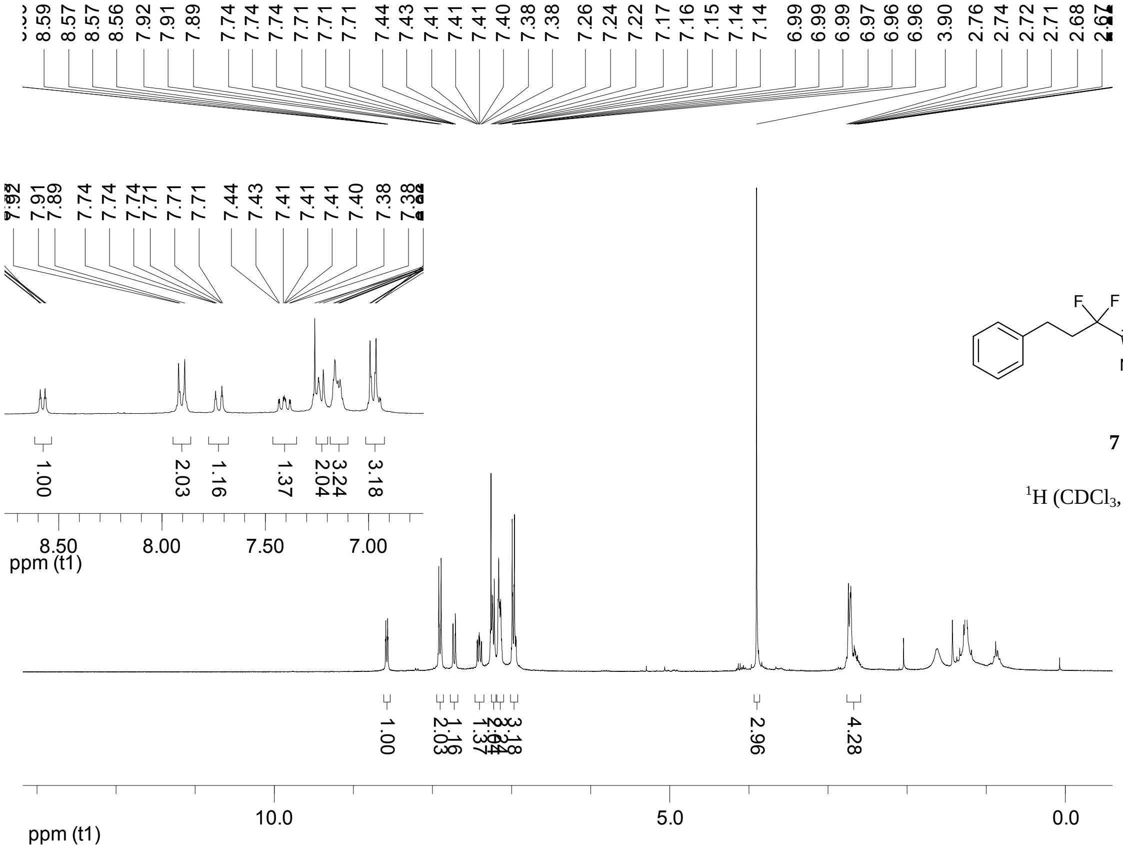

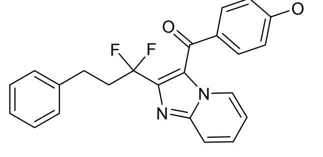

7 j

^{1}H (CDCl$_3$, 300 MHz)

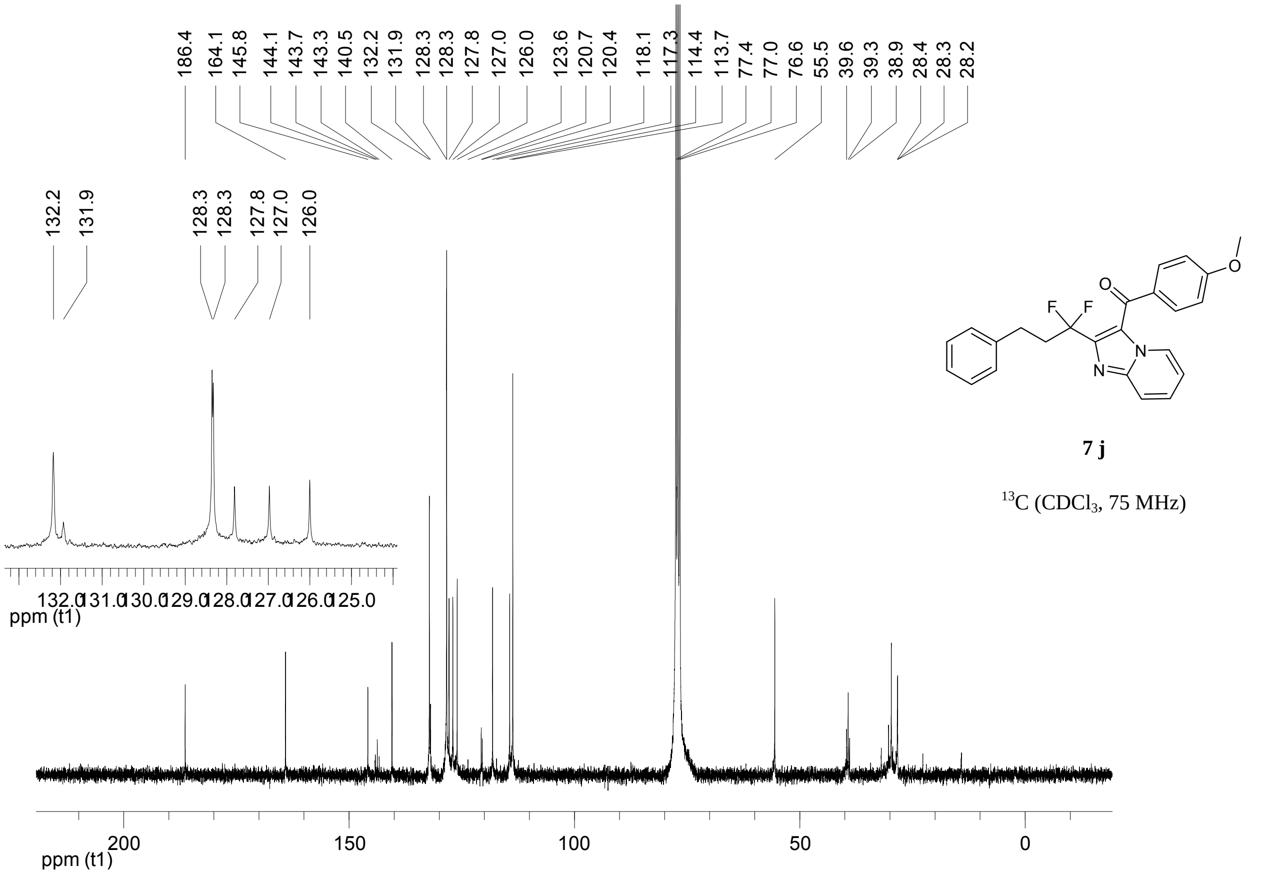

7 j

^{13}C (CDCl$_3$, 75 MHz)

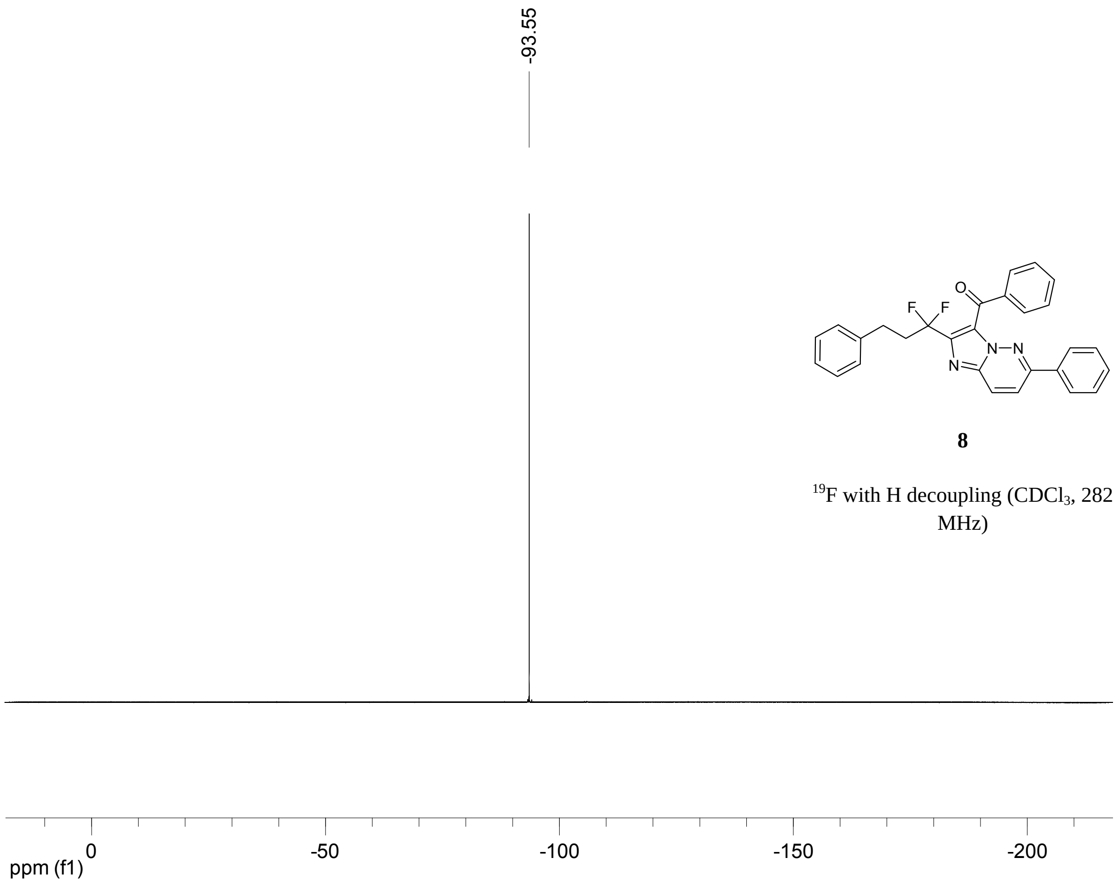

8

^{19}F with H decoupling ($CDCl_3$, 282 MHz)

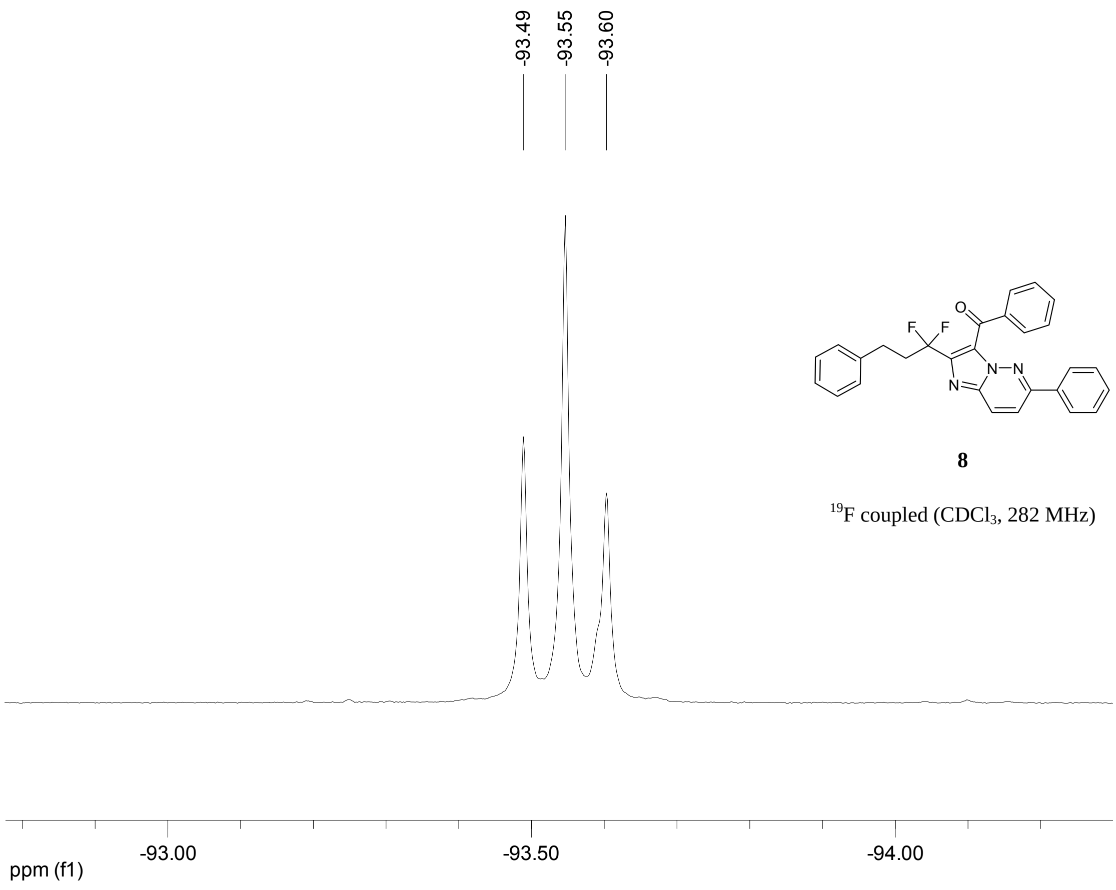

8

^{19}F coupled ($CDCl_3$, 282 MHz)

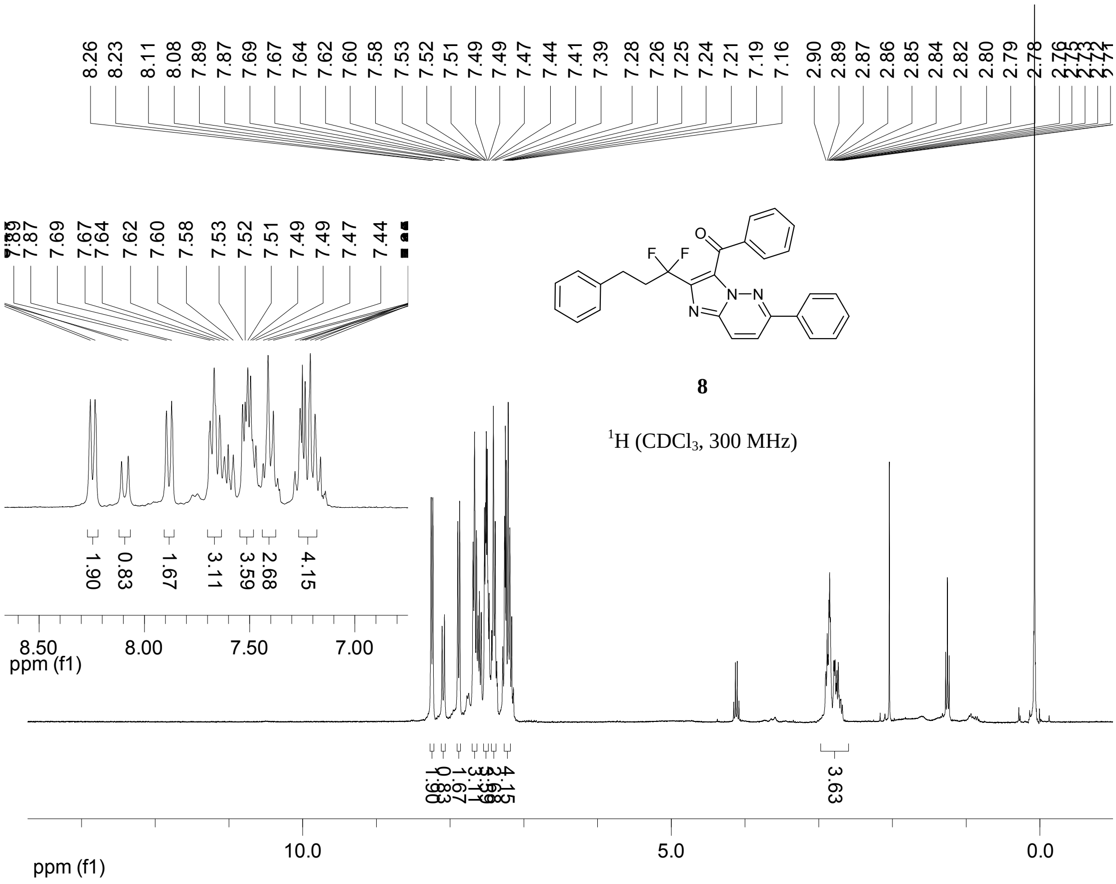
8.26 8.23 8.11 8.08 7.89 7.87 7.69 7.67 7.64 7.62 7.60 7.58 7.53 7.52 7.51 7.49 7.49 7.47 7.44 7.41 7.39 7.28 7.26 7.25 7.24 7.21 7.19 7.16 2.90 2.89 2.87 2.86 2.85 2.84 2.82 2.80 2.79 2.78
7.87 7.69 7.67 7.64 7.62 7.60 7.58 7.53 7.52 7.51 7.49 7.49 7.47 7.44
O
F F
N
N–N
8
^{1}H (CDCl$_3$, 300 MHz)
1.90 0.83 1.67 3.11 3.59 2.68 4.15
8.50 8.00 7.50 7.00
ppm (f1)
3.63
10.0 5.0 0.0
ppm (f1)

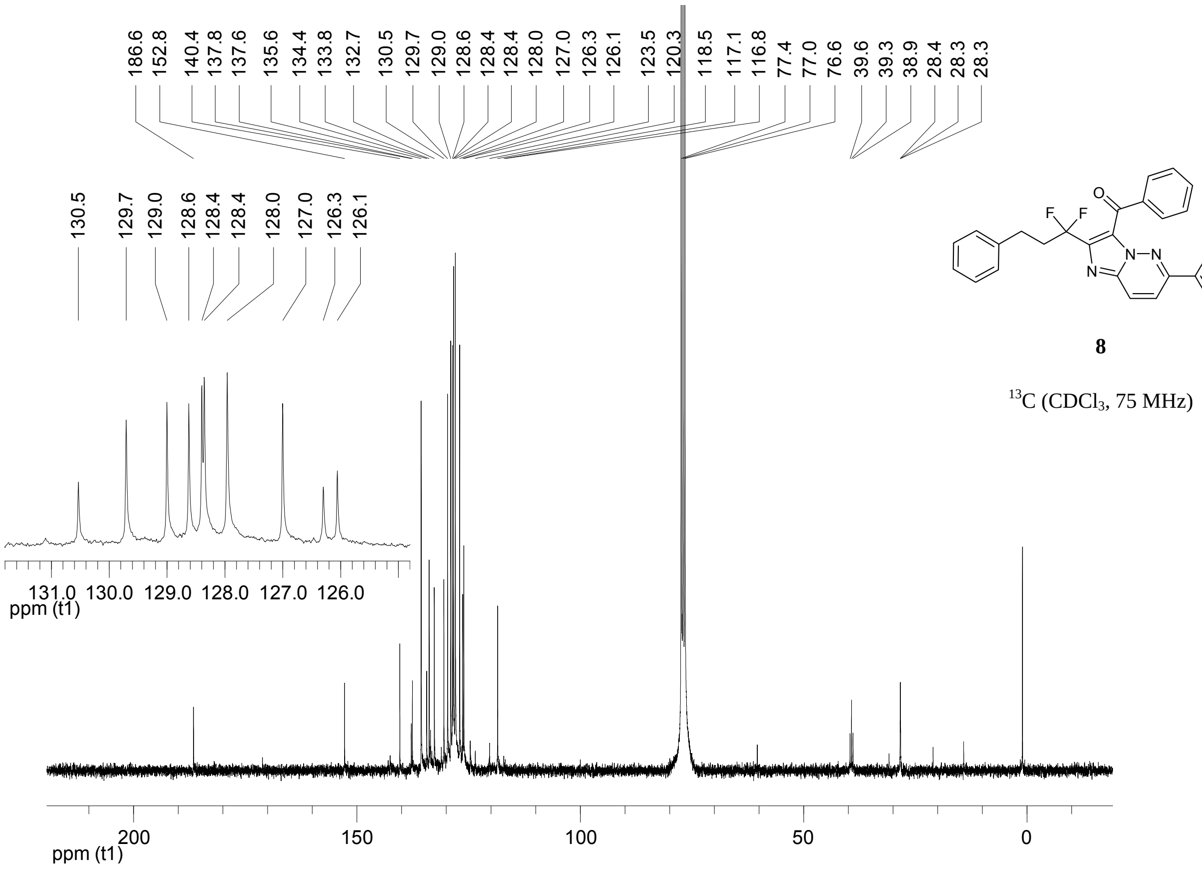
186.6 152.8 140.4 137.8 137.6 135.6 134.4 133.8 132.7 130.5 129.7 129.0 128.6 128.4 128.4 128.0 127.0 126.3 126.1 123.5 120.3 118.5 117.1 116.8 77.4 77.0 76.6 39.6 39.3 38.9 28.4 28.3 28.3
130.5 129.7 129.0 128.6 128.4 128.4 128.0 127.0 126.3 126.1
131.0 130.0 129.0 128.0 127.0 126.0
ppm (t1)
O
F F
N
N–N
8
^{13}C (CDCl$_3$, 75 MHz)
200 150 100 50 0
ppm (t1)

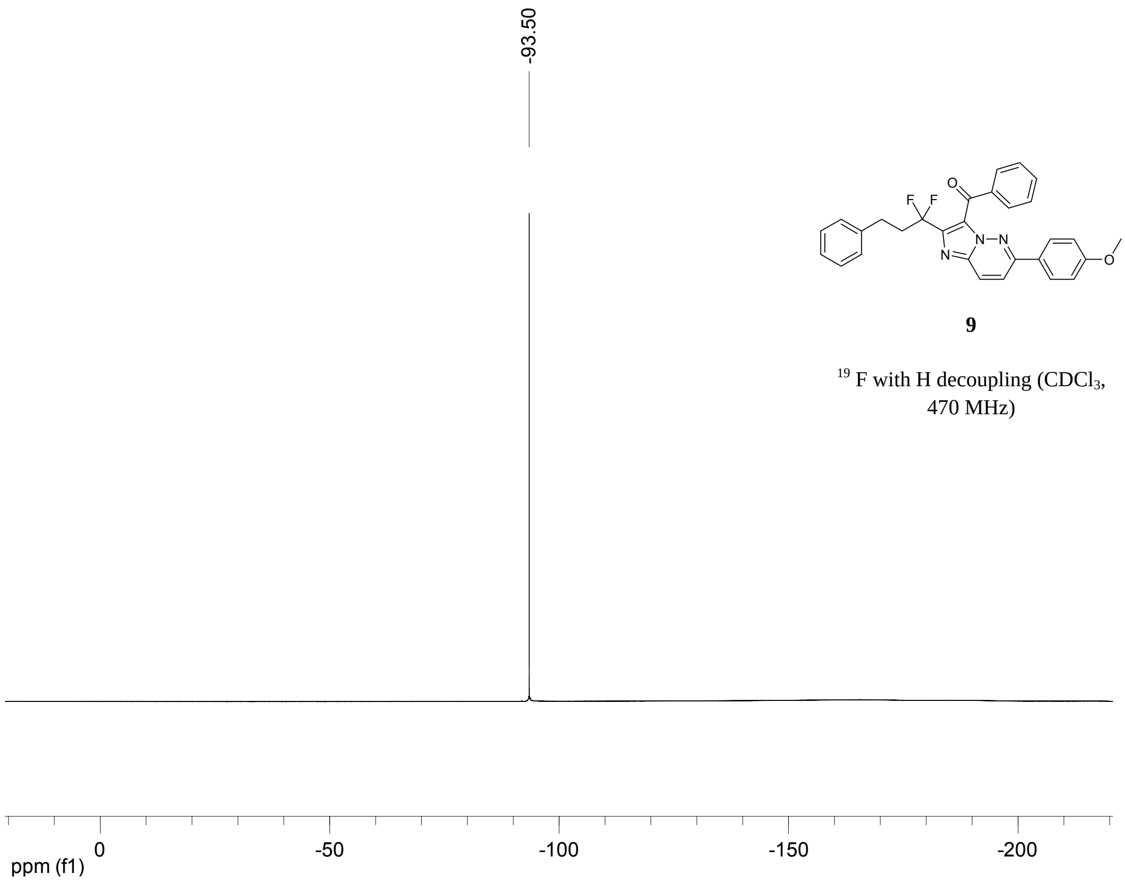

9

[19] F with H decoupling ($CDCl_3$, 470 MHz)

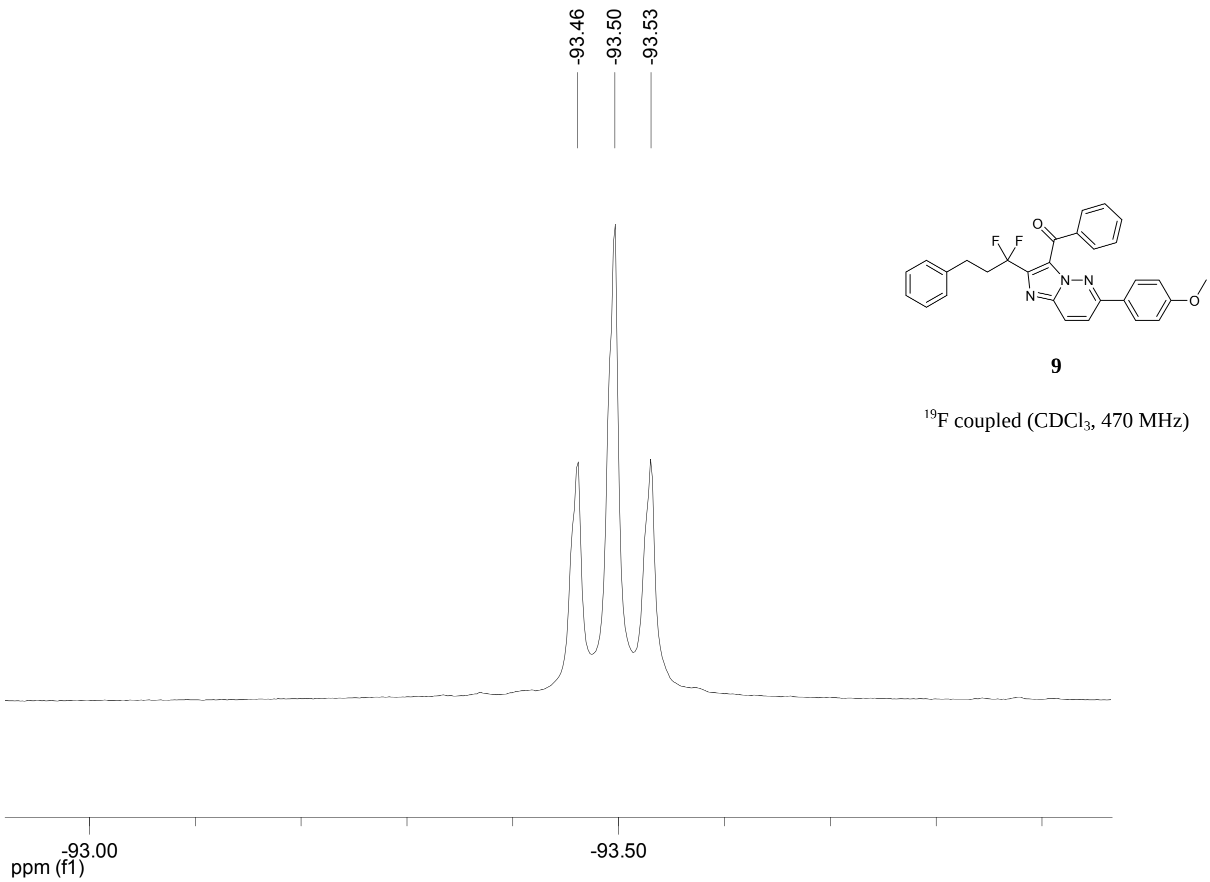

9

^{19}F coupled ($CDCl_3$, 470 MHz)

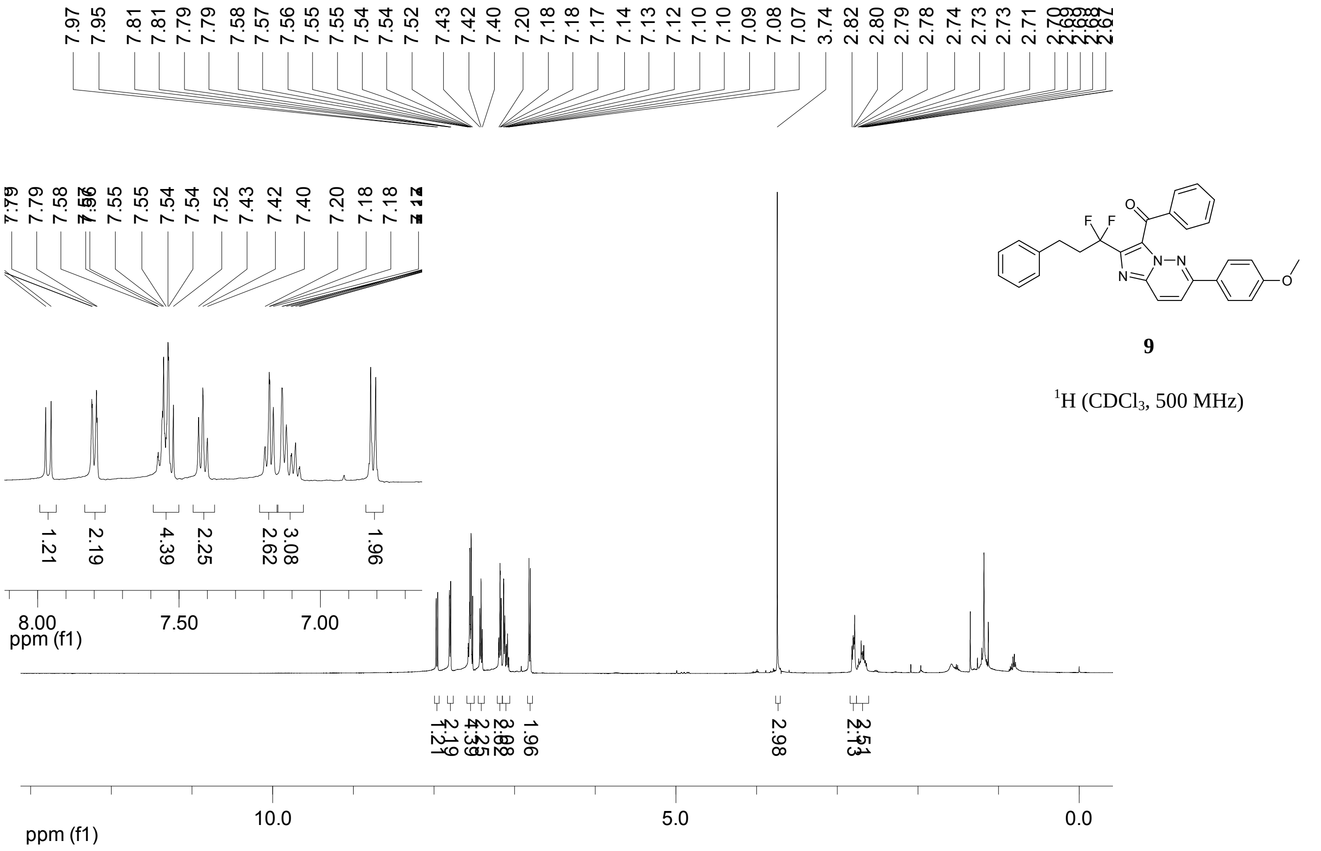

9

^{1}H ($CDCl_3$, 500 MHz)

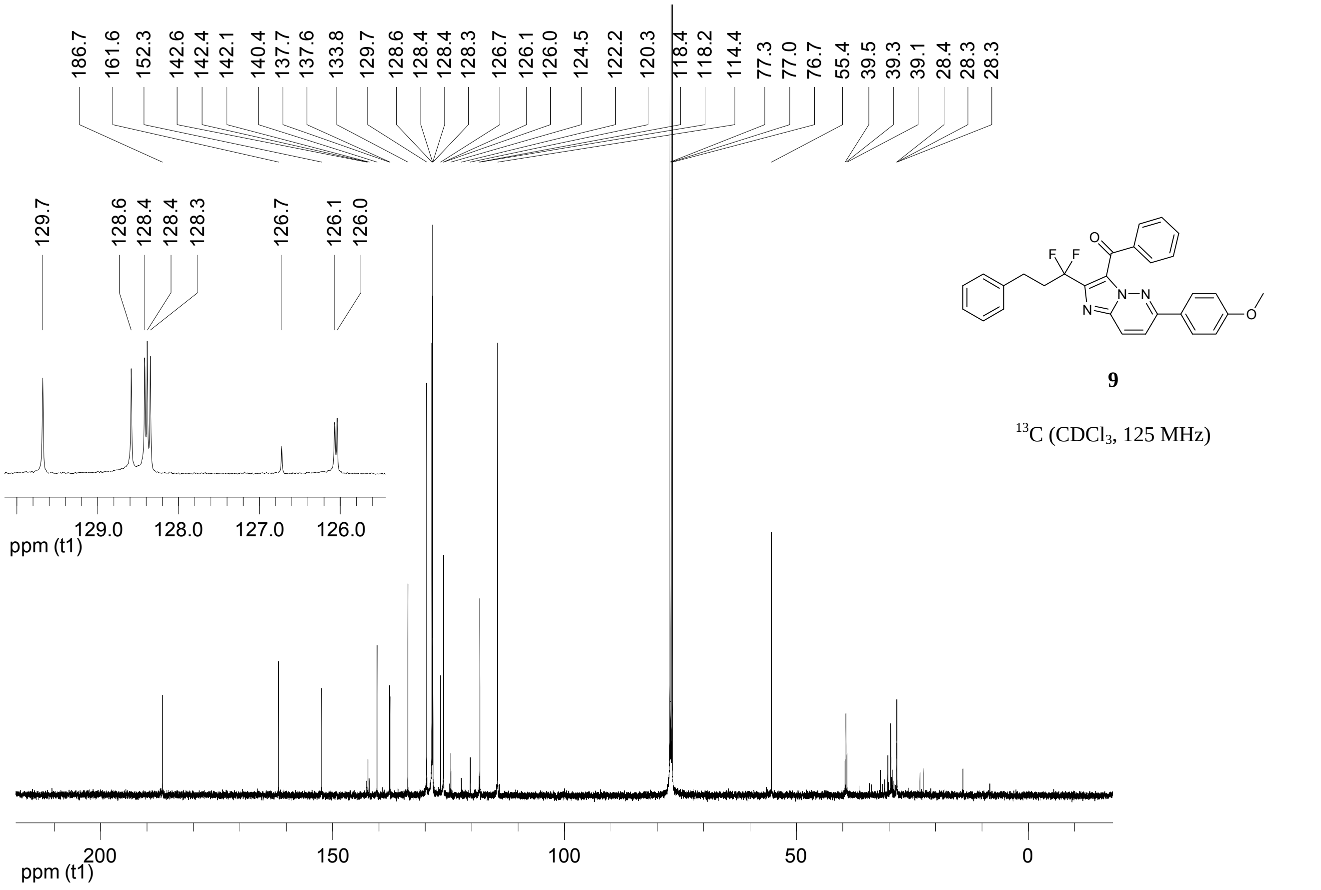

9

^{13}C ($CDCl_3$, 125 MHz)

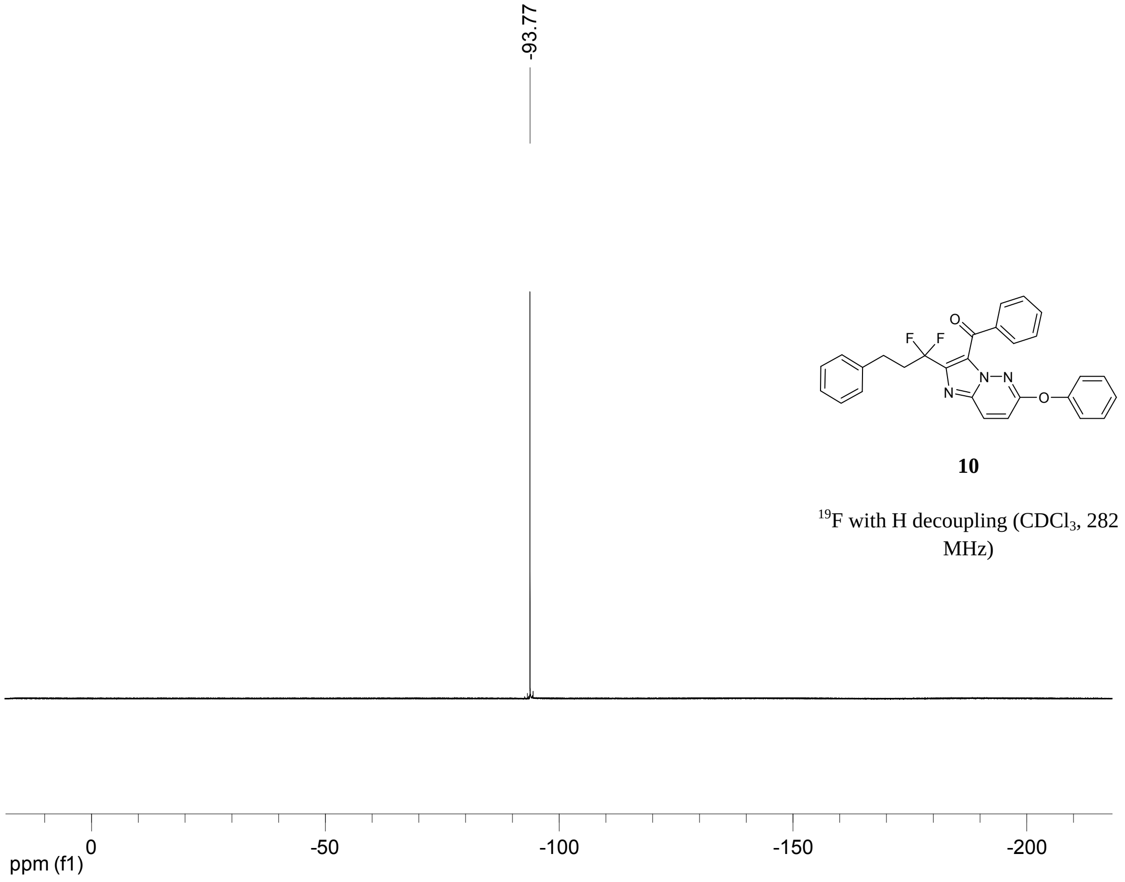

10

^{19}F with H decoupling ($CDCl_3$, 282 MHz)

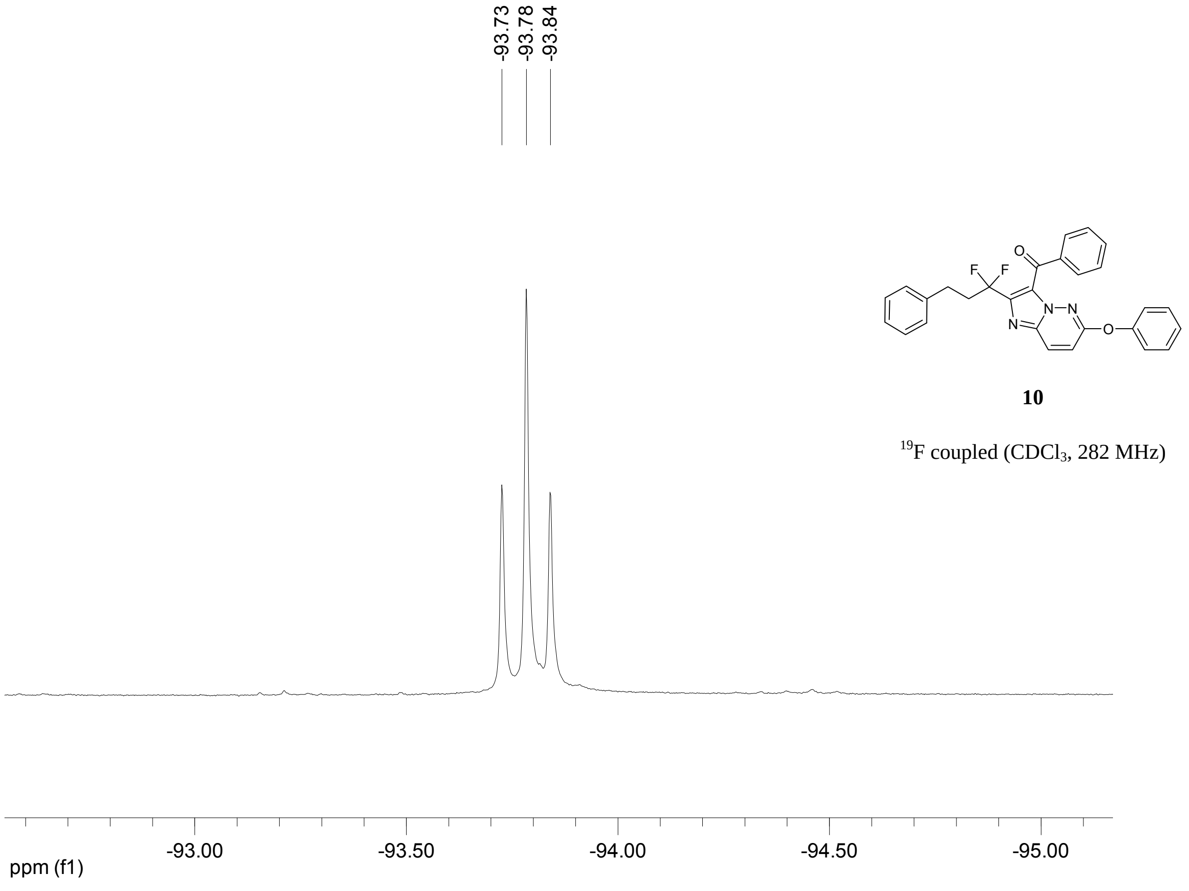

10

^{19}F coupled ($CDCl_3$, 282 MHz)

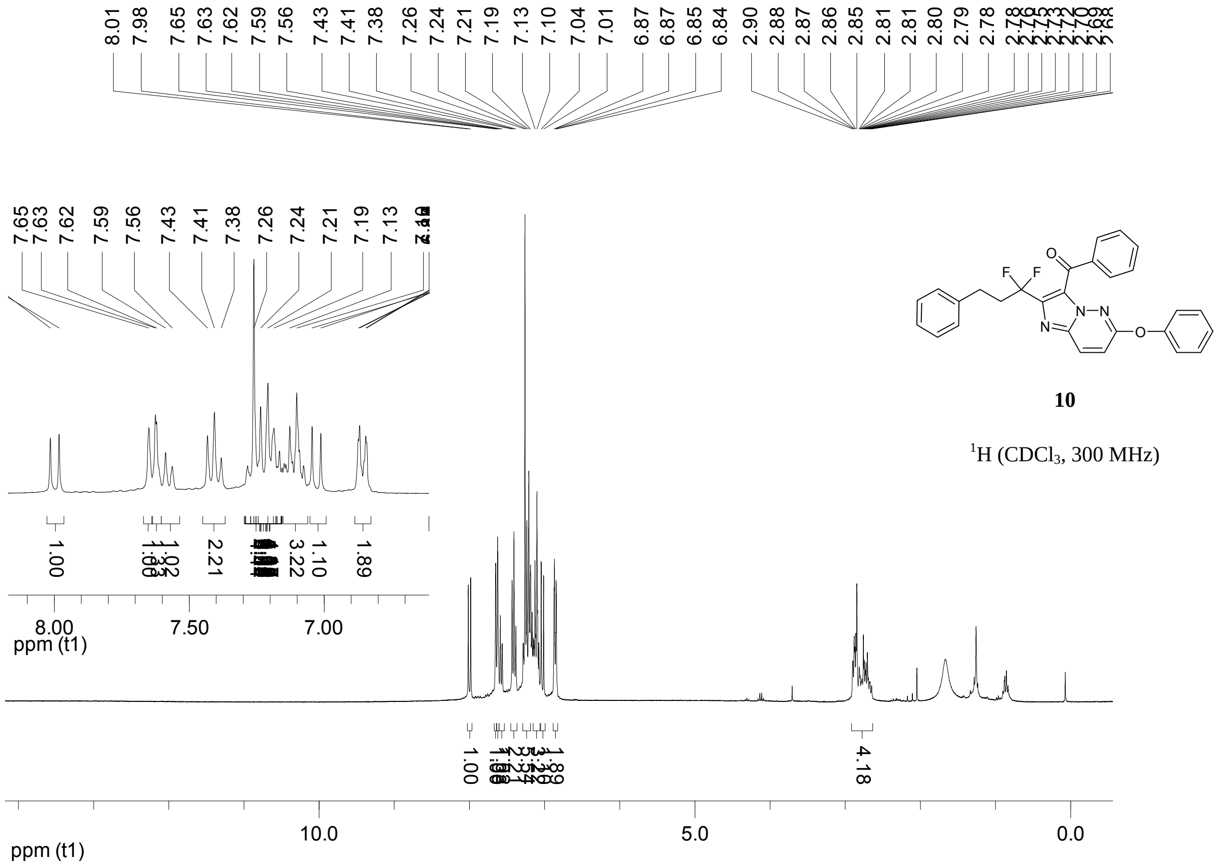

10

^{1}H ($CDCl_3$, 300 MHz)

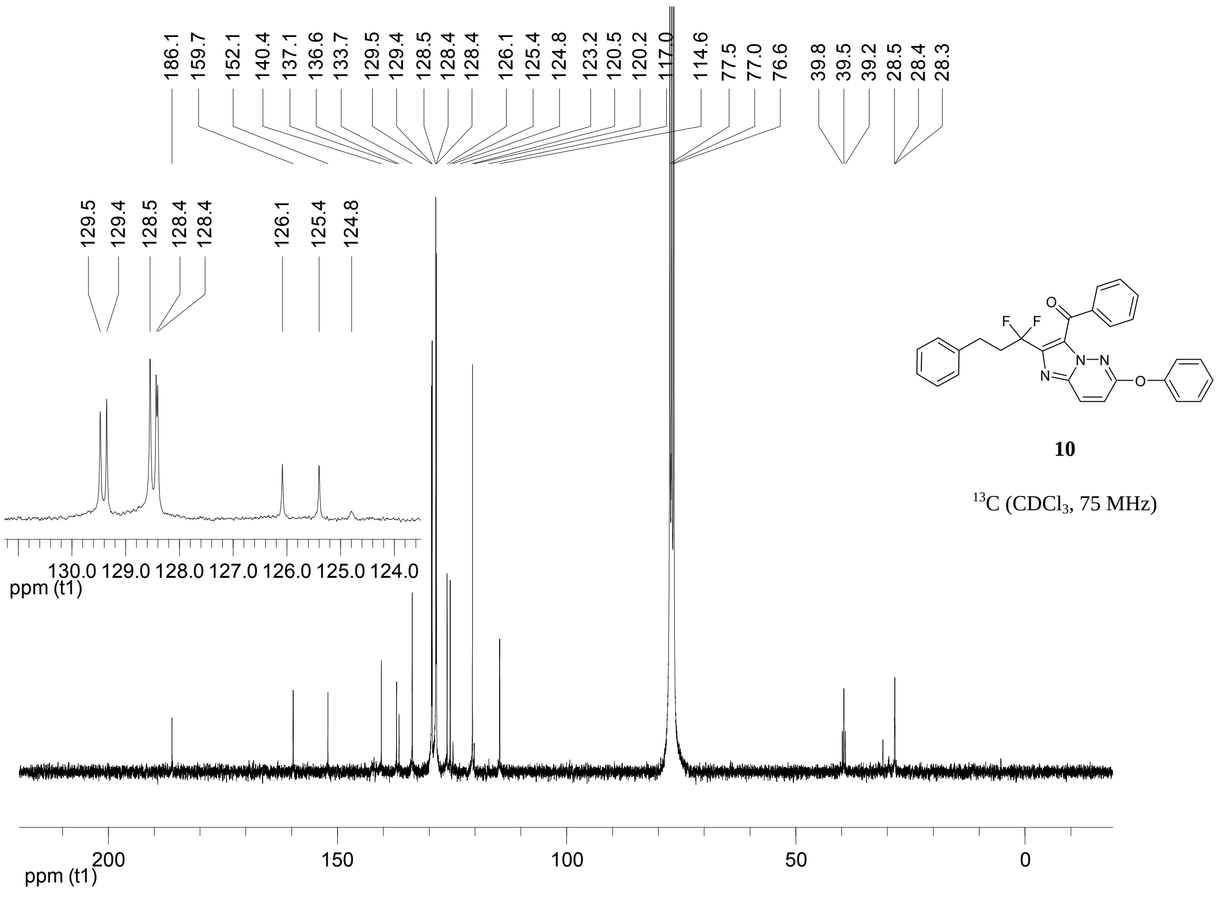

10

^{13}C ($CDCl_3$, 75 MHz)

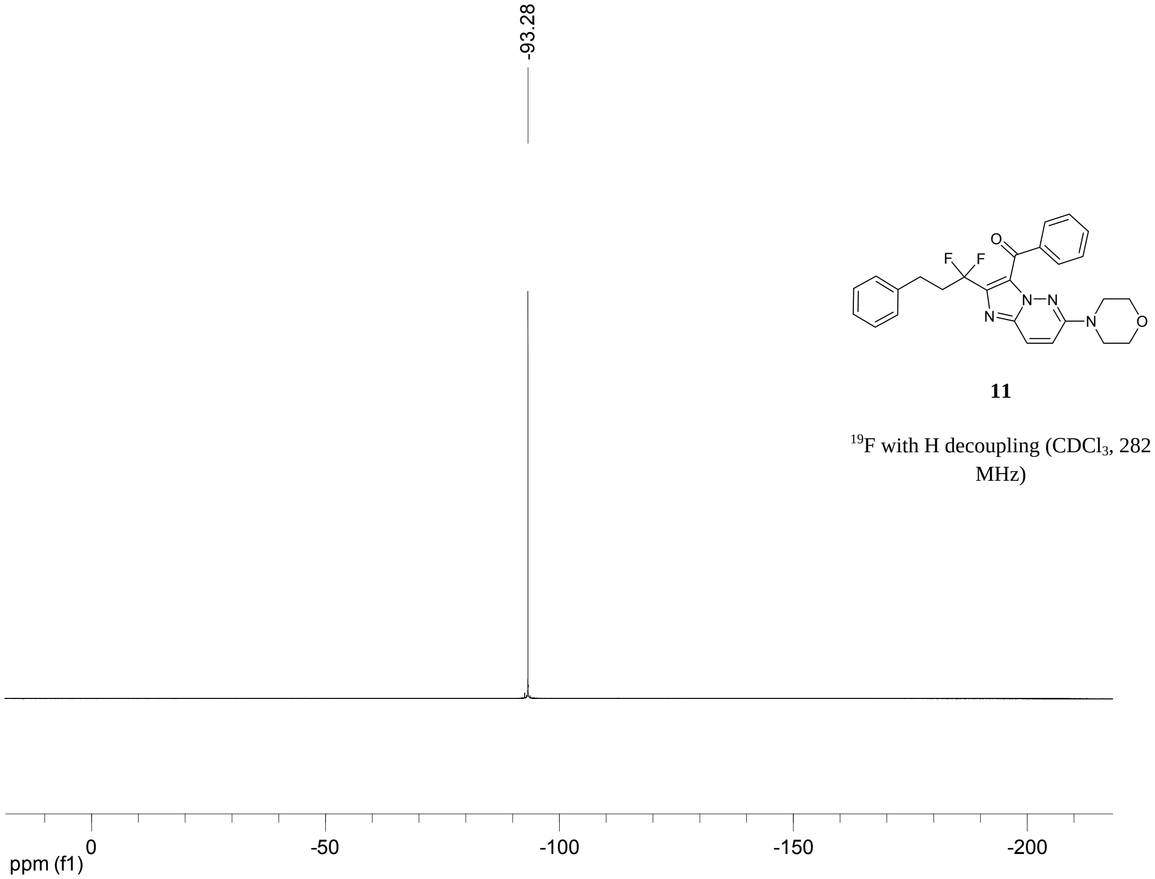

11

^{19}F with H decoupling ($CDCl_3$, 282 MHz)

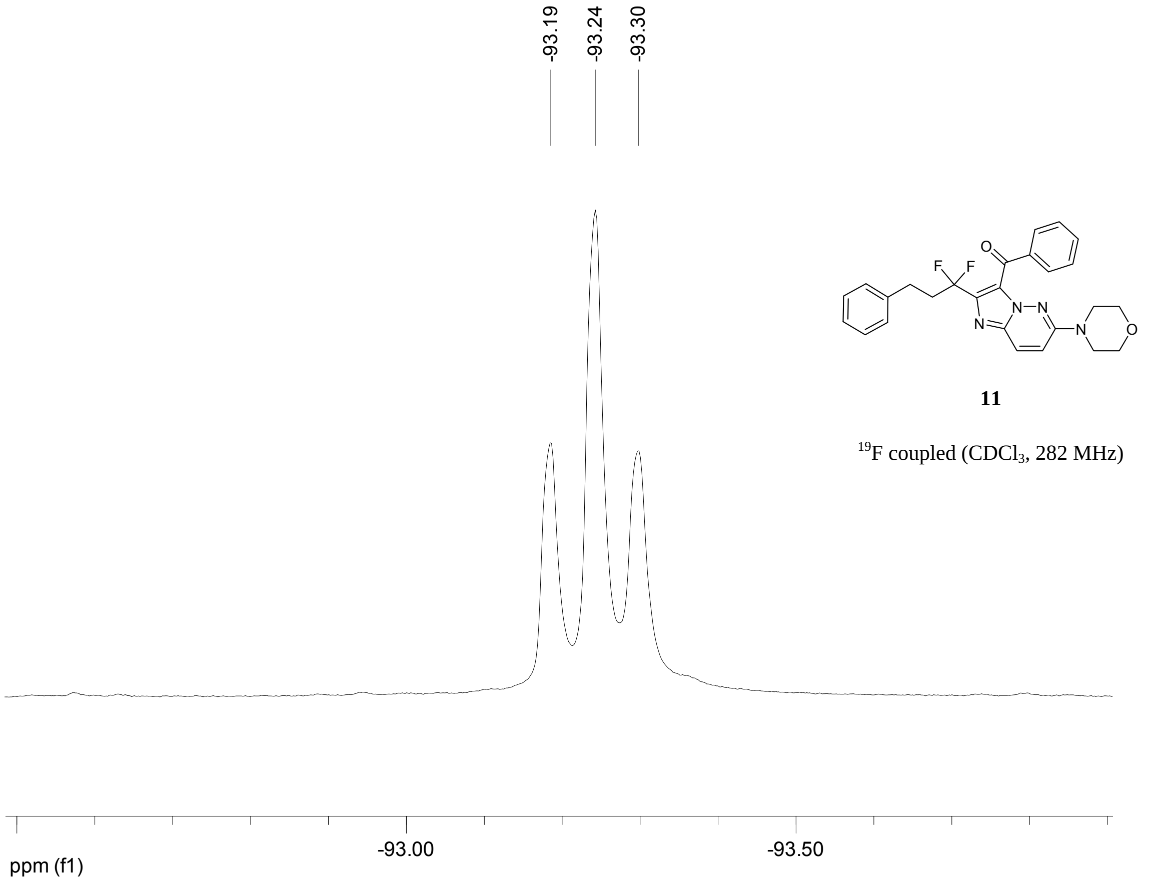

11

^{19}F coupled ($CDCl_3$, 282 MHz)

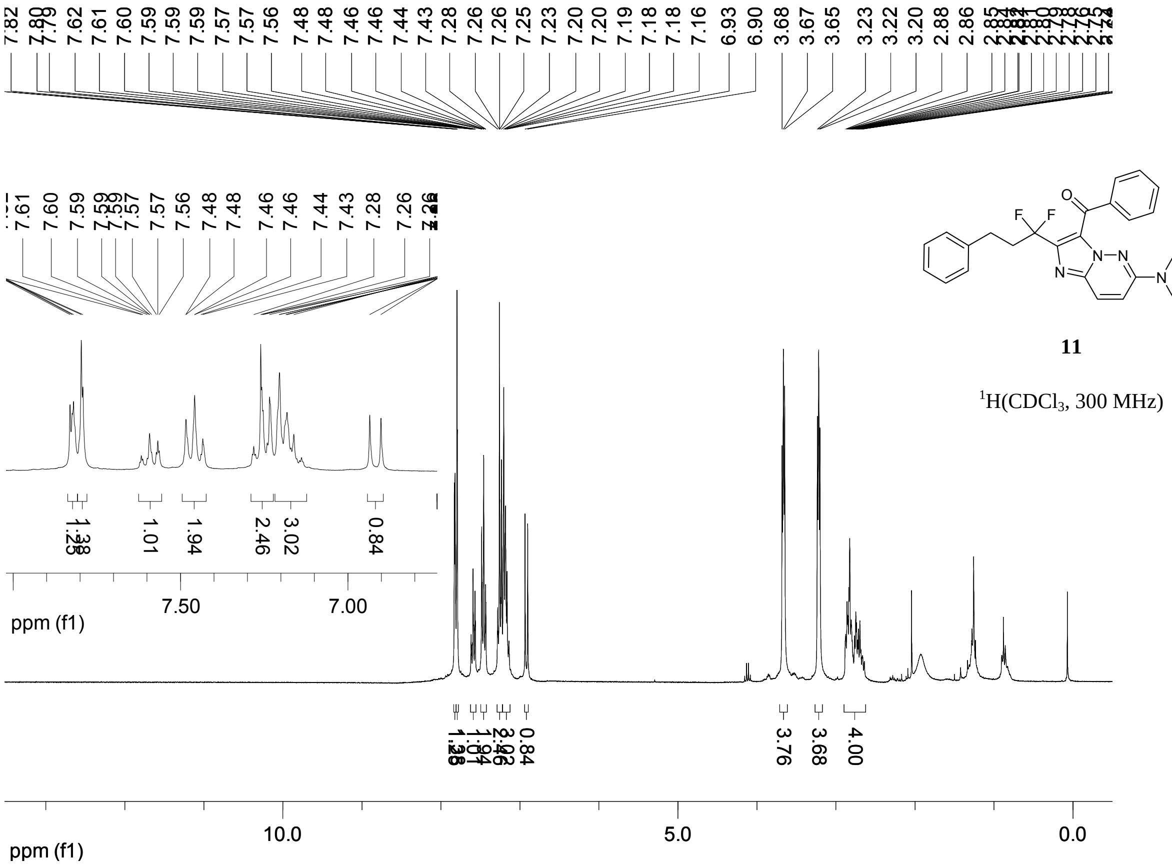

11

$^{1}H(CDCl_3, 300\ MHz)$

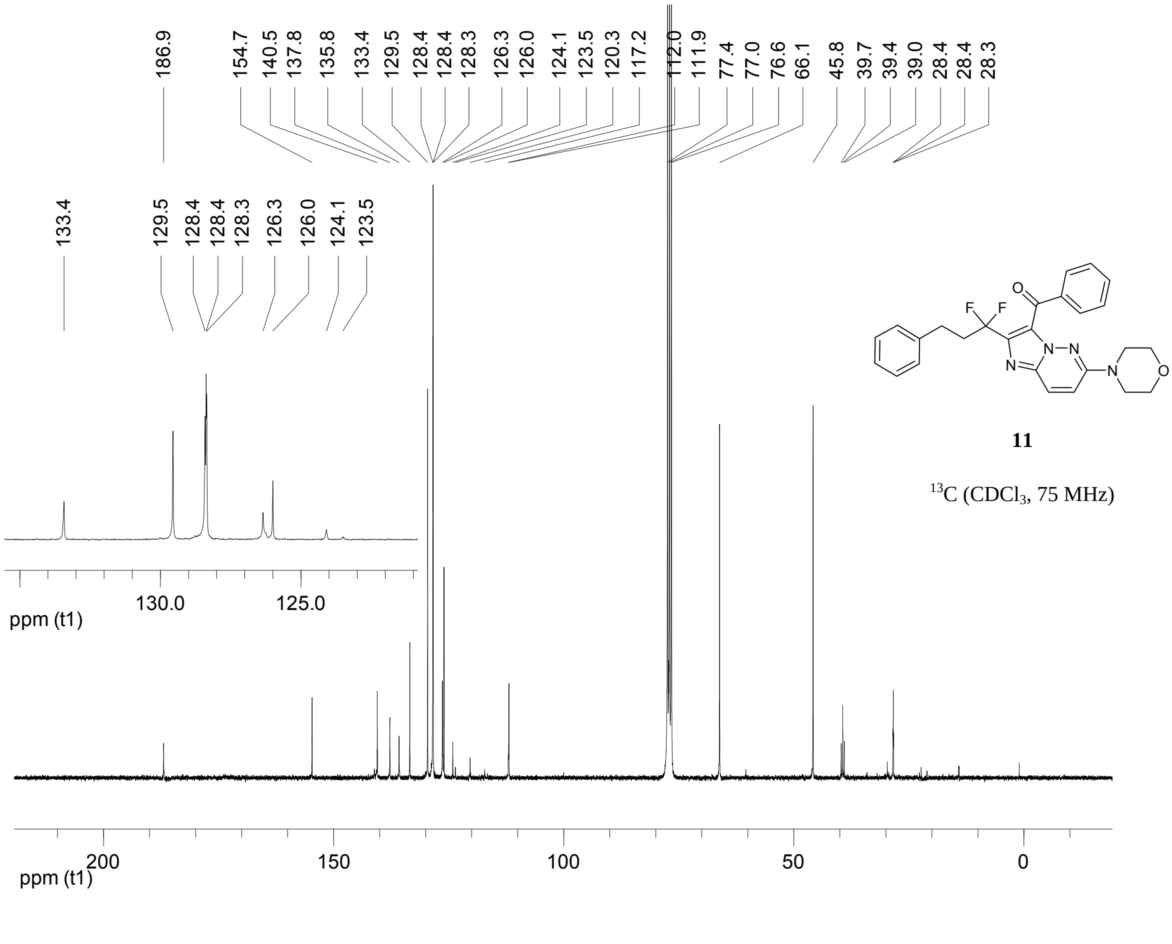

11

$^{13}C\ (CDCl_3, 75\ MHz)$
